# Supplementary material for: Ultra-large virtual screening unveils potent agonists of the neuromodulatory orphan receptor GPR139
Source: Nat Commun. 2025 Dec 9;17:129. doi: 10.1038/s41467-025-66845-y (PMC12775434; doi:10.1038/s41467-025-66845-y)
Supplement: Supplementary file 1 — Supplementary Information [file 41467_2025_66845_MOESM1_ESM.pdf]

Supporting Information:

Ultra-large virtual screening unveils potent agonists of the neuromodulatory orphan receptor GPR139

Israel Cabeza de Vaca[1,†], Boris Trapkov[2,†], Ling Shen[3,†], Duy Duc Vo[1], Xiaoqun Zhang[4], Yunting Yang[4], Mitra Pezeshki[2], Xuehan Zhang[3,5], Aljona Saleh[6], Frida Bällgren[6], Andrii V. Tarnovskiy[7], Dmytro S. Radchenko[7], Yurii S. Moroz[7,8,9], Hans Bräuner-Osborne[2], Per Svenningsson[4], Jan Kihlberg[10], Zhi-Jie Liu[3,5,*], Alexander Sebastian Hauser[2,*], Jens Carlsson[1,*]

[1] Science for Life Laboratory, Department of Cell and Molecular Biology, Uppsala University, SE-75124 Uppsala, Sweden

[2] Department of Drug Design and Pharmacology, University of Copenhagen, 2100 Copenhagen, Denmark

[3] iHuman Institute, ShanghaiTech University, Shanghai 201210, China

[4] Neuro Svenningsson, Department of Clinical Neuroscience, Karolinska Institute, SE-171 76 Stockholm, Sweden

[5] School of Life Science and Technology, ShanghaiTech University, Shanghai 201210, China

[6] Department of Pharmacy, SciLifeLab Drug Discovery and Development, Uppsala University, SE-751 23 Uppsala, Sweden

[7] Enamine Ltd., 02094 Kyiv, Ukraine

[8] Taras Shevchenko National University of Kyiv, Kyiv 01601, Ukraine

[9] Chemspace LLC, Kyiv 02094

[10] Department of Chemistry-BMC, Uppsala University, SE-75123 Uppsala, Sweden

[†]Contributed equally to this work

[*]To whom correspondence should be addressed: jens.carlsson@icm.uu.se, alexander.hauser@sund.ku.dk, liuzhj@shanghaitech.edu.cn

Keywords: Molecular docking, G protein-coupled receptor, structure-based drug design

Table of Contents

Supplementary Tables

1. Experimentally evaluated compounds 3
2. Pharmacological characterization of GPR139 agonists 5
3. Tanimoto similarities between virtual screening hits and known actives 6
4. Comparison of GPR139 agonist 7
5. AlphaFold3 protein-ligand complex predictions 8
6. Signaling signature of GPR139 with compound **1.1** 9
7. Evaluation of enantiopure compounds of **1.1** and **JNJ-63533054** (**JNJ**) 10
8. Cryo-EM data collection and GPR139-miniG$_{s/q}$ processing 11

Supplementary Figures

1. Pharmacological screening of predicted ligands from the virtual screening ... 12
2. Specificity testing of the GPR139 hits 13
3. Evaluation of screening hits at three concentrations 14
4. Pharmacological screening of compound **1** analogs 15
5. Specificity testing of compound **1** analogs 16
6. CRCs of compound **1** analogs 17
7. CRCs of series representative compound **1** analogs for IP$_1$ accumulation 18
8. Sample preparation and Cryo-EM data processing of the GPR139–compound **1.1(S)**–miniG$_{s/q}$–Nb35 complex 19
9. Structural comparison of GPR139 bound to compound **1.1(S)** and **JNJ-63533054** 20
10. AlphaFold3-predicted protein-ligand complex structures for five orphan GPCRs and GPR139 with small-molecule ligands 21
11. pLDDT of AlphaFold3-predicted protein-ligand complex structures 22
12. PAE of AlphaFold3-predicted protein-ligand complex structures 23
13. Evaluation of preferred configuration of compound **1.1** 24
14. Overview and controls of the two-step Ca^{2+} mobilization screening protocol for simultaneous identification of agonist and antagonists 25

Supplementary Methods

1. Synthesis of compounds **1-5** 26
2. General synthetic procedures for compounds **JNJ**, **1.1**, and **1.5** 29
3. Synthesis of **JNJ(S)** and **JNJ(R)** 30
4. Synthesis of compounds **1.1(S)**, **1.1(R), 1.5(S)**, and **1.5(R)** 32
5. HPLC chromatograms 34
6. NMR spectra 42

Supplementary References

Supplementary Tables

Supplementary Table 1. Experimentally evaluated compounds. The iteration 1 compounds were predicted from the structure-based virtual screen. In iteration 2 and 3, the scaffold represented by compound **1** was optimized.

Iteration	Compound	MW	Purity (%)	SMILES
1	1	341.14	95.65	CC(NC(=O)CC1=NOC(=N1)C2=CC(C)=C(C)S2)C=3C=CC=CC3
	2	348.15	100.00	COC(=O)C=1C=C(NC(=O)NC(C)C=2C=CC=C(F)C2F)C=CC1C
	3	322.16	99.40	CC(NC(=O)NC=1C=CC=C(C1)C=2C=CC=CC2)C3=NC(C)=NO3
	4	342.19	100.00	CC1=NOC(CNC(=O)C2(C)CCCCN2C(=O)C=3C=CC=CC3)=N1
	5[a]	333.17	100.00	C[C@@H](NC(=O)CN1C=NC=C(C=2C=CC=CC2)C1=O)C=3C=CC=CC3
	6	334.18	100.00	COCC(NC(=O)NC(C)C=1C=CC(F)=CC1F)C=2C=CC=CC2
	7	304.12	100.00	ClC1=CC=C(CNC(=O)N2CCCC=3C=CC=CC3C2)O1
	8	329.11	100.00	C[C@H](NC(=O)C1=CC=C(CN2C=C(Cl)C=N2)O1)C=3C=CC=CC3
	9	335.14	100.00	CN1CC(=O)N(C1=O)C=2C=CC=C(NC(=O)C3CC=4C=CC=CC34)C2
	10	332.44	100.00	C[C@H](NC(=O)CN(CC1CC1)C(=O)OC(C)(C)C)C=2C=CC=CC2
	11	324.82	100.00	CN(CC1=CC(Cl)=CN1C)C(=O)CCC(=O)C2=CC=CS2
	12	348.09	100.00	CC(NC(=O)C1CCN(C1=O)C=2C=CC=C(Cl)C2)C=3C=CSC3
	13	312.41	100.00	O=C(NC1CC21CCCC2)C3CCCN(C3=O)C=4C=CC=CC4
	14	327.17	100.00	CCOC=1C=CC(=CC1)C(=O)OCC(=O)N[C@H](C)C=2C=CC=CC2
	15	294.17	100.00	C[C@H](NC(=O)C=1C=CC=2C(C)=NN(C)C2N1)C=3C=CC=CC3
	16	337.14	100.00	C[C@H](NC(=O)C=1C=CC(=O)N(N1)C=2C=CC(F)=CC2)C=3C=CC=CC3
	17	333.18	100.00	C[C@H](NC(=O)C(=CC=1C=CC=CC1)N2N=NN=C2C)C=3C=CC=CC3
	18	266.13	100.00	C[C@H](NC(=O)C1=CN2C=CC=NC2=N1)C=3C=CC=CC3
	19	322.13	95.98	CC(O)CN(CC=1C=CC=CC1)C(=O)NCC2=CC=C(Cl)O2
	20	348.18	100.00	CC(C)(NC(=O)NC=1C=CC=CC1)C(=O)NC=2C=NC=3C=CC=CC3C2
	21	309.17	100.00	CC1=NC=2C=C(NC(=O)COC=3C=CC(C)=CC3)C=CC2N1C
	22	347.26	100.00	CC(CC(=O)N(CC1CCCO1)CC(=O)N2CCCC2)N3C=CC=C3
	23	345.03	96.80	CC=1SC=2N=CN(NC(=O)C3=CSC4=CN=CN34)C(=O)C2C1C
	24	346.11	98.68	O=C(CN1C=CN=C1)NC=2C=CC=C(C2)N3C(=O)C=4C=CC=CC4C3=O
	25	324.03	98.09	CN(CC1=NN=CN1)C2=NC(=NO2)C=3C=C(Cl)C=C(Cl)C3
	26	339.22	98.22	C[C@H](NC(=O)C1(CCOCC1)OCC=2C=CC=CC2)C=3C=CC=CC3
	27	318.17	96.21	CC=1NC(=NC1C(=O)N2CCC=3N=CC=CC23)C=4C=CC=C(C)C4
	28	348.19	96.81	O=C(NCC1CCC=CC1)NC=2C=NN(C2)C=3C=NC=4C=CC=CC4N3
	29	349.16	100.00	CCC=1OC=NC1C(=O)NC=2C=CC=C(C2)C(=O)NCC=3C=CC=CC3
	30	333.09	100.00	CN1C(SCC(=O)NC=2C(F)=CC=CC2F)=NC=3C=CC=CC13
	31	317.13	100.00	O=C(NC=1C=CN=C(N1)C=2C=CC=CC2)C3COC=4C=CC=CC34
	32	312.12	100.00	CC(NC(=O)C=1OC=CC1COC=2C=CC=CC2)C=3C=CON3
	33	348.09	100.00	FC=1C=CC(NC(=O)CCN2N=NC=3C=CC=CC3C2=O)=C(F)C1F
	34	319.17	93.76	CC(NC(=O)N1CCCCC1C=2C=NN(C)C2)C3=NC=CS3
	35	313.08	100.00	ClC=1C=CC=C(C1)N2C=C(N=N2)C(=O)NCC=3C=CC=NC3
	36	343.05	95.96	CC(=O)C=1SC(NC(=O)CC2=CN=CS2)=NC1C=3C=CC=CC3
	37	326.14	100.00	CC=1C=CC(=C(C)C1)N2C=C(NC(=O)NCC3=CC=CS3)C=N2
	38	347.07	100.00	CC=1N=NSC1C(=O)N2CCCC2C3=NC=4C=C(Cl)C=CC4N3
	39	345.16	100.00	CN1C=CC(CNC(=O)NC2=CC(=NN2C)C=3C=C(F)C=C(F)C3)=C1
	40	320.08	100.00	CN(CC1=NC=C(Cl)N1C)C2=NC=3C=C(C=CC3N2)[N+](=O)[O-]
	41	336.22	100.00	CC(C)N(C)C(=O)C=1C=C(NC(=O)C2CC=3C=CC=CC23)C=CC1C
	42	343.14	97.23	CN1N=C(C=C1NC(=O)NCC=2C=CC=CN2)C=3C=CC(F)=CC3F
	43	330.16	100.00	O=C(NC=1C=CN=C(OCC=2C=CC=CC2)C1)C3CC=4C=CC=CC34
	44	340.21	100.00	COCC1(CNC(=O)NCC=2C=CC=CC2)OCCC=3C=CC=CC31
	45	336.15	90.37	CCN1C(NC=2C=CN(C)N2)=NN=C1C3=NNC(=O)C=4C=CC=CC34
	46	345.08	100.00	FC=1C=CC=CC1NC(=O)CCN2C=NC=3C=C(Cl)C=CC3C2=O
	47	304.16	98.25	CC1CC=2N=CN(CC3=CSC(NC(=O)C)=N3)C2CC1C
	48	326.17	100.00	CC(C(O)CN1C=NC=2C=C(F)C(C)=CC2C1=O)C=3C=CC=CC3
	49	312.17	100.00	CC(NC(=O)NC=1C=CC=2C(=O)CCCC2C1)C3=CC=C(C)O3
	50	319.17	100.00	CC(NC(=O)N(C)CC=1C=CC=C(C1)N(C)C)C2=CSN=N2
	51	332.20	100.00	CC(NC(=O)C(C)SC1=NN=C(N1)C(C)(C)C)C=2C=CC=CC2
	52	298.16	100.00	CC1(C)COC=2C=C(NC(=O)NCC=3C=CC=NN3)C=CC21
	53	330.18	96.68	C[C@@H](NC(=O)CSC1=NN=C2CCCCCN12)C=3C=CC=CC3
	54	347.05	98.67	C[C@@H](NC(=O)CSC1=NN=C(S1)C(F)(F)F)C=2C=CC=CC2

	55	330.17	91.65	C[C@@H](NC(=O)CN1C=2CCCCCC2SC1=O)C=3C=CC=CC3
	56	310.17	97.94	CC=1C=CC(=CN1)C(C)(C)NC(=O)NC=2C=NC3=NC=CN3C2
	57	327.12	100.00	FC=1C=CC(NC(=O)CCN2N=NN(C=3C=CC=CC3)C2=O)=CC1
	58	340.03	98.57	FC(F)(F)C1=CSC(NC(=O)C=2C=CC=CC2N3C=NN=N3)=N1
	59	322.16	100.00	CC(C(=O)NC=1C=CC(OC=2C=CC(C)=CC2)=NC1)N3C=CC=N3
	60	334.04	100.00	N[C@@H](CC(=O)O)C1=NOC(=N1)C=2C=C(ON2)C=3C=CC(Cl)=CC3
	61	318.17	100.00	CC=1C=CC(C(=O)NC=2C=CC=C(C2)C3=NN=CN3)=C4CCCC41
	62	323.17	98.45	CCN(C(=O)C1=CC(CC=2C=CC=CC2)=NN1)C=3C=CC(F)=CC3
	63	346.08	100.00	C[C@@H](NC(=O)CSC1=NC=2C=C(Cl)C=NC2N1)C=3C=CC=CC3
	64	319.03	100.00	ClC1=CC=C(CNC(=O)C=2C=CN=C(C2)N3C=NC=N3)S1
	65	347.05	100.00	CC1=NOC(=N1)C=2C=CC(C)=C(NC(=O)CSC3=NN=CS3)C2
	66	343.09	100.00	ClC=1C=CC(OC=2C=CC(NC(=O)CCN3C=NC=N3)=CN2)=CC1
	67	345.18	97.58	CC(C(=O)NC=1C=CC=C(C1)C2=NC=3C=CC=CC3N2C)N4C=CC=N4
	68	322.14	100.00	CC=1C=CC(=CC1)C2=NC(CC(=O)NCC=3C=CC=CC3)=CS2
	1.1	327.41	100.00	CC(C1=CC=CC=C1)NC(CC2=NOC(C3=CSC=C3C)=N2)=O
	1.2	337.38	99.67	COC1=C(C2=NC(CC(NC(C3=CC=CC=C3)C)=O)=NO2)C=CC=C1
	1.3	321.38	97.56	CC(C1=CC=CC=C1)NC(CC2=NOC(C3=CC(C)=CC=C3)=N2)=O
	1.4	327.41	99.78	CC(C1=CC=CC=C1)NC(CC2=NOC(C3=CSC(C)=C3)=N2)=O
	1.5	312.33	100.00	CC(C1=CC=CC=C1)NC(CC2=NOC(C3=CON=C3C)=N2)=O
	1.6	311.34	100.00	CC(C1=CC=CC=C1)NC(CC2=NOC(C3=CC=C(O3)C)=N2)=O
	1.7	338.37	99.21	COC1=NC=CC(C2=NC(CC(NC(C3=CC=CC=C3)C)=O)=NO2)=C1
	1.8	313.38	98.58	CC(C1=CC=CC=C1)NC(CC2=NOC(C3=CC=CS3)=N2)=O
	1.9	327.41	100.00	CC(C1=CC=CC=C1)NC(CC2=NOC(C3=CC=C(S3)C)=N2)=O
	1.10	337.38	100.00	COC1=CC(C2=NC(CC(NC(C3=CC=CC=C3)C)=O)=NO2)=CC=C1
	1.11	327.41	97.32	CC(C1=CC=CC=C1)NC(CC2=NOC(C3=CC(C)=CS3)=N2)=O
	1.12	341.44	98.67	CCC1=CC=C(C2=NC(CC(NC(C3=CC=CC=C3)C)=O)=NO2)S1
	1.13	322.37	90.05	CC(C1=CC=CC=C1)NC(CC2=NOC(C3=NC(C)=CC=C3)=N2)=O
	1.14	321.38	98.36	CC(C1=CC=CC=C1)NC(CC2=NOC(C3=CC=C(C)C=C3)=N2)=O
	1.15	328.40	99.91	CC(C1=CC=CC=C1)NC(CC2=NOC(C3=CSC(C)=N3)=N2)=O
	1.16	325.37	100.00	CC(C1=CC=CC=C1)NC(CC2=NOC(C3=CC(C)=NN3C)=N2)=O
	1.17	322.37	100.00	CC(C1=CC=CC=C1)NC(CC2=NOC(C3=NC=CC(C)=C3)=N2)=O
	1.18	337.38	98.98	COC1=CC=C(C2=NC(CC(NC(C3=CC=CC=C3)C)=O)=NO2)C=C1
2	1.19	322.37	100.00	CC(C1=CC=CC=C1)NC(CC2=NOC(C3=CC(C)=NC=C3)=N2)=O
	1.20	335.41	99.93	CC(C1=CC=CC=C1)NC(CC2=NOC(C3=CC(C)=C(C)C=C3)=N2)=O
	1.21	367.43	99.61	CC(C1=CC=CC=C1)NC(CC2=NOC(C3=C(C)N4C=CN=C4S3)=N2)=O
	1.22	362.39	99.61	CC(C1=CC=CC=C1)NC(CC2=NOC(C3=CC4=C(OC(C)=N4)C=C3)=N2)=O
	1.23	312.33	99.01	CC(C1=CC=CC=C1)NC(CC2=NOC(C3=NOC(C)=C3)=N2)=O
	1.24	322.37	100.00	CC(C1=CC=CC=C1)NC(CC2=NOC(C3=CC(C)=CN=C3)=N2)=O
	1.25	312.33	97.56	CC(C1=CC=CC=C1)NC(CC2=NOC(C3=CN=C(O3)C)=N2)=O
	1.26	338.37	97.35	COC1=NC=C(C2=NC(CC(NC(C3=CC=CC=C3)C)=O)=NO2)C=C1
	1.27	339.40	91.03	CCN1N=C(C2=NC(CC(NC(C3=CC=CC=C3)C)=O)=NO2)C=C1C
	1.28	355.42	99.51	CC(C1=CC=CC=C1)NC(CC2=NOC(C3=CC=C(C(C)=O)S3)=N2)=O
	1.29	312.33	95.71	CC(C1=CC=CC=C1)NC(CC2=NOC(C3=COC(C)=N3)=N2)=O
	1.30	312.33	93.15	CC(C1=CC=CC=C1)NC(CC2=NOC(C3=CC(C)=NO3)=N2)=O
	1.31	336.40	100.00	CC(C1=CC=CC=C1)NC(CC2=NOC(C3=NC(C)=C(C)C=C3)=N2)=O
	1.32	322.37	99.01	CC(C1=CC=CC=C1)NC(CC2=NOC(C3=CN=C(C)C=C3)=N2)=O
	1.33	327.35	98.24	CC(C1=CC=CC=C1)NC(CC2=NOC(CC3=NON=C3C)=N2)=O
	1.34	328.40	96.73	CC(C1=CC=CC=C1)NC(CC2=NOC(C3=CN=C(S3)C)=N2)=O
	1.35	338.37	100.00	COC1=CN=C(C2=NC(CC(NC(C3=CC=CC=C3)C)=O)=NO2)C=C1
	1.36	393.47	95.77	COC1=CC2=C(C=C(C3=NC(CC(NC(C4=CC=CC=C4)C)=O)=NO3)S2)C=C1
	1.37	367.41	100.00	COC1=CC=C(OCC2=NC(CC(NC(C3=CC=CC=C3)C)=O)=NO2)C=C1
	1.38	312.39	100.00	CC(NC(=O)CN1C=C(N=N1)C2=CC=CS2)C=3C=CC=CC3
	1.39	343.40	100.00	COC1=CSC(=C1)C2=NC(CC(=O)NC(C)C=3C=CC=CC3)=NO2
	1.40	361.40	96.96	CC(NC(=O)CC1=NOC(=N1)C2=CC3=NC=C(C)N3C2)C=4C=CC=CC4
3	1.41	355.45	100.00	CC(C)C1=CC=C(S1)C2=NC(CC(=O)NC(C)C=3C=CC=CC3)=NO2
	1.42	326.35	91.91	CCC1=NC(=CO1)C2=NC(CC(=O)NC(C)C=3C=CC=CC3)=NO2
	1.43	328.39	90.90	CC(NC(=O)CC1=NOC(=N1)C=2C=C(C)SN2)C=3C=CC=CC3
	1.44	312.39	100.00	CC(NC(=O)CC1=COC(=N1)C2=CC=CS2)C=3C=CC=CC3

[a] Compound **5** was first obtained as the R-enantiomer (EC_{50} = 3.6 μM). The S-enantiomer was subsequently custom synthesized and showed greater activity in the calcium mobilization assay (EC_{50} = 540 nM), which agreed with the docking predictions.

Supplementary Table 2. Pharmacological characterization of GPR139 agonists.[a]

Compound	pEC50 *Ca2+*	Emax (%) *Ca2+*	pEC50 *IP1*	Emax (%) *IP1*
**Lundbeck Cmp 1a**	6.7 ± 0.09	108 ± 3	6.3 ± 0.07	101 ± 12
**1**	6.8 ± 0.1	94 ± 3	6.1 ± 0.1	114 ± 5
**2**	6.5 ± 0.07	83 ± 6	5.8 ± 0.09 *(p<0.0002)	85 ± 8 *
**3**	5.6 ± 0.2 *(p<0.0001)	70 ± 3 *(p<0.0005)	4.3 ± 0.09 [b],*(p<0.0001)	43 ± 1 [c],*(p<0.0013)
**4**	5.4 ± 0.08 *(p<0.0001)	100 ± 7	4.9 ± 0.02 [b],*(p<0.0001)	95 ± 7 [c],*
**5**	5.4 ± 0.1 *(p<0.0001)	88 ± 4	4.6 ± 0.06 [b],*(p<0.0001)	59 ± 5 [c],*(p=0.0259)
**1.1**	7.3 ± 0.1 *(p=0.0172)	125 ± 12	7.3 ± 0.09 *(p<0.0001)	91 ± 10
**1.2**	7.3 ± 0.07 *(p=0.0172)	116 ± 6	6.3 ± 0.06	100 ± 9
**1.3**	7.0 ± 0.1	109 ± 6		
**1.4**	7.0 ± 0.1	114 ± 6		
**1.5**	6.9 ± 0.1	103 ± 6	6.3 ± 0.07	102 ± 15
**1.6**	6.8 ± 0.09	112 ± 10		
**1.7**	6.7 ± 0.05	113 ± 9	5.8 ± 0.05 *(p=0.0002)	117 ± 14
**1.8**	6.7 ± 0.1	107 ± 2		
**1.9**	6.7 ± 0.1	86 ± 8		
**1.10**	6.7 ± 0.03	111 ± 5		
**1.11**	6.7 ± 0.1	105 ± 10		
**1.12**	6.4 ± 0.2	93 ± 4		
**1.13**	6.4 ± 0.06	95 ± 10		
**1.14**	6.4 ± 0.2	89 ± 15		
**1.15**	6.3 ± 0.08	105 ± 4		
**1.16**	6.2 ± 0.2	93 ± 11	5.6 ± 0.03 *(p=0.0001)	118 ± 5
**1.17**	6.1 ± 0.1 *(p=0.0172)	98 ± 3		
**1.18**	6.0 ± 0.2 *(p=0.0021)	72 ± 5 *(p=0.0012)		
**1.19**	6.0 ± 0.1 *(p=0.0021)	109 ± 10		
**1.20**	5.9 ± 0.1 *(p=0.0002)	86 ± 11		
**1.21**	5.9 ± 0.1 *(p=0.0002)	88 ± 6		
**1.22**	5.8 ± 0.2 *(p=0.0001)	99 ± 2		
**1.23**	5.8 ± 0.2 *(p=0.0001)	95 ± 3		
**1.24**	5.8 ± 0.2 *(p=0.0001)	91 ± 4		
**1.25**	5.7 ± 0.08 *(p=0.0001)	97 ± 3		
**1.26**	5.7 ± 0.1 *(p=0.0001)	94 ± 9		
**1.27**	5.7 ± 0.2 *(p=0.0001)	96 ± 6		
**1.28**	5.6 ± 0.08 *(p=0.0001)	66 ± 3 *(p=0.0001)		
**1.29**	5.6 ± 0.2 *(p=0.0001)	90 ± 8		
**1.30**	5.6 ± 0.05 *(p=0.0001)	79 ± 3 *(p=0.0241)		
**1.31**	5.5 ± 0.2 *(p=0.0001)	86 ± 5		
**1.32**	5.5 ± 0.2 *(p=0.0001)	82 ± 9		
**1.33**	5.4 ± 0.08 *(p=0.0001)	88 ± 2		
**1.34**	5.4 ± 0.2 *(p=0.0001)	90 ± 6		
**1.35**	5.3 ± 0.2 *(p=0.0001)	101 ± 5		
**1.38**	5.9 ± 0.1 *(p=0.0001)	82 ± 7		
**1.39**	6.5 ± 0.09	123 ± 14		
**1.40**	5.5 ± 0.01 *(p=0.0001)	86 ± 7		
**1.41**	5.8 ± 0.2 *(p=0.0001)	67 ± 4 *(p=0.0001)		
**1.42**	5.7 ± 0.2 *(p=0.0001)	78 ± 3 *(p=0.0162)		
**1.43**	5.8 ± 0.2 *(p=0.0001)	102 ± 7		

[a]Ca^{2+} mobilization and IP_1 accumulation was measured in CHOk1 cells stably expressing GPR139. Responses are normalized to that of the reference **Lundbeck Cmp 1a**. Data represent mean ± SEM of at least three independent experiments performed in triplicates. [b] CRCs were incomplete; the value is estimated by simulating a CRC with E_{max} constrained to 100%. [c] Measured response at 32 μM. * Significant difference relative to **Lundbeck Cmp 1a** as determined by one-way ANOVA with Dunnett's multiple comparisons test at $p < 0.05$.

Supplementary Table 3. Tanimoto similarities between virtual screening hits and known actives. Tanimoto coefficients (ECFP4-based fingerprints) for comparisons to the active compounds extracted from ChemBL database for GPR139 (EC_{50} < 10 µM).

IDs	Structure: Hit from virtual screen	ChEMBL ID	Structure: ChEMBL ligand	Tanimoto coefficient
1	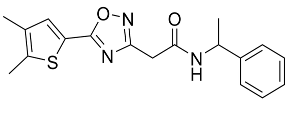	CHEMBL5432850	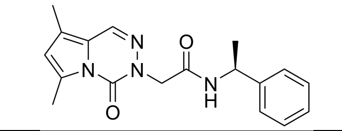	0.39
2	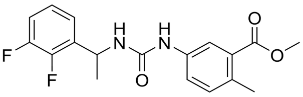	CHEMBL4782204	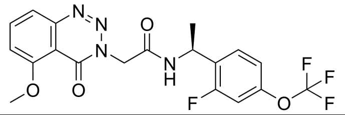	0.28
3	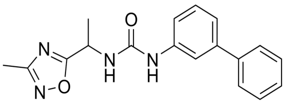	CHEMBL4781160	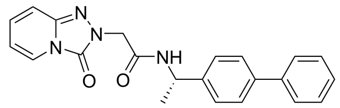	0.28
4	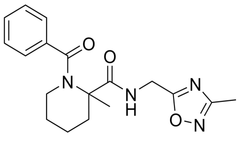	CHEMBL3633718	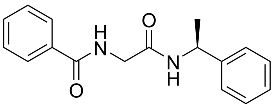	0.22
5	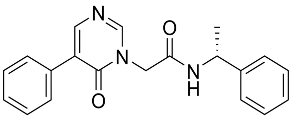	CHEMBL4786641	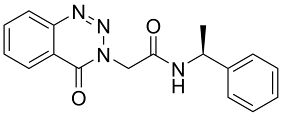	0.49

Supplementary Table 4. Comparison of GPR139 agonists. pEC_{50} and E_{max} values for Ca^{2+} mobilization and IP_1 accumulation of compound **1.1** and GPR139 reference agonists.

	**Ca^{2+} mobilization[a]**		**IP_1 accumulation[a]**	
**Compound**	**pEC_{50}**	**E_{max} (%)**	**pEC_{50}**	**E_{max} (%)**
**1.1**	7.3 ± 0.1	125 ± 12	7.3 ± 0.09	91 ± 10
**Lundbeck Cmp 1a**	6.7 ± 0.09 *(p=0.0009)	108 ± 3	6.3 ± 0.07 *(p=0.0001)	101 ± 12
**JNJ-63533054**	7.2 ± 0.06	100 ± 2	6.7 ± 0.05 *(p=0.0005)	157 ± 16 *(p=0.0059)
**TAK-041**	7.2 ± 0.05	100 ± 3	7.1 ± 0.1	101 ± 9

[a]Responses were measured in CHOk1 cells stably expressing GPR139. Data represent mean ± SEM of at least three independent experiments performed in triplicates. E_{max} is normalized to that of **Lundbeck Cmp 1a**. * Significant difference relative to compound **1.1** as determined by one-way ANOVA with Dunnett's multiple comparisons test at $p < 0.05$.

Supplementary Table 5. AlphaFold3 predictions of receptor-ligand complexes**.** Comparison of AlphaFold3 complex predictions with experimentally determined structures of orphan GPCRs in complex with small-molecule ligands.

**Receptor (PDB ID)**	**Ligand RMSD (Å)**	**Correctly predicted contacts[a]**	**Number of contacts**	
			**AF3**	**Experimental**
GPR139 (9M42)	2.7	13 (32 %)	71	41
GPR84 (8J19)	3.4	14 (21 %)	78	66
GPR132 (8HVI)	18.8	0 (0 %)	33	41
GPR61 (8TB7)	20.3	0 (0 %)	75	63
GPR6 (8T1V)	6.3	0 (0 %)	43	81
GPR35 (8H8J)	5.7	1 (2 %)	71	57

[a]The number of contacts is measured as the total number of receptor heavy atoms within a 4 Å distance of each ligand heavy atom.

Supplementary Table 6. Signaling signature of GPR139 with compound **1.1**.

	**Compound potency and E_{max}[a]**							
	**1.1(S)**		**1.1(R)**		**JNJ(S)**		**JNJ(R)**	
	**pEC_{50}**	**E_{max} (%)**	**pEC_{50}**	**E_{max} (%)**	**pEC_{50}**	**E_{max} (%)**	**pEC_{50}**	**E_{max} (%)**
**Gi2**	7.3 ± 0.06<br>****	95 ± 5<br>****	6.1 ± 0.1<br>*	88 ± 11<br>****	7.3 ± 0.05<br>*	109 ± 1<br>***	5.4 ± 0.04<br>*	86 ± 4<br>***
**Gi3**	7.3 ± 0.003<br>****	45 ± 3<br>****	6.1 ± 0.1<br>*	37 ± 1<br>****	7.0 ± 0.1<br>***	56 ± 4<br>****	5.4 ± 0.06<br>*	35 ± 6<br>***
**GoA**	7.2 ± 0.03<br>***	96 ± 3<br>****	6.1 ± 0.07<br>*	75 ± 3<br>***	7.1 ± 0.05<br>**	114 ± 2<br>****	5.3 ± 0.07<br>*	79 ± 1<br>**
**Gq**	6.7 ± 0.06<br>*****	35 ± 3<br>****	5.6 ± 0.07<br>**	21 ± 3<br>****	6.8 ± 0.06<br>***	42 ± 3<br>****	5.0 ± 0.07<br>*	22 ± 8<br>****
**G12**	7.9 ± 0.05<br>******	54 ± 3<br>****	6.1 ± 0.2<br>*	51 ± 4<br>**	7.6 ± 0.1<br>****	57 ± 1<br>****	5.4 ± 0.04<br>*	48 ± 3<br>**
**Ca^{2+}**	7.9 ± 0.1<br>******	146 ± 16<br>******	6.4 ± 0.2<br>***	139 ± 24<br>*******	7.8 ± 0.3<br>*****	133 ± 14<br>*****	5.9 ± 0.2<br>***	101 ± 18<br>****
**Arrestin-3**	7.1 ± 0.1<br>***	96 ± 1<br>****	5.6 ± 0.2<br>**	81 ± 5<br>***	6.8 ± 0.1<br>***	100 ± 4<br>****	4.8 ± 0.1[b]<br>****	63 ± 11[c]<br>**

[a]Activation of diverse G proteins, Ca^{2+} mobilization, and arrestin-3 recruitment was measured in HEK293A cells transiently expressing GPR139. Data represent mean ± SEM of at least three independent experiments performed in triplicates. E_{max} is normalized to that of **JNJ-63533054** (**JNJ(S)**).
[b] CRCs were incomplete; the value is estimated by simulating a CRC with E_{max} constrained to 100%.
[c] Measured response at 32 µM. Black * indicates a significant difference ($p<0.05$) relative to the corresponding R enantiomer at the respective signaling pathway. Brown * indicates a significant difference ($p<0.05$) relative to **JNJ (R)** at the respective signaling pathway. **1.1 (S)** responses were not of significant difference ($p<0.05$) relative to **JNJ (S)** responses at the individual signaling pathways. Magenta *, blue *, red *, yellow *, green *, purple *, and turquoise * indicate significant differences ($p<0.05$) of the respective compound signaling pathway response relative to its response at G_{i2}, G_{i3}, G_{oA}, G_q, G_{12}, Ca^{2+}, and arrestin-3, respectively. Significant differences were statistically determined by two-way ANOVA with Tukey's multiple comparisons test at $p < 0.05$.

Supplementary Table 7. Evaluation of enantiopure compounds of **1.1** and **JNJ-63533054** (**JNJ**).

Compound	pEC_{50} [a]	E_{max} (%)[a]
**1.1(S)**	8.1 ± 0.05	121 ± 11
**1.1(R)**	6.5 ± 0.05 *(p<0.0001)	112 ± 1
**JNJ(S)**	7.8 ± 0.1	119 ± 6
**JNJ(R)**	5.5 ± 0.2 *(p<0.0001)	99 ± 5
**Lundbeck Cmp 1a**	6.8 ± 0.09 *(p<0.0001)	108 ± 4

[a]pEC_{50} and E_{max} values for Ca^{2+} mobilization were determined in CHOk1 cells stably expressing GPR139. Data represent mean ± SEM of at least three independent experiments performed in triplicates. E_{max} is normalized to that of **Lundbeck Cmp 1a**. * Significant difference relative to compound **1.1(S)** as determined by one-way ANOVA with Dunnett's multiple comparisons test at $p < 0.05$.

Supplementary Table 8. Cryo-EM data collection and GPR139-miniG$_{s/q}$ processing.

Data collection and processing	GPR139-miniG$_{s/q}$
Magnification	130,000
Voltage (kv)	300
Electron exposure (e-/Å^2)	60
Defocus range(μm)	-1.2 ~ -2.0
Pixel size (Å)	0.96
Symmetry imposed	C1
Final particles	138,265
Map resolution	3.2
FSC threshold	0.143
**Refinement**	
Initial model used (PDB code)	7VUH
**Map sharping**	
B factor (Å)	114.0
**Model composition**	
Non-hydrogen atoms	8177
Protein residues	1027
Ligand	1
**B-factors**	
Protein	78.38
Ligand	84.94
**R.M.S. deviations**	
Bond lengths (Å)	0.005
Bond angles (°)	0.68
**Validation**	
MolProbity score	1.65
Clash score	8.78
Poor rotamers (%)	0.11
**Ramachandran plot**	
Favored (%)	96.93
Allowed (%)	3.07
Disallowed (%)	0.00
EMD code	EMD-63614
PDB code	9M42

Supplementary Figures

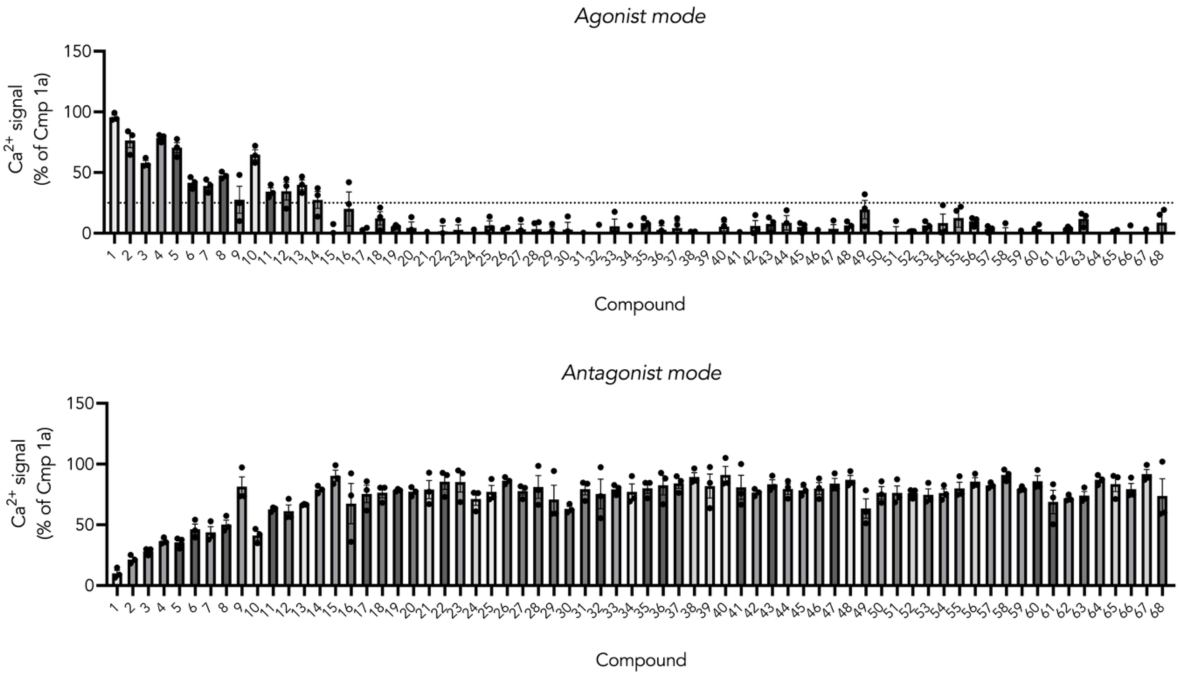

Supplementary Figure 1. Pharmacological screening of predicted ligands from the virtual screening. Two-step screening protocol. Ca^{2+} mobilization was measured in a stable GPR139-CHOk1 cell line. Top graph, agonist mode – Ca^{2+} mobilization was measured immediately after addition of screening compounds at 10 µM. Bottom graph, antagonist mode – followed 20 min preincubation with the screening compounds, Ca^{2+} mobilization was measured after an addition of EC_{80} concentration (800 nM) of reference agonist **Lundbeck Cmp 1a**. Data represent mean ± SEM of three independent experiments performed in triplicates and are normalized to buffer (0%) and 10 µM **Lundbeck Cmp 1a** (100%).

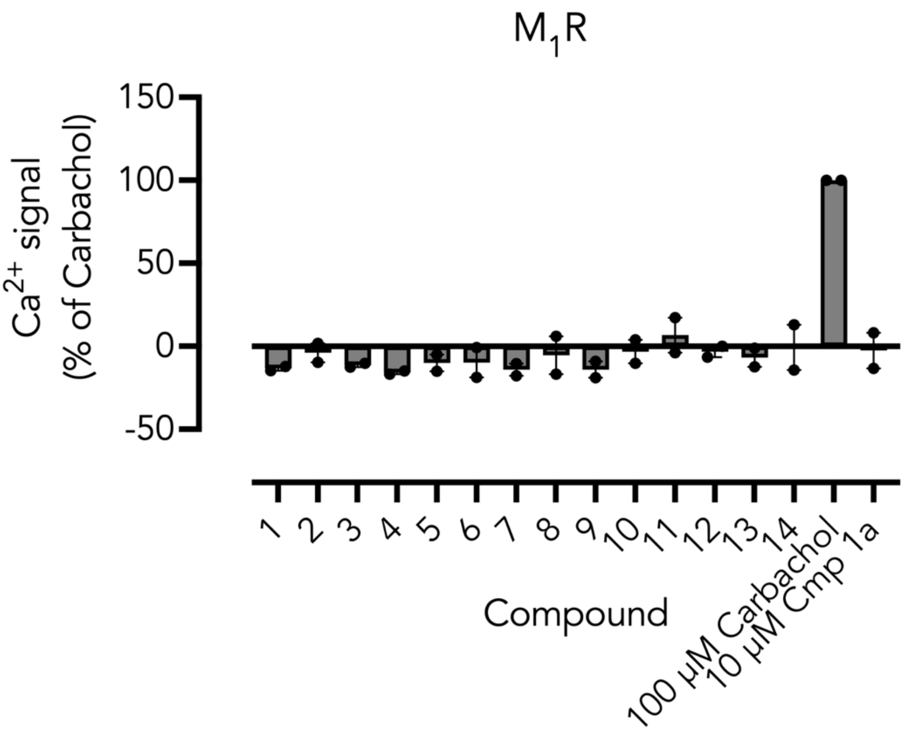

Supplementary Figure 2. Specificity testing of the GPR139 hits. The response specificity of the agonist hits for GPR139 was examined by counter screening at 10 µM for their ability to stimulate Ca^{2+} mobilization by M_1R in a stable M_1R-CHOk1 cell line. Data represent mean ± SEM of two independent experiments performed in duplicates and are normalized to buffer (0%) and 100 µM carbachol (100%).

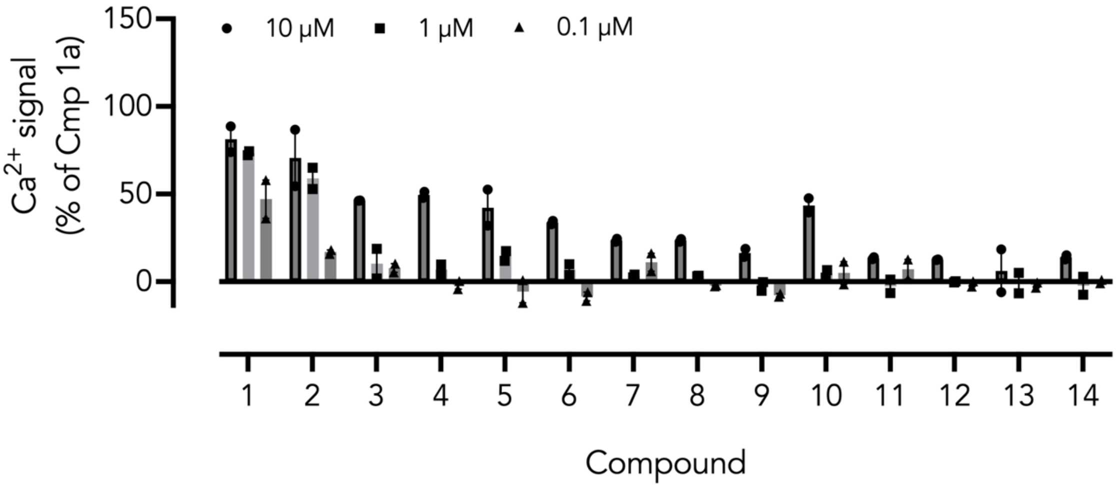

Supplementary Figure 3. Evaluation of screening hits at three concentrations. The GPR139 hits were tested with three decreasing concentrations to examine their activity in stimulating Ca^{2+} mobilization by GPR139 in a stable GPR139-CHOk1 cell line. Data represent mean ± SEM of two independent experiments performed in duplicates and are normalized to buffer (0%) and 10 μM **Lundbeck Cmp 1a** (100%).

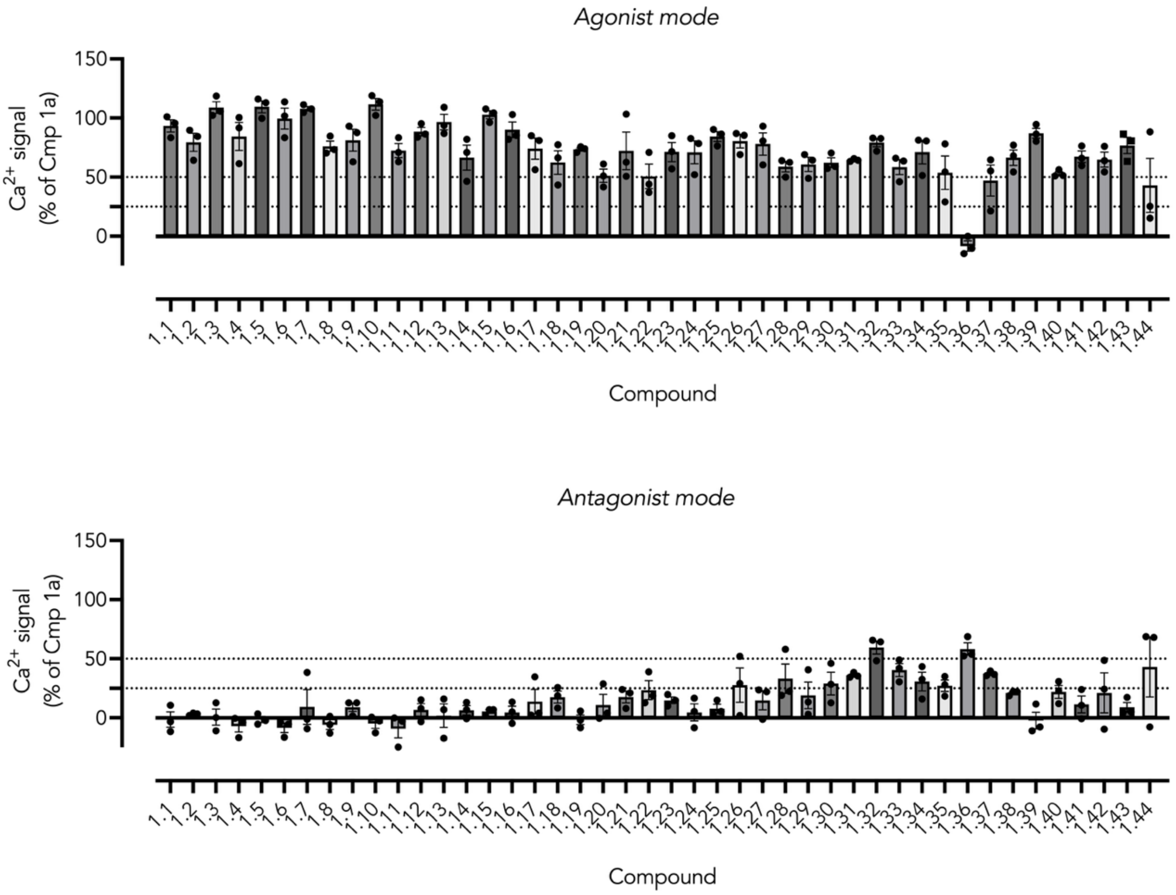

Supplementary Figure 4. Pharmacological screening of compound **1** analogs. Using a two-step screening protocol, analogs of compound **1** were screened at 10 µM for their ability to stimulate Ca^{2+} mobilization by GPR139 in a stable GPR139-CHOk1 cell line (Top graph: agonist mode, Bottom graph: antagonist mode). Data represent mean ± SEM of three independent experiments performed in triplicates and are normalized to buffer (0%) and 10 µM **Lundbeck Cmp 1a** (100%).

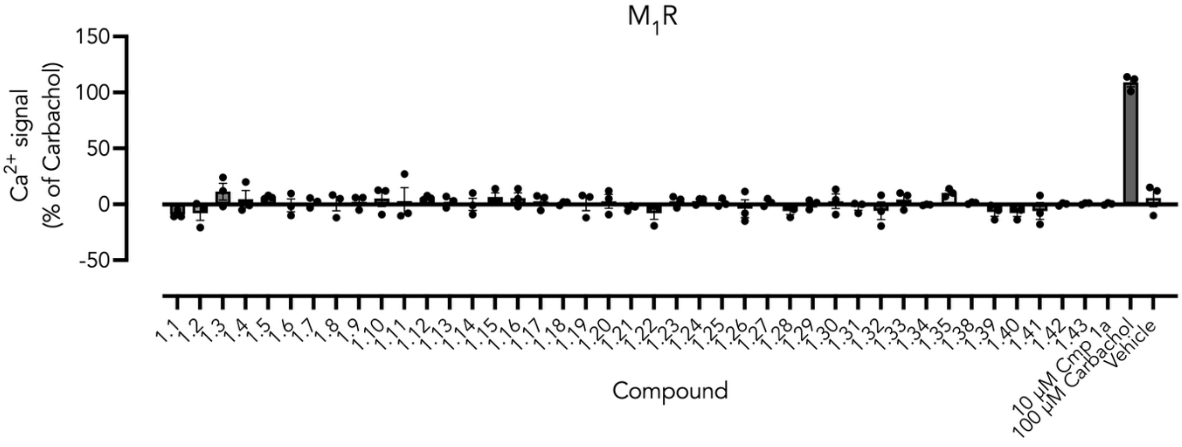

Supplementary Figure 5. Specificity testing of compound **1** analogs. The response specificity of compound **1** analogs for GPR139 was examined by counter screening at 10 μM for their ability to stimulate Ca^{2+} mobilization by M_1R in a stable M_1R-CHOk1 cell line. Data represent mean ± SEM of three independent experiments performed in triplicates and are normalized to buffer (0%) and 100 μM carbachol (100%).

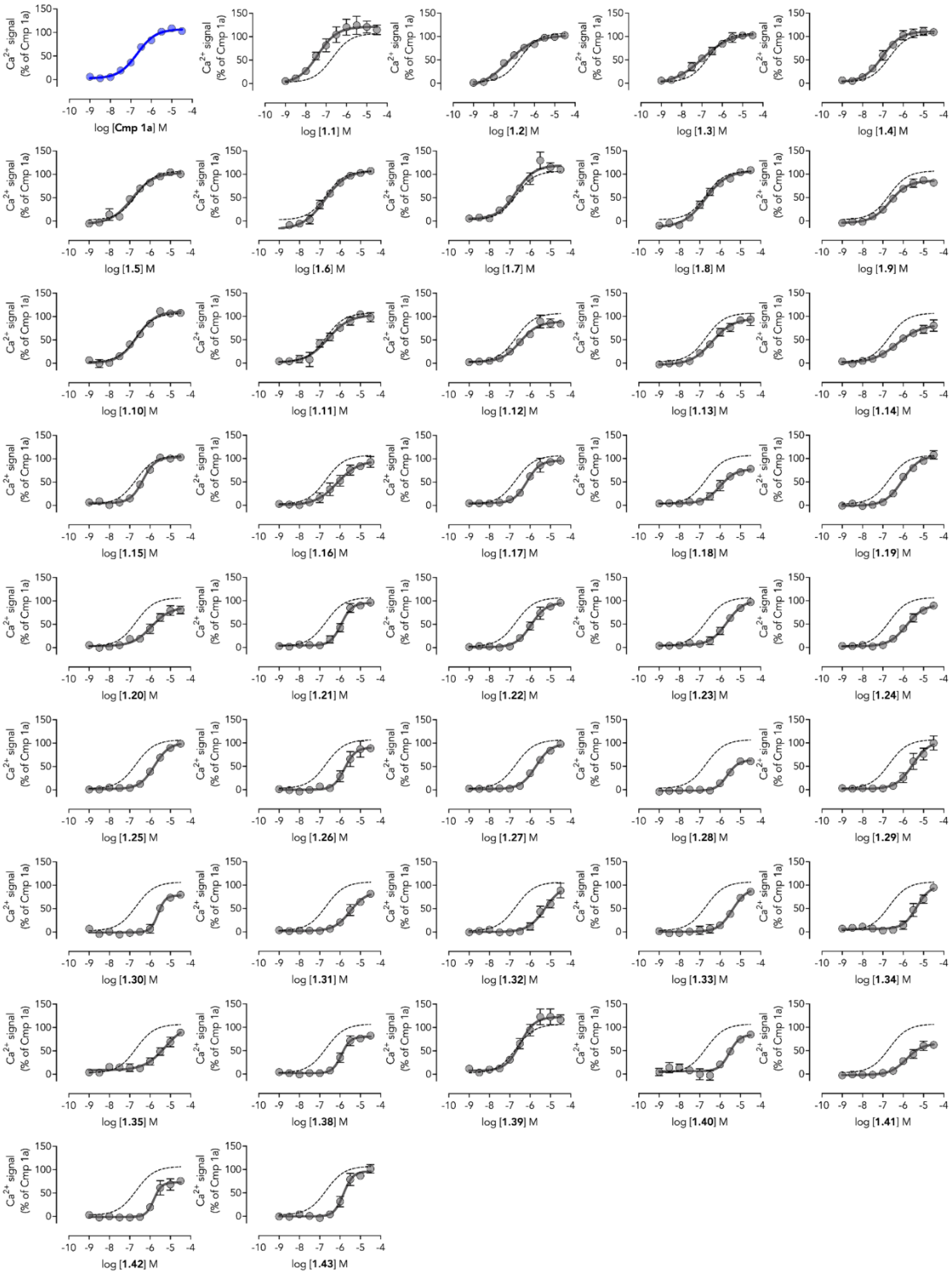

Supplementary Figure 6. CRCs of compound **1** analogs. Compound **1** analogs were pharmacologically characterized for their ability to stimulate intracellular Ca^{2+} mobilization by GPR139 in a stable GPR139-CHOk1 cell line. The dotted concentration-response curve corresponds to the reference **Lundbeck Cmp 1a**. Data represent mean ± SEM of at least three independent experiments performed in triplicates and are normalized to buffer (0%) and 10 µM **Lundbeck Cmp 1a** (100%).

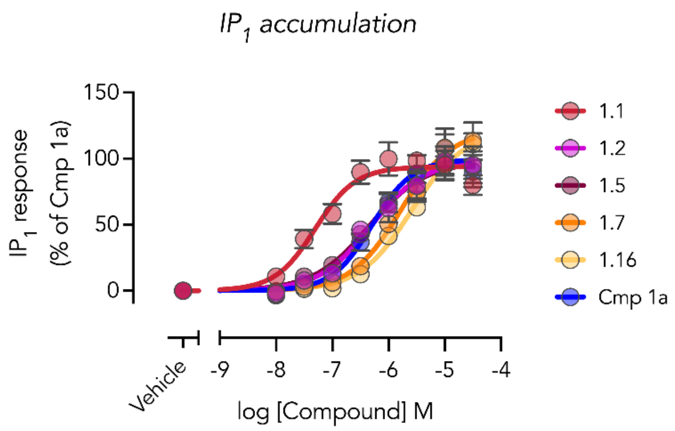

Supplementary Figure 7. CRCs of series representative compound **1** analogs for IP_1 accumulation. Series representative analogs of compound **1** were pharmacologically characterized for their ability to stimulate inositol monophosphate (IP_1) accumulation by GPR139 in a stable GPR139-CHOk1 cell line. Data represent mean ± SEM of at least three independent experiments performed in triplicates and are normalized to buffer (0%) and 10 μM **Lundbeck Cmp 1a** (100%).

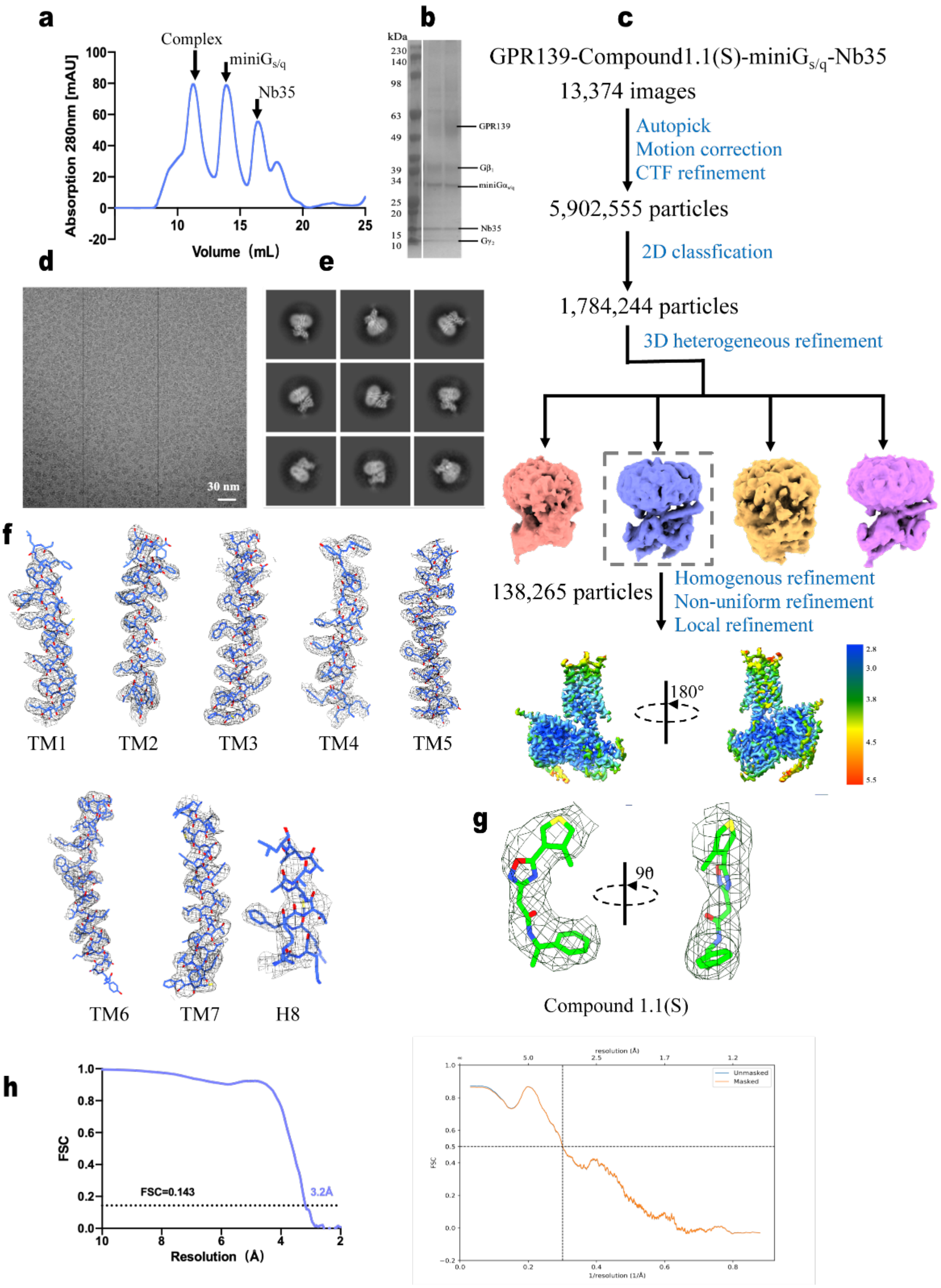

Supplementary Figure 8. Sample preparation and cryo-EM data processing of the GPR139–compound **1.1**–miniG$_{s/q}$–Nb35 complex. (a, b) Superdex 200 size-exclusion chromatography and SDS-PAGE analysis of the purified GPR139–compound **1.1(S)**–miniG$_{s/q}$–Nb35 complex. (**c**) Workflow of the cryo-EM data processing for the GPR139–compound **1.1(S)**–miniG$_{s/q}$–Nb35 complex. (d,e) Representative cryo-EM micrographs from 13,374 movies (**c**) (scale bar, 30 nm) and 2D classification of the complex. (f, g) Cryo-EM density map of each transmembrane helix and compound **1.1(S)**. (h) Final local resolution estimation of the cryo-EM density map and the gold-standard Fourier shell correlation (FSC) curve. The model versus map FSC is also shown, with the FSC = 0.5 threshold indicated.

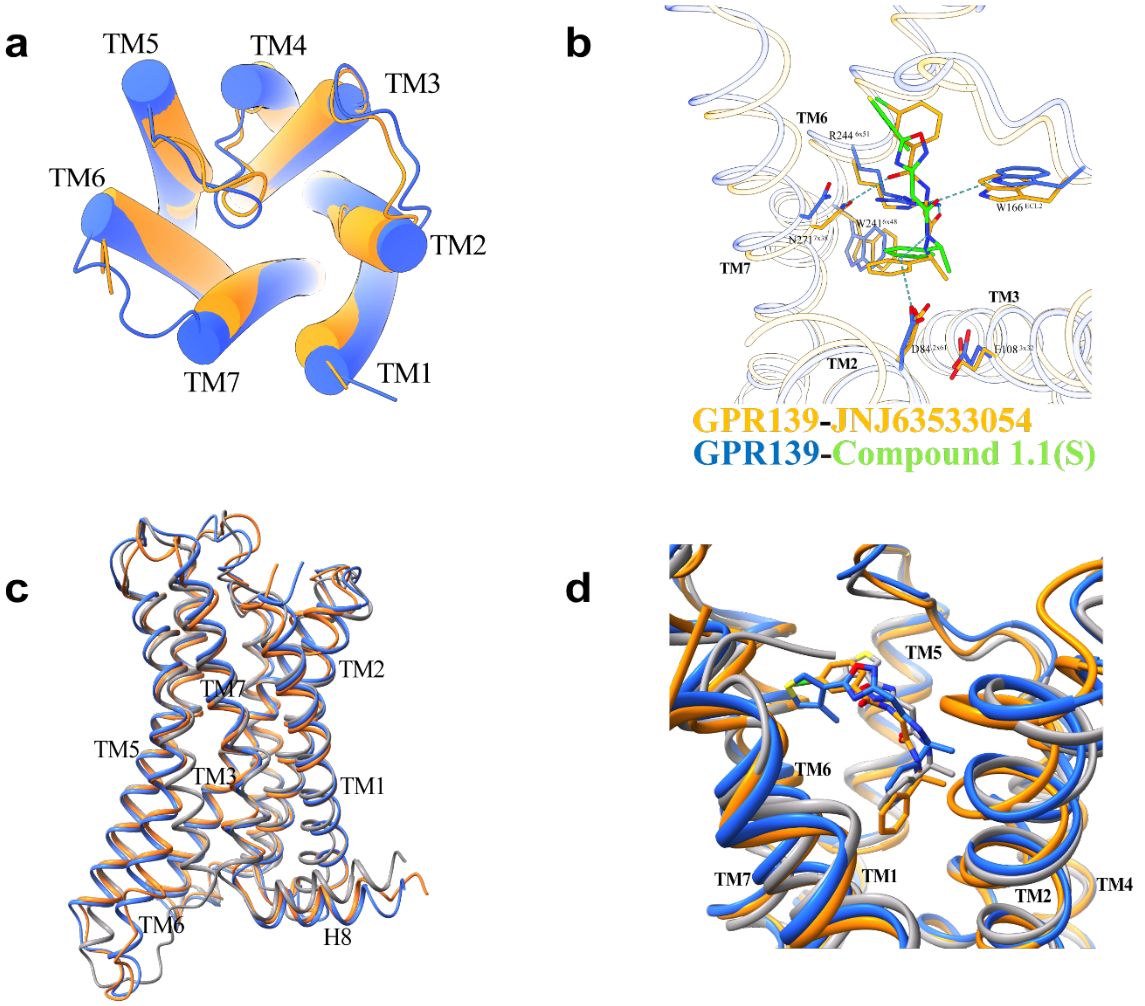

Supplementary Figure 9. Structural comparison of GPR139 bound to compound **1.1(S)** and **JNJ-63533054.** (a-b) Superimposition of GPR139 in complex with compound **1.1(S)** (blue) and **JNJ-63533054** (orange). (a) Extracellular view of the superimposed structures, highlighting the relative displacement of the transmembrane regions between the two ligand-bound conformations. The transmembrane helices are shown as cylinders. (b) Ligand-induced conformational changes in the extracellular regions of the binding pocket. (c) Structural superposition of GPR139 bound to compound **1.1(S)**, GPR139 bound to **JNJ-63533054**, and the AlphaFold3-predicted (grey) GPR139 structure. (d) Close-up view of the orthosteric binding pocket from the three superimposed structures. In (b-d), the receptor and ligands are shown as ribbons and sticks, respectively.

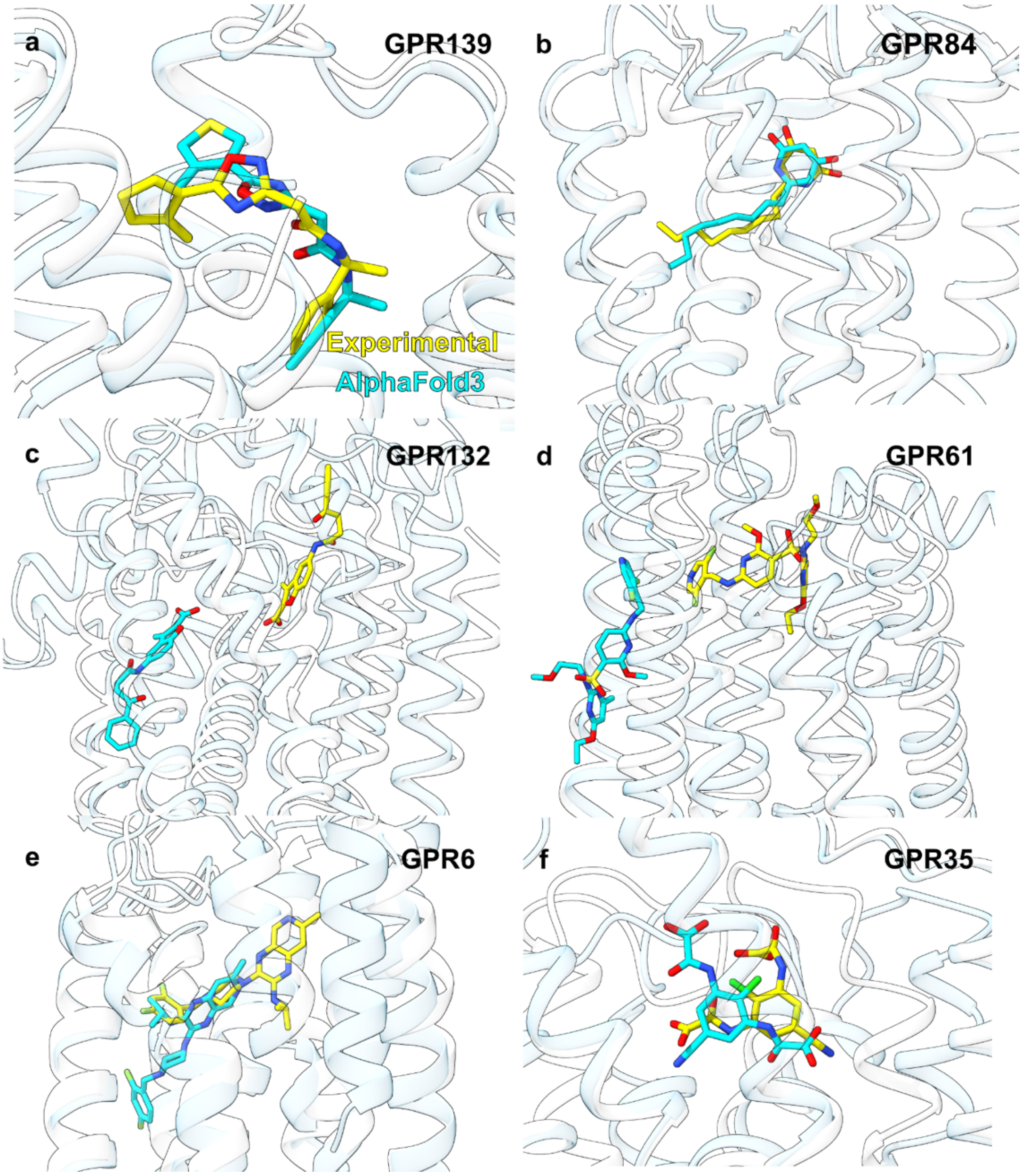

Supplementary Figure 10. AlphaFold3-predicted protein-ligand complex structures for five orphan GPCRs and GPR139 with small molecule ligands. Excluding GPR139, these orphan GPCRs lack experimental structures in the AlphaFold3 training set. Panels include: (a) GPR139, (b) GPR84, (c) GPR132, (d) GPR61 (e) GPR6 and (f) GPR35. Receptors are represented as blue (AlphaFold3 model) and white (experimental structure) cartoons. Ligands are depicted in yellow (experimental structures) and green (AlphaFold3 predictions) sticks. The PDB accession codes for the experimental structures are as follows: 9M42 (GPR139), 8T1V (GPR6), 8HVI (GPR132), 8TB7 (GPR61), 8J19 (GPR84) and 8H8J (GPR35).[1–5]

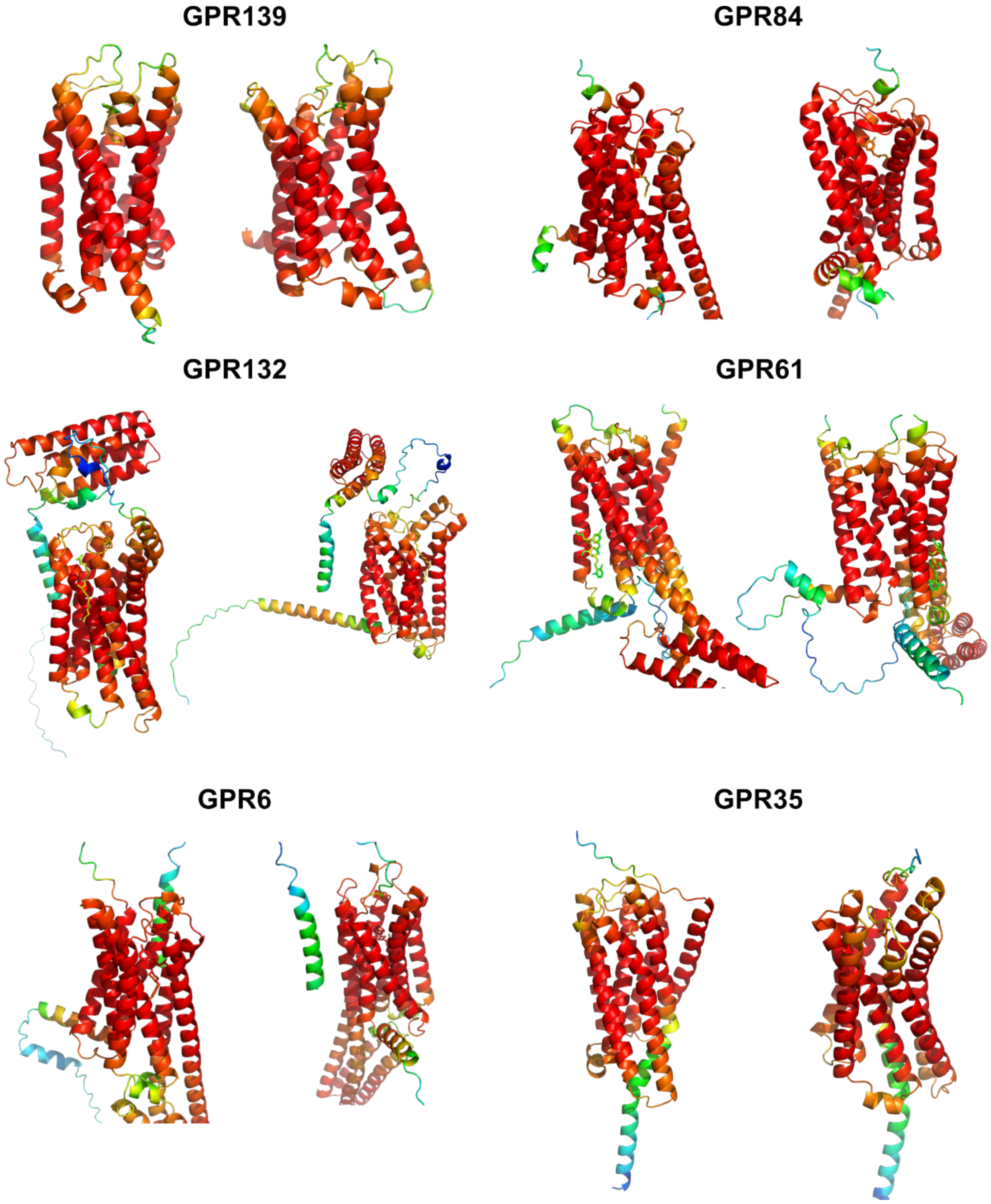

Supplementary Figure 11. pLDDT scores of AlphaFold3-predicted protein–ligand complex structures for five orphan GPCRs and GPR139 bound to small-molecule ligands. Red indicates regions of high confidence, while green and blue represent regions of low confidence.

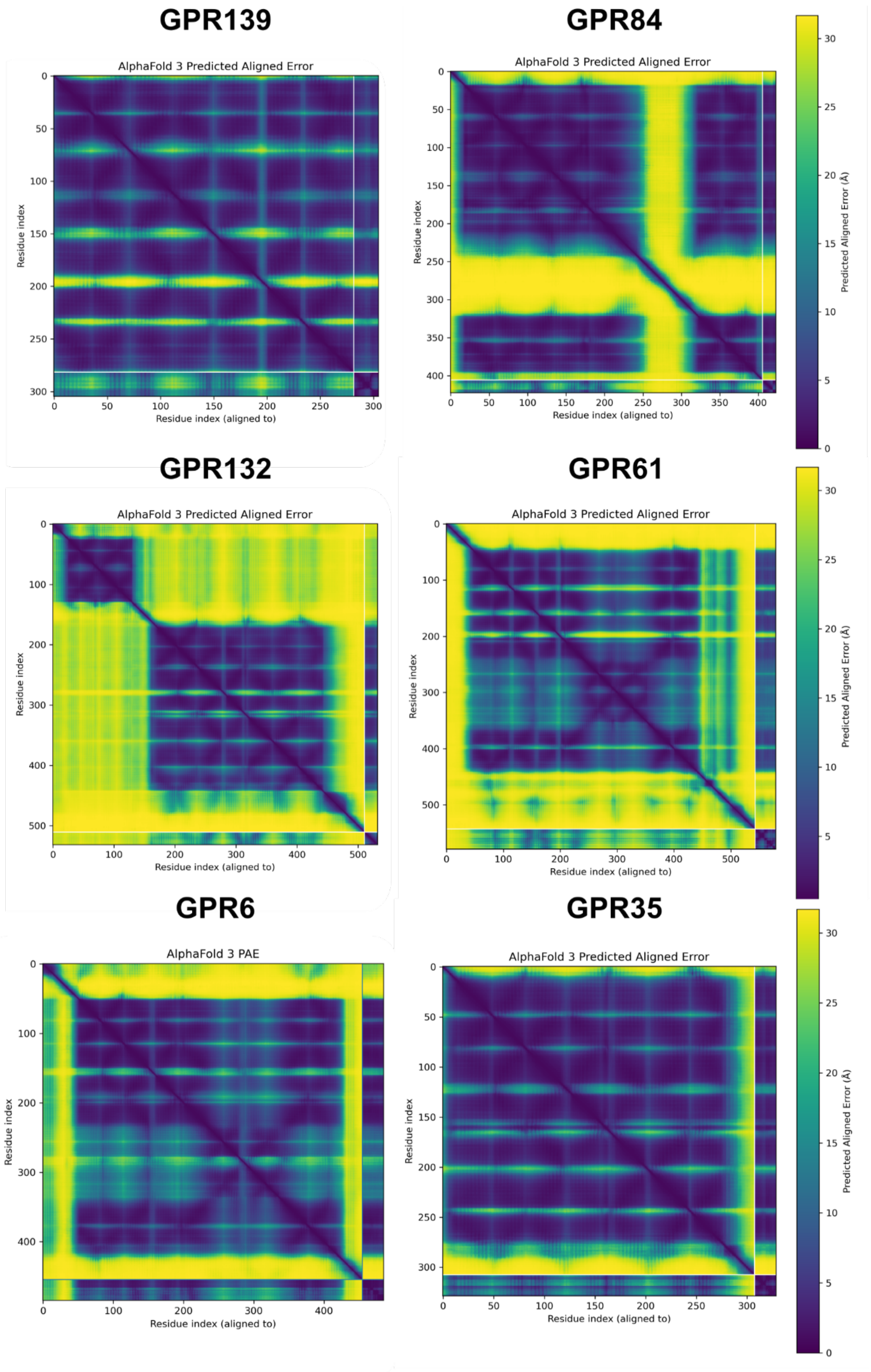

Supplementary Figure 12. Predicted Aligned Error (PAE) of AlphaFold3-predicted protein–ligand complex structures for five orphan GPCRs and GPR139 bound to small-molecule ligands.

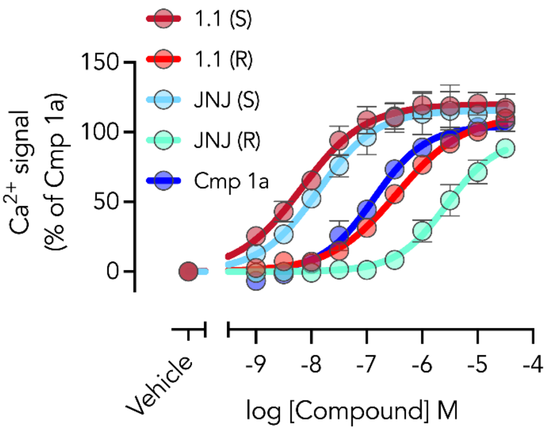

Supplementary Figure 13. Evaluation of preferred configuration of compound **1.1.** Compound **1.1** was synthesized as pure S- and R-enantiomer, **1.1(S)** and **1.1(R)**, and the preferred configuration was assessed by measuring Ca^{2+} mobilization in a stable GPR139-CHOk1 cell line. **JNJ(S)** and **JNJ(R)** are the enantiomers of **JNJ-63533054**. Data represent mean ± SEM of at least three independent experiments performed in triplicates and are normalized to buffer (0%) and 10 µM **Lundbeck Cmp 1a** (100%).

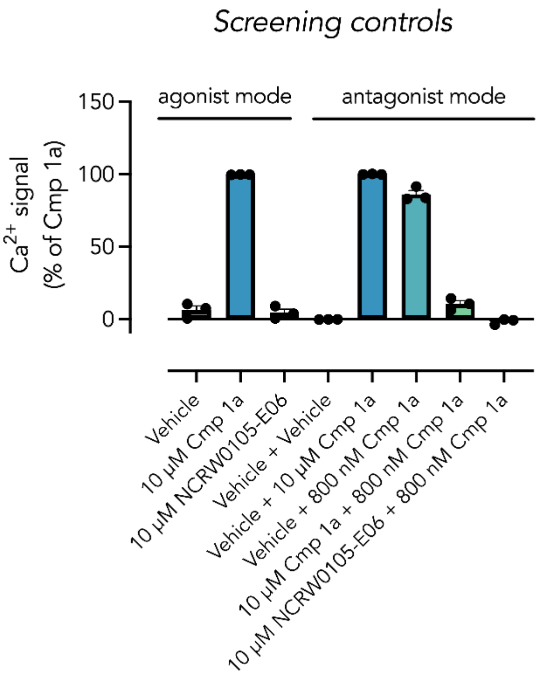

Supplementary Figure 14. Overview and controls of the two-step Ca^{2+} mobilization screening protocol for simultaneous identification of agonist and antagonists. **Cmp 1a** is a reference agonist from H. Lundbeck A/S. **NCRW0105-E06** is a reference antagonist. Ca^{2+} mobilization was measured in a stable GPR139-CHOk1 cell line. In the first step, the screening compounds are applied (10 µM) and Ca^{2+} mobilization is measured immediately to test for agonistic activity (agonist mode). Following 20 minutes incubation with the screening compounds, in the second step an EC_{80} concentration of **Lundbeck Cmp 1a** (800 nM) is applied to test for antagonistic activity (antagonist mode). Agonists show a response in the first step and diminish subsequent response of the reference agonist in the second step due to desensitization. Antagonists do not show a response in the first step and decrease the response of the reference agonist in the second step. Data represent mean ± SEM of three independent experiments performed in triplicates and are normalized to buffer (0%) and 10 µM **Lundbeck Cmp 1a** (100%).

Supplementary chemistry methods

1. Synthesis of compounds 1-5

General considerations: All chemicals for the synthesis of the studied compounds were provided by Enamine Ltd. (www.enamine.net). All solvents were treated according to standard methods. ^{1}H NMR spectra were recorded at 500 MHz, ^{13}C NMR spectra at 101 and 151 MHz (Varian or Bruker spectrometers). ^{1}H chemical shifts are calibrated using residual nondeuterated solvent DMSO, δ = 2.50 ppm. ^{13}C chemical shifts are calibrated using residual solvent DMSO, δ = 39.5 ppm. Coupling constants are given in Hz. LCMS analysis was performed utilizing an Agilent 1200 Series LCMSD system with DAD/ELSD (column Zorbax SB-C18 1.8 µm 4.6x15 mm; solvent A (water, 0.1% formic acid) and solvent B (acetonitrile, 0.1% formic acid); gradient 0% – 100% solvent B, run time, 1.8 min; flow rate, 3 mL/min) and Agilent LCMSD SL (G6130A) or SL (G6140A) mass spectrometer (APCI mode). All the LCMS data were obtained using positive/negative mode switching.

Synthetic procedures:

Method 1. The synthesis was performed according to the previously published procedure[7].

Method 2. The synthesis was performed according to the previously published procedure[8].

Method 3. A carboxylic acid (100 mg) was dissolved in 0.5 mL of 10% hydroxybenzotriazole (HOBt) in DMF followed by the addition of an amine (1 mol equiv to the carboxylic acid) and 1-ethyl-3-(3-dimethylaminopropyl)carbodiimide (EDC, 1.2 mol equiv to the carboxylic acid). The resulting mixture was shaken for 24 h at room temperature. Then, $CHCl_3$ (2 mL) was added, and the organic phase was washed with water, dried over sodium sulfate, and evaporated under reduced pressure. The crude was dissolved in 0.5 mL of DMSO and further purified by preparative HPLC.

Spectral description of the studied compounds:

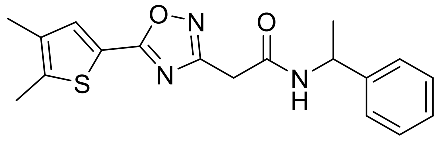

2-[5-(4,5-dimethylthiophen-2-yl)-1,2,4-oxadiazol-3-yl]-N-(1-phenylethyl)acetamide – Compound 1 (Method 1).
Yield: 9%; purity, >95% (assessed by LCMS).
^{1}H NMR (500 MHz, DMSO-d_6) δ 8.72 (d, J = 7.9 Hz, 1H), 7.71 (s, 1H), 7.36 – 7.27 (m, 4H), 7.25 – 7.18 (m, 1H), 4.90 (p, J = 7.1 Hz, 1H), 3.70 – 3.66 (m, 2H), 2.40 (s, 3H), 2.15 (s, 3H), 1.36 (d, J = 7.0 Hz, 3H).
^{13}C NMR (101 MHz, DMSO-d_6) δ 170.9, 166.8, 165.6, 165.6, 144.7, 142.2, 136.2, 135.4, 128.7, 127.1, 126.4, 119.8, 48.7, 48.6, 33.4, 22.9, 13.7.
LCMS (APCI) m/z [M+H] calculated for C18H20N3O2S: 342.1; found: 342.0.

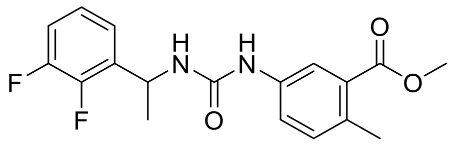

methyl 5-(3-(1-(2,3-difluorophenyl)ethyl)ureido)-2-methylbenzoate – Compound 2 (Method 2).
Yield: 53%; purity, >95% (assessed by LCMS).
^{1}H NMR (500 MHz, DMSO-d_6) δ 8.56 (s, 1H), 7.93 (d, *J* = 2.5 Hz, 1H), 7.35 (dd, *J* = 8.3, 2.5 Hz, 1H), 7.28 (dtd, *J* = 10.0, 7.6, 2.3 Hz, 1H), 7.19 (h, *J* = 5.3 Hz, 2H), 7.14 (t, *J* = 7.5 Hz, 1H), 6.75 (d, *J* = 7.6 Hz, 1H), 5.04 (p, *J* = 7.1 Hz, 1H), 3.77 (s, 3H), 2.38 (s, 3H), 1.38 (d, *J* = 7.0 Hz, 3H).
^{13}C NMR (101 MHz, DMSO-d_6) δ 167.7, 154.6, 148.8, 138.5, 135.3, 132.3, 129.7, 125.2, 122.8, 121.8, 119.5, 116.1, 52.2, 43.8, 22.1, 20.8.
LCMS (APCI) m/z [M+H] calculated for C18H19F2N2O3: 349.1; found: 349.2.

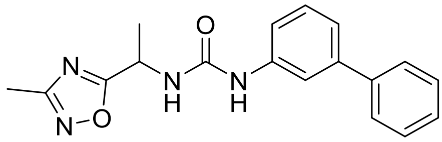

1-([1,1'-biphenyl]-3-yl)-3-(1-(3-methyl-1,2,4-oxadiazol-5-yl)ethyl)urea – Compound 3 (Method 2).
Yield: 32%; purity, >95% (assessed by LCMS).
^{1}H NMR (500 MHz, DMSO-d_6) δ 8.73 (s, 1H), 7.72 (d, *J* = 2.2 Hz, 1H), 7.60 – 7.54 (m, 2H), 7.44 (t, *J* = 7.6 Hz, 2H), 7.37 – 7.26 (m, 3H), 7.19 (dt, *J* = 6.4, 2.1 Hz, 1H), 6.96 (d, *J* = 7.5 Hz, 1H), 5.07 (p, *J* = 7.1 Hz, 1H), 2.31 (s, 3H), 1.50 (d, *J* = 7.1 Hz, 3H).
^{13}C NMR (151 MHz, DMSO-d_6) δ 181.1, 167.3, 154.9, 141.3, 140.9, 140.8, 129.8, 129.4, 127.9, 127.1, 120.5, 117.5, 116.6, 43.2, 19.5, 11.6.
LCMS (APCI) m/z [M+H] calculated for C18H19N4O2: 323.1; found: 323.2.

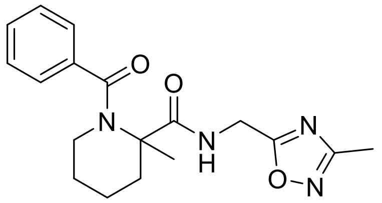

1-benzoyl-2-methyl-N-((3-methyl-1,2,4-oxadiazol-5-yl)methyl)piperidine-2-carboxamide – Compound 4 (Method 3).
Yield: 64%; purity, >95% (assessed by LCMS).
^{1}H NMR (500 MHz, DMSO-d_6) δ 8.30 (t, *J* = 5.8 Hz, 1H), 7.50 – 7.38 (m, 4H), 4.52 (dd, *J* = 16.7, 5.9 Hz, 1H), 4.35 (dd, *J* = 16.7, 5.5 Hz, 1H), 3.46 (dt, *J* = 13.9, 4.8 Hz, 1H), 3.29 (s, 1H), 3.06 (ddd, *J* = 13.6, 9.5, 3.5 Hz, 1H), 2.28 (s, 3H), 1.97 – 1.88 (m, 1H), 1.73 – 1.35 (m, 6H).
^{13}C NMR (126 MHz, DMSO-d_6) δ 177.7, 174.8, 172.2, 167.2, 137.6, 130.1, 128.7, 127.6, 61.4, 45.1, 36.1, 35.1, 23.5, 18.9, 18.4, 11.6.
LCMS (APCI) m/z [M-H] calculated for C18H21N4O3: 341.2; found: 341.1.

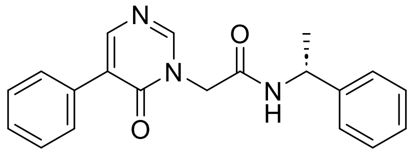

(R)-2-(6-oxo-5-phenylpyrimidin-1(6H)-yl)-N-(1-phenylethyl)acetamide – Compound 5(R) (Method 3).

Yield: 21%; purity, >95% (assessed by LC/MS).

^{1}H NMR (500 MHz, DMSO-d_6) δ 8.79 (d, J = 7.9 Hz, 1H), 8.38 (s, 1H), 8.13 (s, 1H), 7.68 – 7.63 (m, 2H), 7.40 (td, J = 7.3, 1.6 Hz, 2H), 7.37 – 7.31 (m, 1H), 7.32 (s, 2H), 7.33 – 7.27 (m, 2H), 7.21 (tt, J = 5.5, 2.4 Hz, 1H), 4.92 (p, J = 7.1 Hz, 1H), 4.68 (s, 2H), 1.37 (d, J = 7.0 Hz, 3H).

^{13}C NMR (151 MHz, DMSO-d_6) δ 165.7, 159.9, 152.9, 151.4, 144.6, 133.7, 128.7, 128.7, 128.6, 128.5, 127.2, 126.4, 126.1, 49.0, 48.7, 23.0.

LCMS (APCI) m/z [M-H] calculated for $C_{20}H_{20}N_3O_2$: 334.2; found: 334.2.

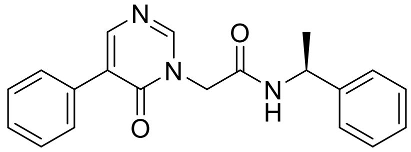

(S)-2-(6-oxo-5-phenylpyrimidin-1(6H)-yl)-N-(1-phenylethyl)acetamide – **Compound 5(S) (Method 3).**

Yield: 25%; purity, >95% (assessed by LCMS).

^{1}H NMR (500 MHz, DMSO-d6) δ 8.79 (d, J = 7.8 Hz, 1H), 8.38 (s, 1H), 8.13 (s, 1H), 7.66 (dd, J = 8.5, 1.4 Hz, 2H), 7.40 (dd, J = 8.3, 6.7 Hz, 2H), 7.37 – 7.32 (m, 1H), 7.35 – 7.27 (m, 4H), 7.22 (tt, J = 5.4, 2.3 Hz, 1H), 4.92 (p, J = 7.0 Hz, 1H), 4.68 (s, 2H), 1.37 (d, J = 7.0 Hz, 3H).

^{13}C NMR (126 MHz, DMSO-d_6) δ 165.8, 159.9, 153.0, 151.4, 144.6, 133.8, 128.8, 128.6, 127.2, 126.5, 126.2, 49.0, 48.8, 23.0.

LCMS (APCI) m/z [M-H] calculated for $C_{20}H_{20}N_3O_2$: 334.2; found: 334.0.

2. General synthetic procedures for compounds JNJ, 1.1, and 1.5. All reagents were purchased from Fluorochem, Sigma-Aldrich, Enamine and Chemtronica. DCM, methanol, DMF, and acetonitrile (99.9%) were purchased from VWR International AB, whereas THF was purchased from Sigma-Aldrich. Reagents and solvents were used as such without further purification. All reactions involving air or moisture-sensitive reagents or intermediates were performed under a nitrogen atmosphere. Mainly LC-MS was used for monitoring reactions using an Agilent 1260 Infinity II series HPLC having an Agilent C18 Porosell 120 column (2.1 × 50 mm, 2.7 μm). Acetonitrile–water (both + 0.1% formic acid, gradient of 5-95% of acetonitrile over 6 min with flow rate of 0.5 mL/min) was used as mobile phase and a LCMSD iQ (G616A) mass spectrometer operating in electrospray ionization mode was used for detection of molecular ions. Silica gel 60 F_{254} TLC plates from Merck were sometimes used for monitoring reactions and particularly during purification of compounds. Visualization of the developed TLC was done using UV light (254 nm) and staining with ninhydrin or anisaldehyde. After workup, organic phases were dried over Na_2SO_4/$MgSO_4$ and filtered before being concentrated under reduced pressure. Silica gel (Matrex, 60 Å, 35–70 μm, Grace Amicon) was used for purification of intermediate compounds with flash column chromatography. ^1H and ^{13}C NMR spectra for synthesized compounds were recorded at 298 K on an Agilent Technologies 400 MR spectrometer at 400 MHz or 100 MHz, respectively, or on a Bruker Avance Neo spectrometer at 500 MHz or 125 MHz, respectively. Chemical shifts are reported in parts per million (ppm, δ) referenced to the residual ^1H and ^{13}C resonance of the solvent [CD_3OD, δ_H 3.31, δ_C 49.0; DMSO-d_6, δ 2.50, δ_C 39.5]. Splitting patterns are designated as follows: s (singlet), d (doublet), t (triplet), m (multiplet), and br (broad). Coupling constants (J values) are listed in hertz (Hz). Preparative reversed-phase HPLC was performed on a Kromasil C8 column (250 × 21.2 mm, 5 μm) on a Gilson HPLC equipped with Gilson 322 pump, UV/Visible-156 detector and 202 fraction collector using acetonitrile-water gradients as eluents with a flow rate of 15 mL/min and detection at 210 or 254 nm. Unless otherwise stated, all the tested compounds were purified by HPLC. The purity of the tested compounds is ≥95% as determined by HPLC and high resolution ^1H NMR spectroscopy (500 MHz).

3. Synthesis of JNJ(S) and JNJ(R). The synthesis of compounds **JNJ-63533054** (**JNJ(S)**) and its enantiomer **JNJ(R)**, was performed using modified versions of the procedures described in Dvorak et al 2015.[6]

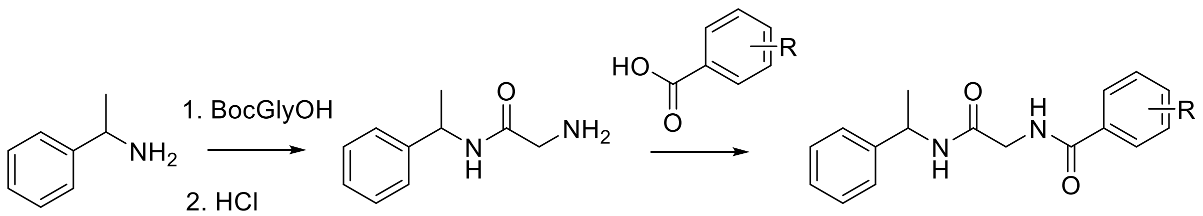

Briefly, *R*- or *S*-1-phenylethan-1-amine was coupled with BocGlyOH followed by Boc deprotection using HCl to afford an intermediate, primary amine. This was submitted to a 2nd amide coupling with the corresponding benzoic acid to afford target compounds as white solids after purification by HPLC.

General one pot procedure. A mixture of *R*- or *S*-1-phenylethan-1-amine (0.05 mmol, 1 equiv), BocGlyOH (0.05 mmol, 1 equiv), TBTU (0.05 mmol, 1 equiv), and Et_3N (0.05 mmol, 1 equiv) in DCM (0.5 mL) was stirred at rt until full conversion was obtained (ca. 30 min). The solvent was removed by evaporation, then 4N HCl in dioxane (0.2 mL) was added while stirring at rt for 30 min for Boc deprotection. The solvent was again removed, followed by addition of ArCOOH (0.05 mmol, 1 equiv), TBTU (0.05 mmol, 1 equiv), Et_3N (0.30 mmol, 6 equiv), and DCM (1 mL). The reaction was stirred at rt for 30 min and formic acid (50 µL) was added. After removal of the solvent, the residue was dissolved in DMSO (1 mL), filtered and purified by HPLC using a gradient of 20-70% acetonitrile in H_2O (H_2O + 0.1% formic acid) to afford the desired product as a white solid. Yields of **JNJ(S)** and **JNJ(R)**: 44-57%.

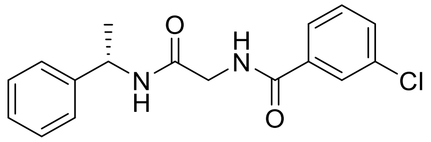

JNJ(S), (S)-3-chloro-N-(2-oxo-2-((1-phenylethyl)amino)ethyl)benzamide.
^{1}H NMR (500 MHz, CD_3OD) δ 7.89 (t, *J* = 1.9 Hz, 1H), 7.79 (d, *J* = 7.6 Hz, 1H), 7.58 – 7.52 (m, 1H), 7.45 (t, *J* = 7.9 Hz, 1H), 7.40 – 7.28 (m, 4H), 7.25 – 7.18 (m, 1H), 5.05 (q, *J* = 7.0 Hz, 1H), 4.08 (d, *J* = 16.4 Hz, 1H), 4.02 (d, *J* = 16.4 Hz, 1H), 1.47 (d, *J* = 7.0 Hz, 3H).
^{13}C NMR (126 MHz, CD_3OD) δ 170.5, 168.9, 145.0, 137.1, 135.6, 132.7, 131.2, 129.5, 128.7, 128.1, 127.1, 126.8, 50.2, 44.0, 22.4.
LCMS (ESI+): calculated for $C_{17}H_{18}ClN_2O_2$ $(M+H)^+$: 317.1; found 317.3.

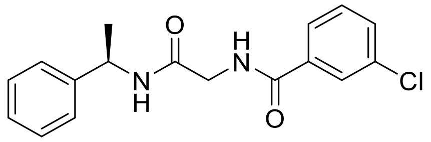

JNJ (R), (R)-3-chloro-N-(2-oxo-2-((1-phenylethyl)amino)ethyl)benzamide.
^{1}H NMR (500 MHz, CD_3OD) δ 7.89 (t, *J* = 1.9 Hz, 1H), 7.79 (d, *J* = 7.6 Hz, 1H), 7.58 – 7.52 (m, 1H), 7.45 (t, *J* = 7.9 Hz, 1H), 7.40 – 7.28 (m, 4H), 7.25 – 7.18 (m, 1H), 5.05 (q, *J* = 7.0 Hz, 1H), 4.08 (d, *J* = 16.4 Hz, 1H), 4.02 (d, *J* = 16.4 Hz, 1H), 1.47 (d, *J* = 7.0 Hz, 3H).
^{13}C NMR (126 MHz, CD_3OD) δ 170.5, 168.9, 145.0, 137.1, 135.6, 132.7, 131.2, 129.5, 128.7, 128.1, 127.1, 126.8, 50.2, 44.0, 22.4.
LCMS (ESI+): calculated for $C_{17}H_{18}ClN_2O_2$ $(M+H)^+$: 317.1; found 317.3.

4. Synthesis of compounds 1.1(S), 1.1(R), 1.5(S), and 1.5(R)

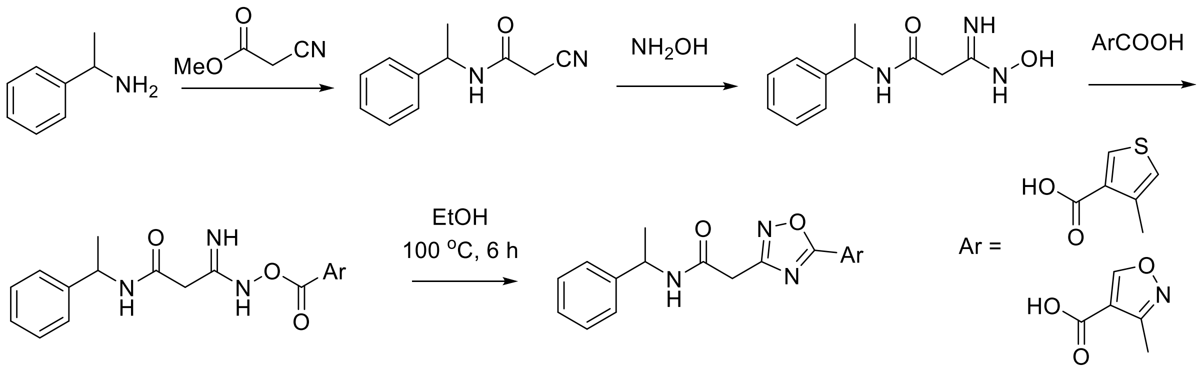

Briefly, aminolysis of methyl 2-cyanoacetate by *R*- or *S*-1-phenylethan-1-amine afforded a cyano derivative which was reacted with hydroxylamine, followed by ester formation to afford a hydrazino ester intermediate. Heating the latter in EtOH at 100 °C for 6 h afforded the target compounds as white solids after purification by HPLC.

General one pot procedure: A solution of *R*- or *S*-1-phenylethan-1-amine (0.2 mmol, 1 equiv) and methyl 2-cyanoacetate (0.2 mmol, 1 equiv) in MeOH (0.2 mL) was stirred overnight at rt. Then $NH_2OH.HCl$ (0.2 mmol, 1 equiv) and Et_3N (0.2 mmol, 1 equiv) were added and the reaction solution was stirred at 60 °C for 30 min. The solvent was removed and 2-methylthiophene-3-carboxylic acid or 3-methylisoxazole-4-carboxylic acid (0.2 mmol, 1 equiv), DCC (0.2 mmol, 1 equiv) and DCM (0.5 mL) were added after which the solution was stirred at rt for 30 min. DCM was removed and EtOH (0.5 mL) was added. Then the mixture was heated at 100 °C in a sealed vial for 6 h. After removal of the solvent, the residue was diluted with DMSO (1 mL), filtered and purified by HPLC using a gradient of 20-80% acetonitrile in H_2O (H_2O + 0.1% formic acid) to afford the desired product as a white solid. Yields of **1.1(S)** and **1.1(R)**: 15%.

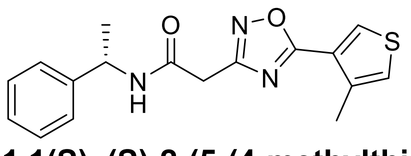

1.1(S), (S)-2-(5-(4-methylthiophen-3-yl)-1,2,4-oxadiazol-3-yl)-N-(1-phenylethyl)acetamide.

^{1}H NMR (500 MHz, CD_3OD) δ 8.32 (d, *J* = 3.3 Hz, 1H), 7.36 (d, *J* = 7.3 Hz, 2H), 7.31 (t, *J* = 7.7 Hz, 2H), 7.28 – 7.19 (m, 2H), 5.05 (q, *J* = 7.0 Hz, 1H), 3.78 (s, 2H), 2.53 (d, *J* = 1.1 Hz, 3H), 1.49 (d, *J* = 7.0 Hz, 3H).

^{13}C NMR (126 MHz, CD_3OD) δ 173.9, 168.5, 167.2, 144.8, 138.6, 133.4, 129.5, 128.1, 127.1, 126.2, 125.2, 50.5, 34.3, 22.4, 16.2.

LCMS (ESI+): calculated for $C_{17}H_{18}N_3O_2S$ $(M+H)^+$: 328.1; found 328.3.

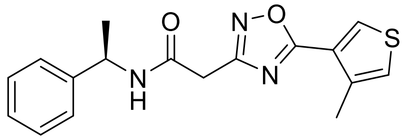

1.1(R), (R)-2-(5-(4-methylthiophen-3-yl)-1,2,4-oxadiazol-3-yl)-N-(1-phenylethyl)acetamide.

^{1}H NMR (500 MHz, CD_3OD) δ 8.32 (d, *J* = 3.3 Hz, 1H), 7.36 (d, *J* = 7.3 Hz, 2H), 7.31 (t, *J* = 7.7 Hz, 2H), 7.28 – 7.19 (m, 2H), 5.05 (q, *J* = 7.0 Hz, 1H), 3.78 (s, 2H), 2.53 (d, *J* = 1.1 Hz, 3H), 1.49 (d, *J* = 7.0 Hz, 3H).

^{13}C NMR (126 MHz, CD_3OD) δ 173.9, 168.5, 167.2, 144.8, 138.6, 133.4, 129.5, 128.1, 127.1, 126.2, 125.2, 50.5, 34.3, 22.4, 16.2.

LCMS (ESI+): calculated for $C_{17}H_{18}N_3O_2S$ $(M+H)^+$: 328.1; found 328.3.

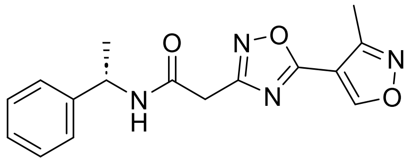

1.5(S)-2-(5-(3-methylisoxazol-4-yl)-1,2,4-oxadiazol-3-yl)-N-(1-phenylethyl)acetamide

^{1}H NMR (500 MHz, CD_3OD) δ 9.47 (s, 1H), 7.39 – 7.28 (m, 4H), 7.27 – 7.20 (m, 1H), 5.04 (q, *J* = 7.0 Hz, 1H), 3.80 (s, 2H), 2.57 (s, 3H), 1.49 (d, *J* = 7.0 Hz, 3H).

^{13}C NMR (126 MHz, CD_3OD) δ 170.4, 168.3, 167.4, 163.4, 158.9, 144.8, 129.5, 128.2, 127.1, 108.5, 50.6, 34.1, 22.4, 11.0.

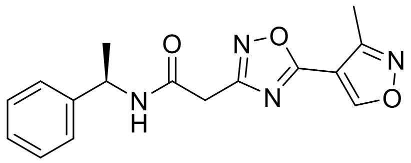

1.5(R)-2-(5-(3-methylisoxazol-4-yl)-1,2,4-oxadiazol-3-yl)-N-(1-phenylethyl)acetamide

^{1}H NMR (500 MHz, CD_3OD) δ 9.47 (s, 1H), 7.39 – 7.28 (m, 4H), 7.27 – 7.20 (m, 1H), 5.04 (q, *J* = 7.0 Hz, 1H), 3.80 (s, 2H), 2.57 (s, 3H), 1.49 (d, *J* = 7.0 Hz, 3H).

^{13}C NMR (126 MHz, CD_3OD) δ 170.4, 168.3, 167.4, 163.4, 158.8, 144.8, 129.5, 128.2, 127.1, 108.5, 50.6, 34.1, 22.4, 11.0.

5. HPLC chromatograms

LCMS of compound **1**

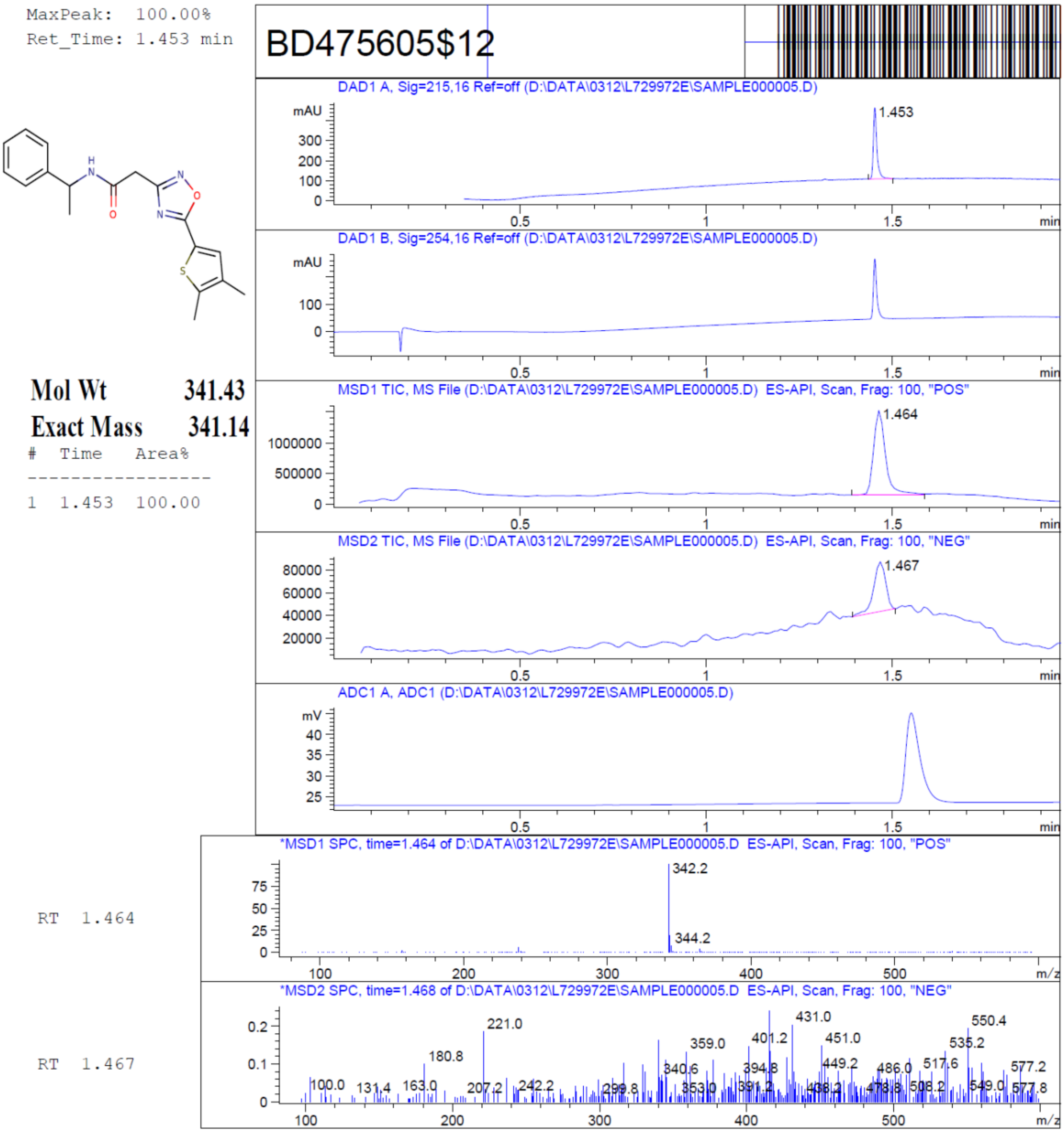

LCMS of compound **2**

MaxPeak: 100.00%
Ret_Time: 1.360 min

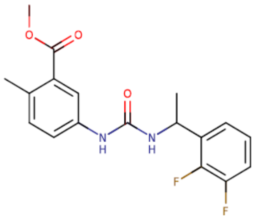

Mol Wt 348.34
Exact Mass 348.15

#	Time	Area%
1	1.360	100.00

V880944$3

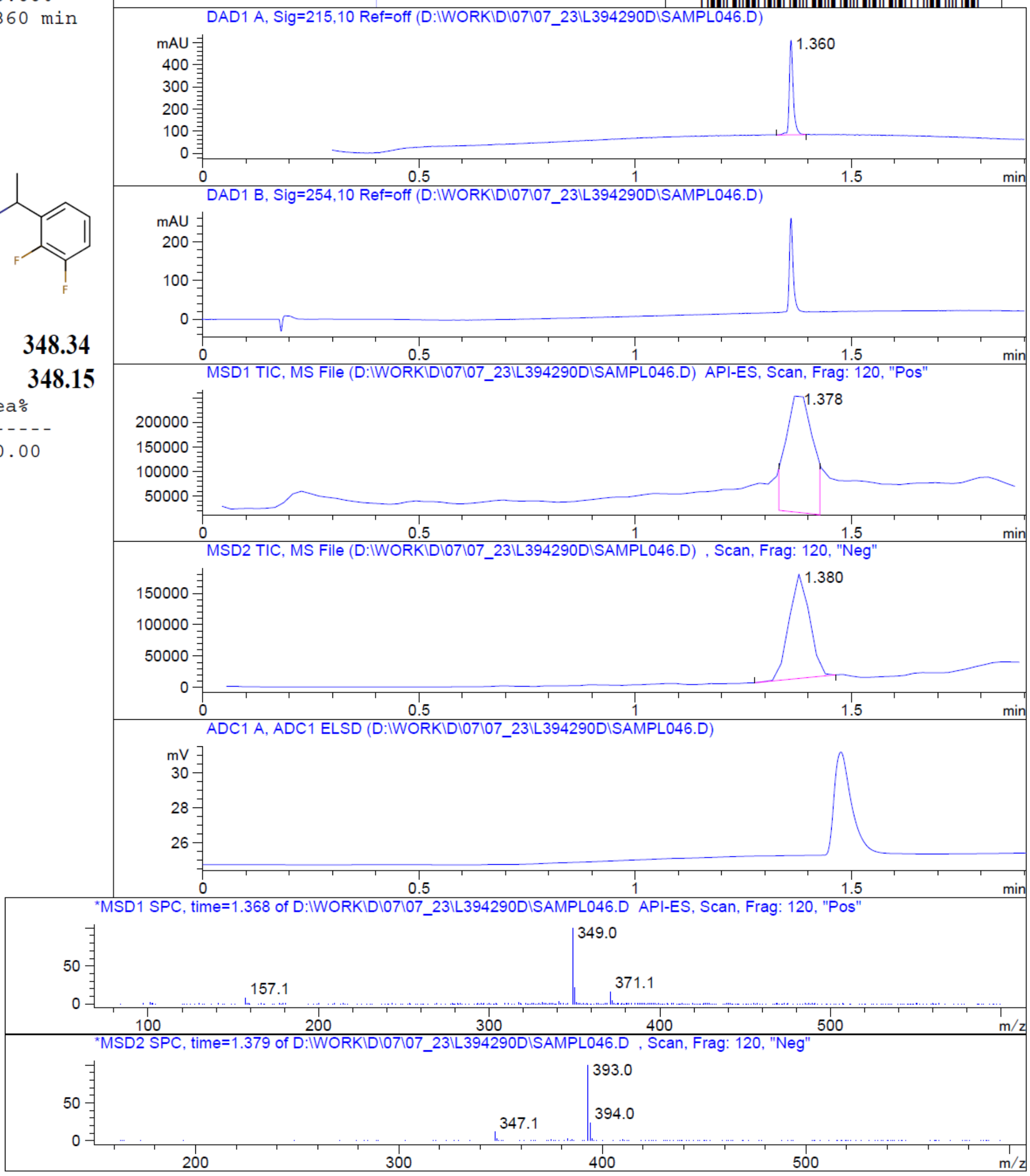

Inj.Date 7/23/2021 K P2-F-06 -VL- Acq. Method C:\HPCHEM\-> ->

LCMS of compound **3**

MaxPeak: 99.40%
Ret_Time: 1.071 min

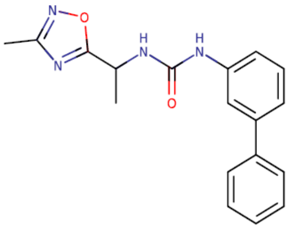

Mol Wt 322.36

Exact Mass 322.16

#	Time	Area%
1	1.071	99.40
2	1.106	0.60

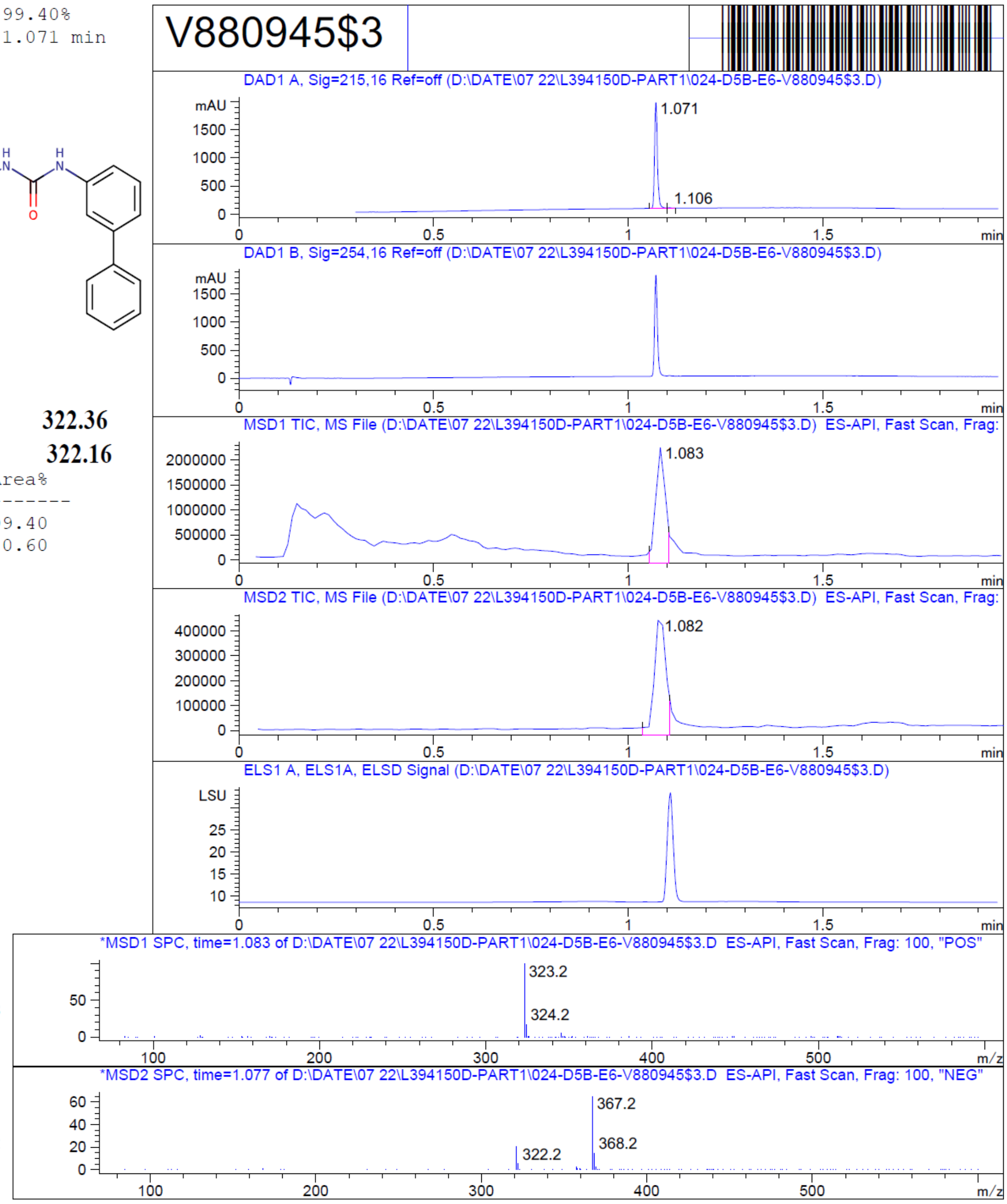

RT 1.083

RT 1.082

Inj.Date 7/22/2021 E Acq. Method C:\Users\ -> ->

LCMS of compound **4**

MaxPeak: 100.00%
Ret_Time: 1.164 min

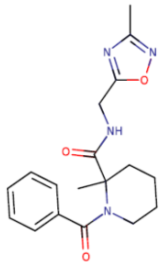

Mol Wt 342.39
Exact Mass 342.19

#	Time	Area%
1	1.164	100.00

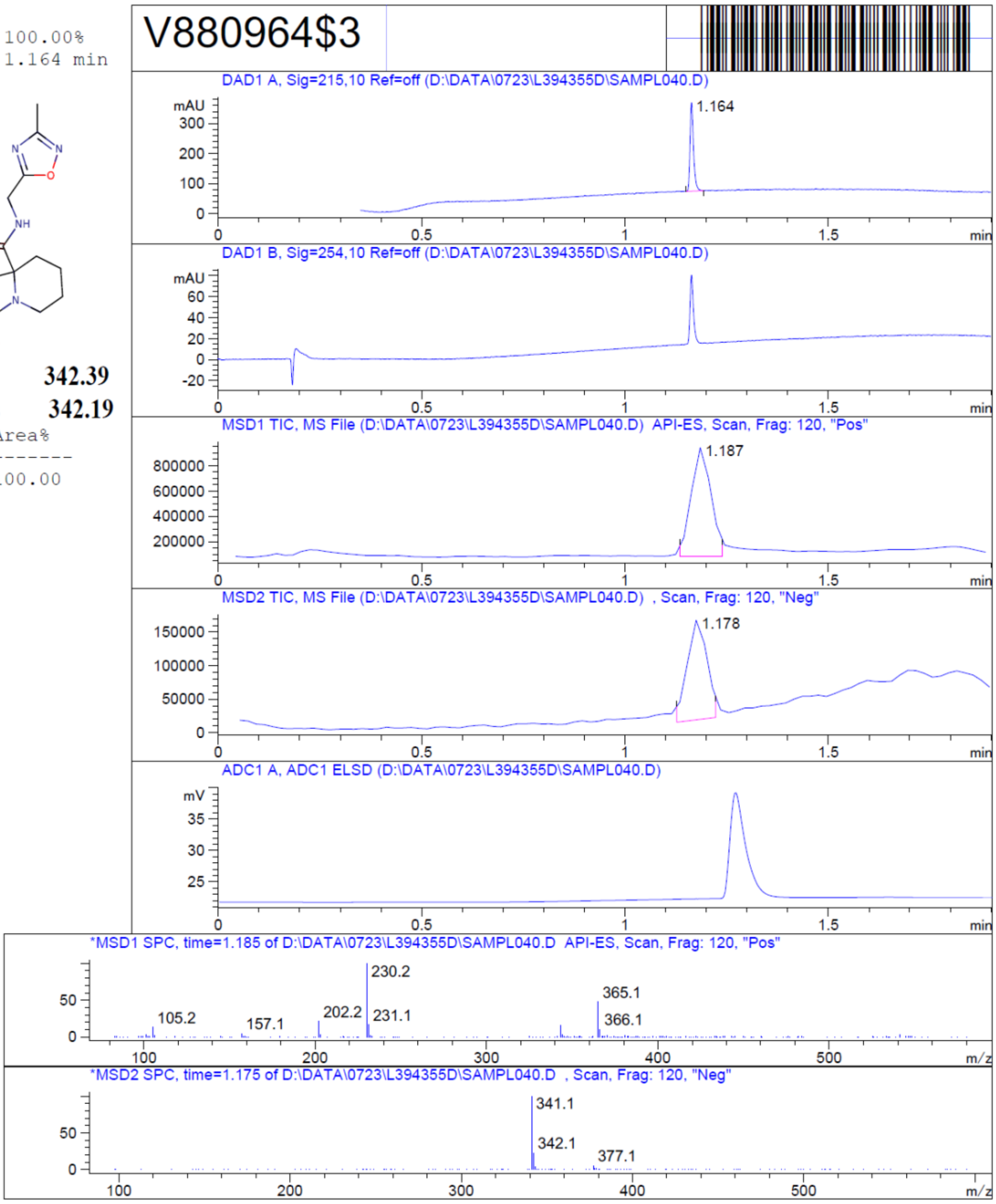

RT 1.187

RT 1.178

Inj.Date 7/23/2021 H -SL- Acq. Method C:\HPCHEM\-> ->

LCMS of compound **5(R)**

MaxPeak: 100.00%
Ret_Time: 1.039 min

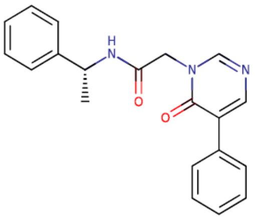

Mol Wt 333.38
Exact Mass 333.17

#	Time	Area%
1	1.039	100.00

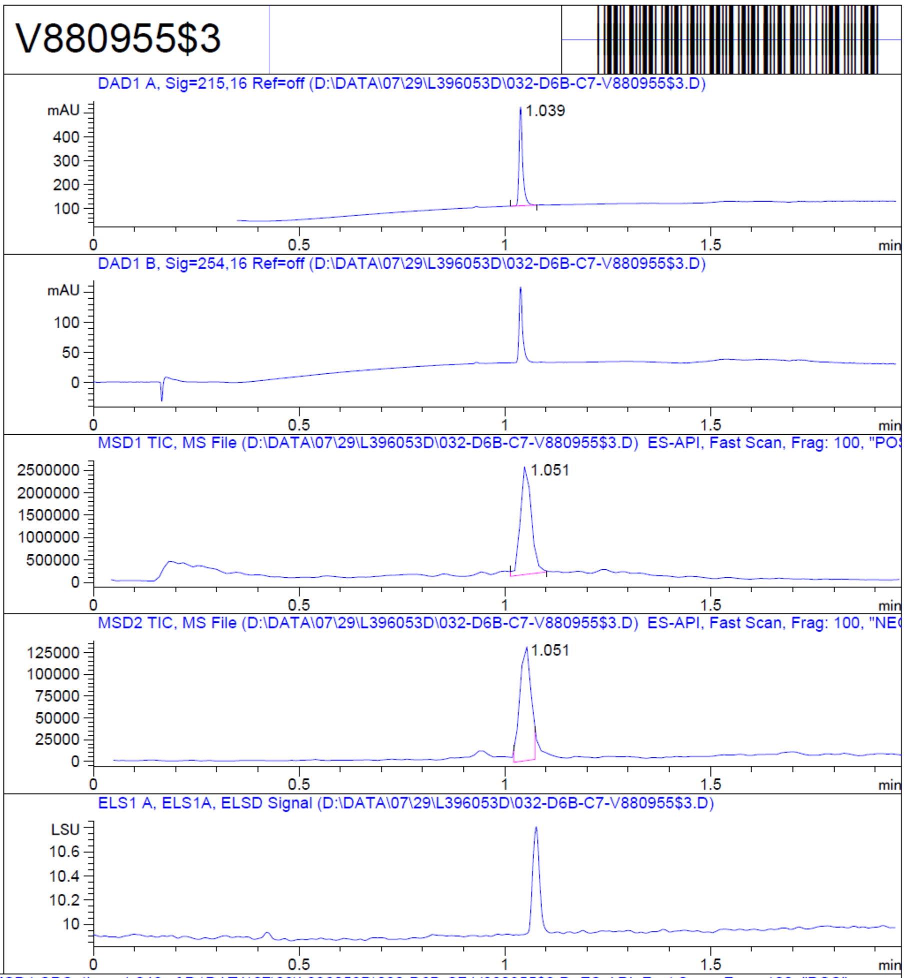

RT 1.051

RT 1.051

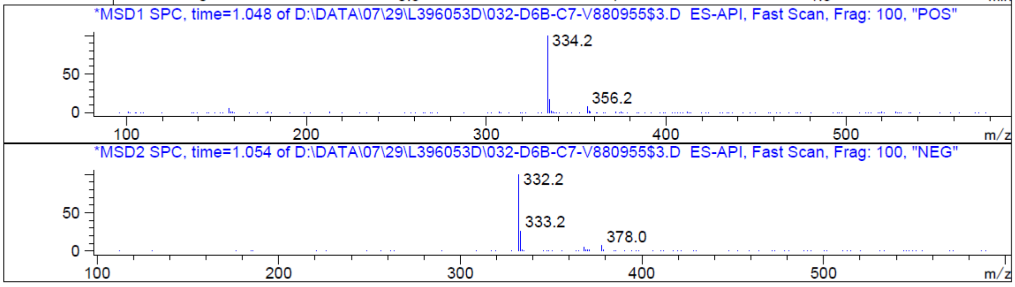

Inj.Date 7/27/2021 LB Acq. Method C:\Users\ -> ->

LCMS of compound **5(S)**

MaxPeak: 100.00%
Ret_Time: 1.230 min

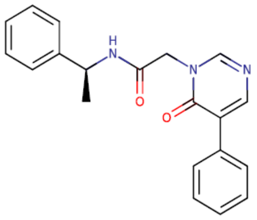

Mol Wt 333.38
Exact Mass 333.17

#	Time	Area%
1	1.230	100.00

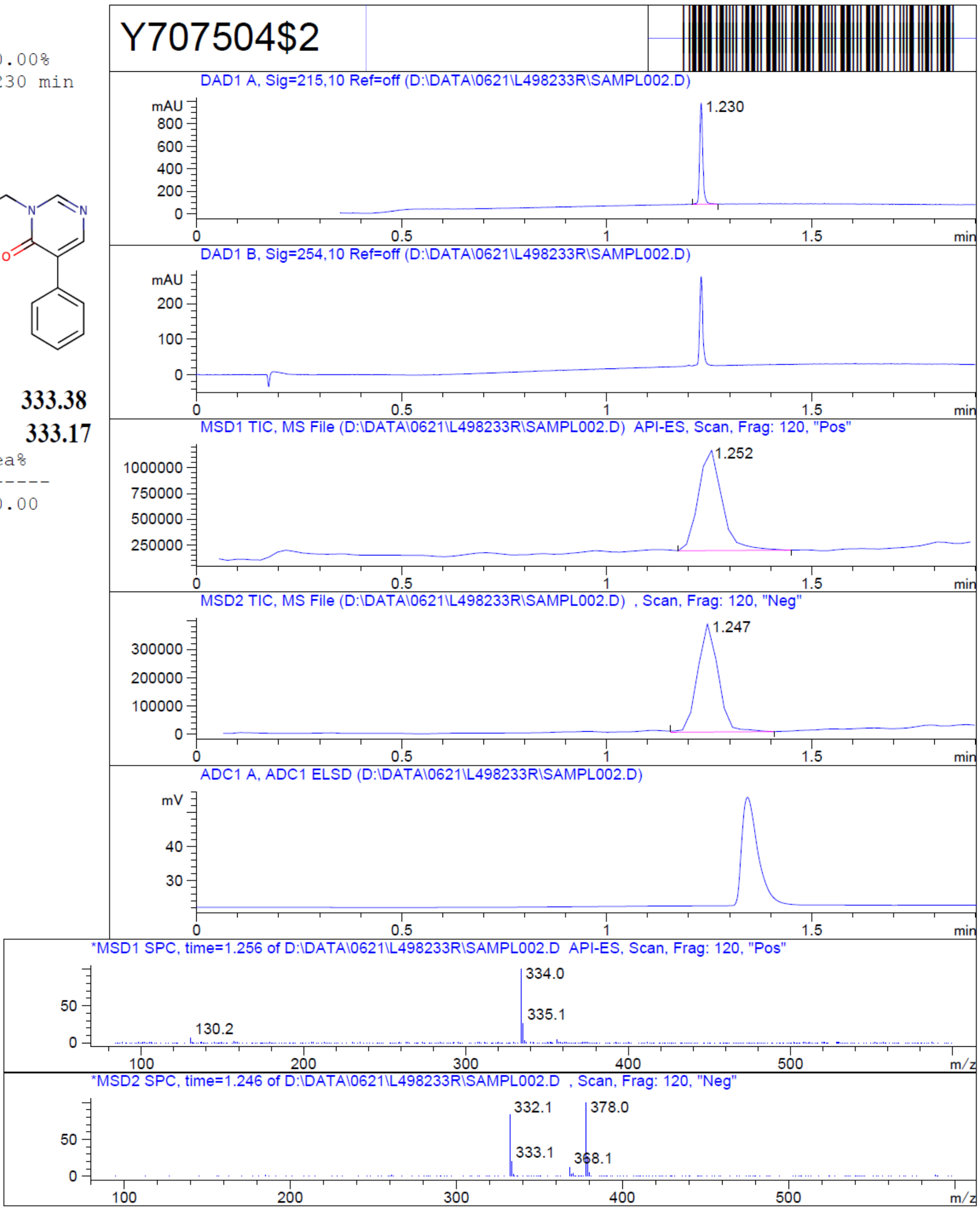

RT 1.252

RT 1.247

Inj.Date 6/21/2022 OA -VL- Acq. Method C:\HPCHEM\-> ->

HPLC of compound **JNJ(S)**

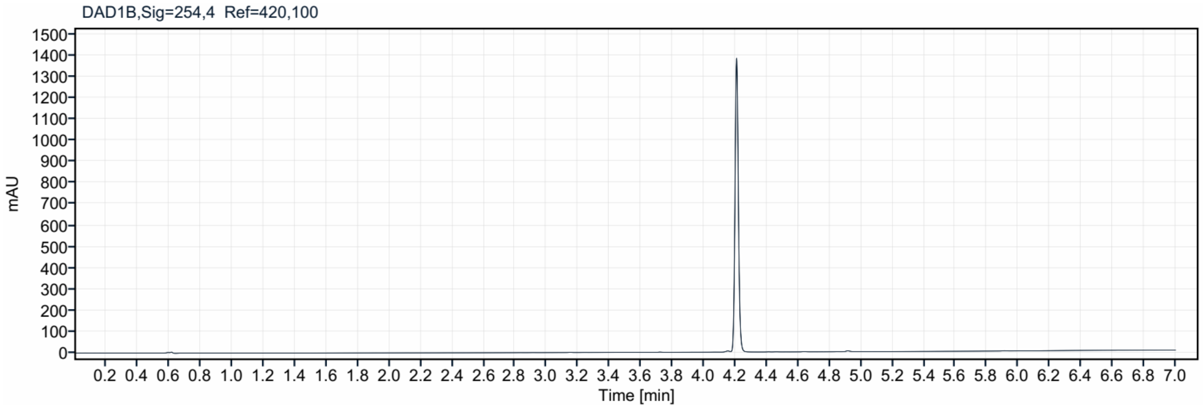

HPLC of compound **JNJ(R)**

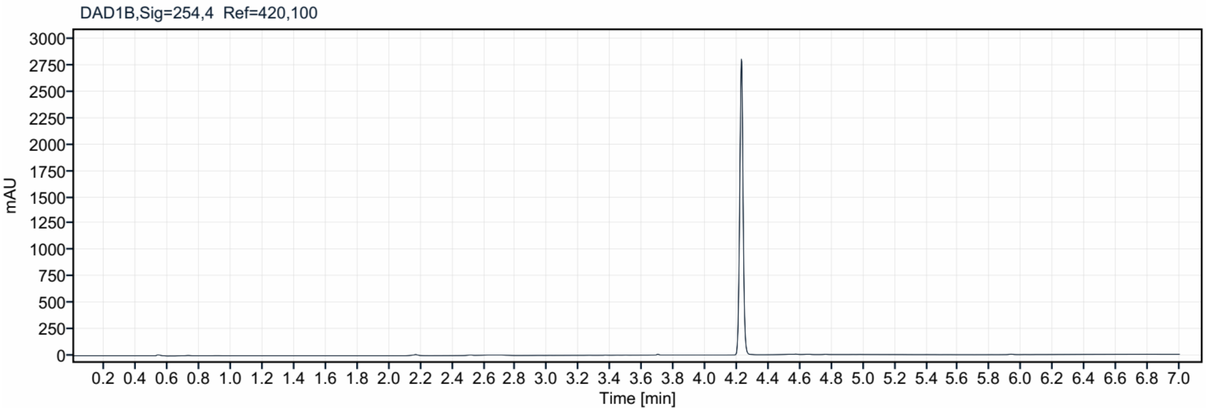

HPLC of compound **1.1(S)**

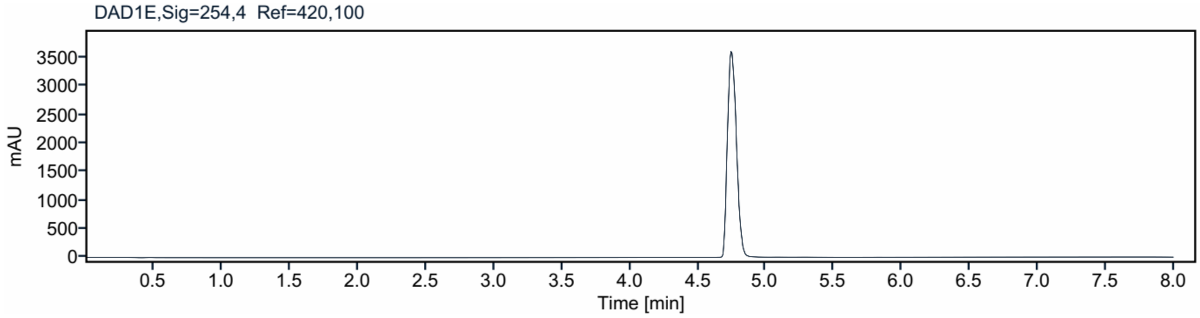

HPLC of compound **1.1(R)**

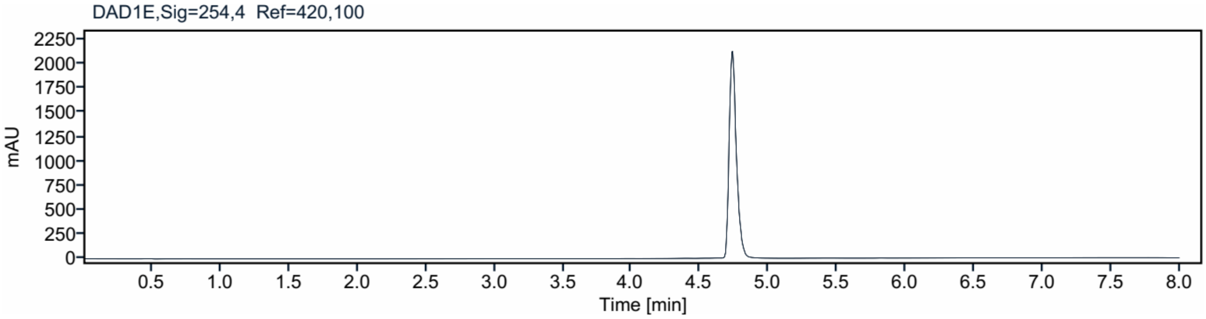

HPLC of compound **1.5(S)**

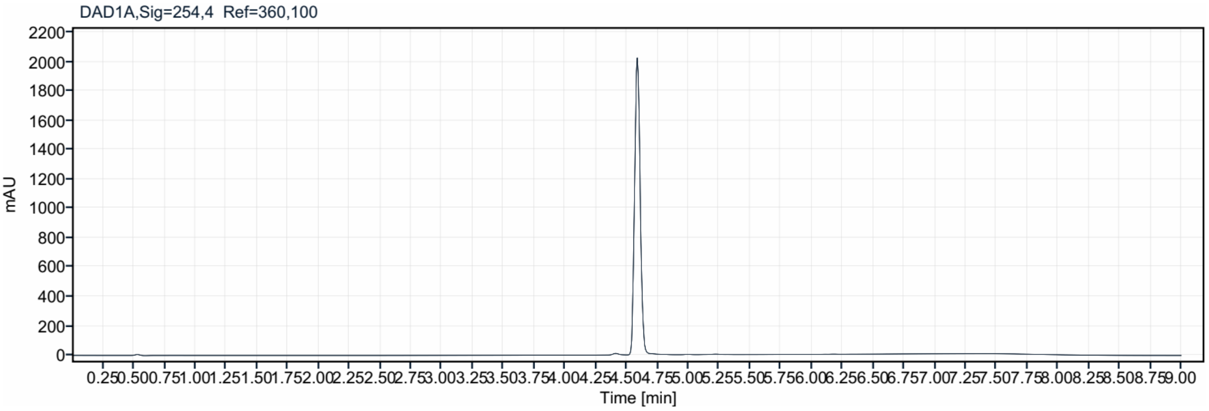

HPLC of compound **1.5(R)**

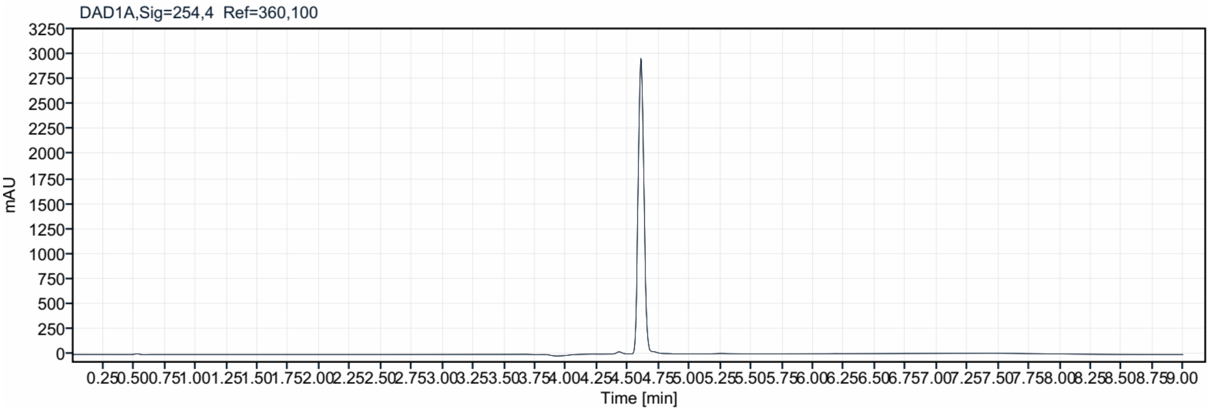

6. NMR spectra

^{1}H NMR of compound **1**

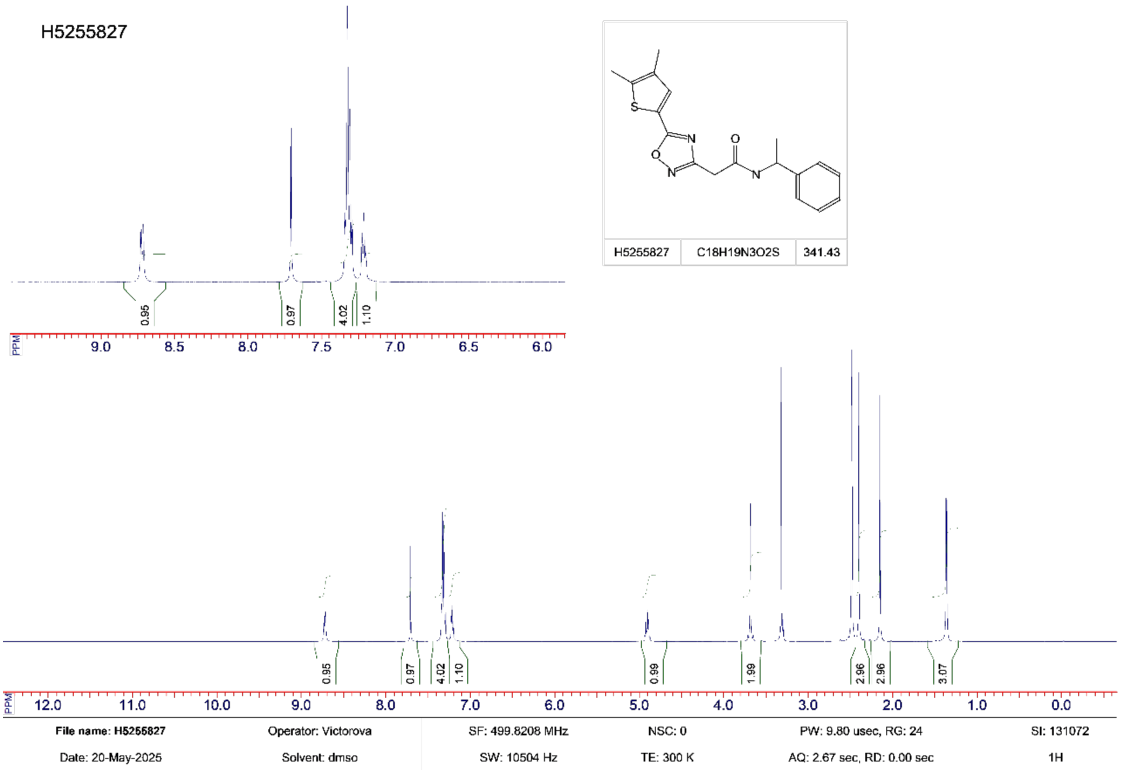

^{13}C NMR of compound **1**

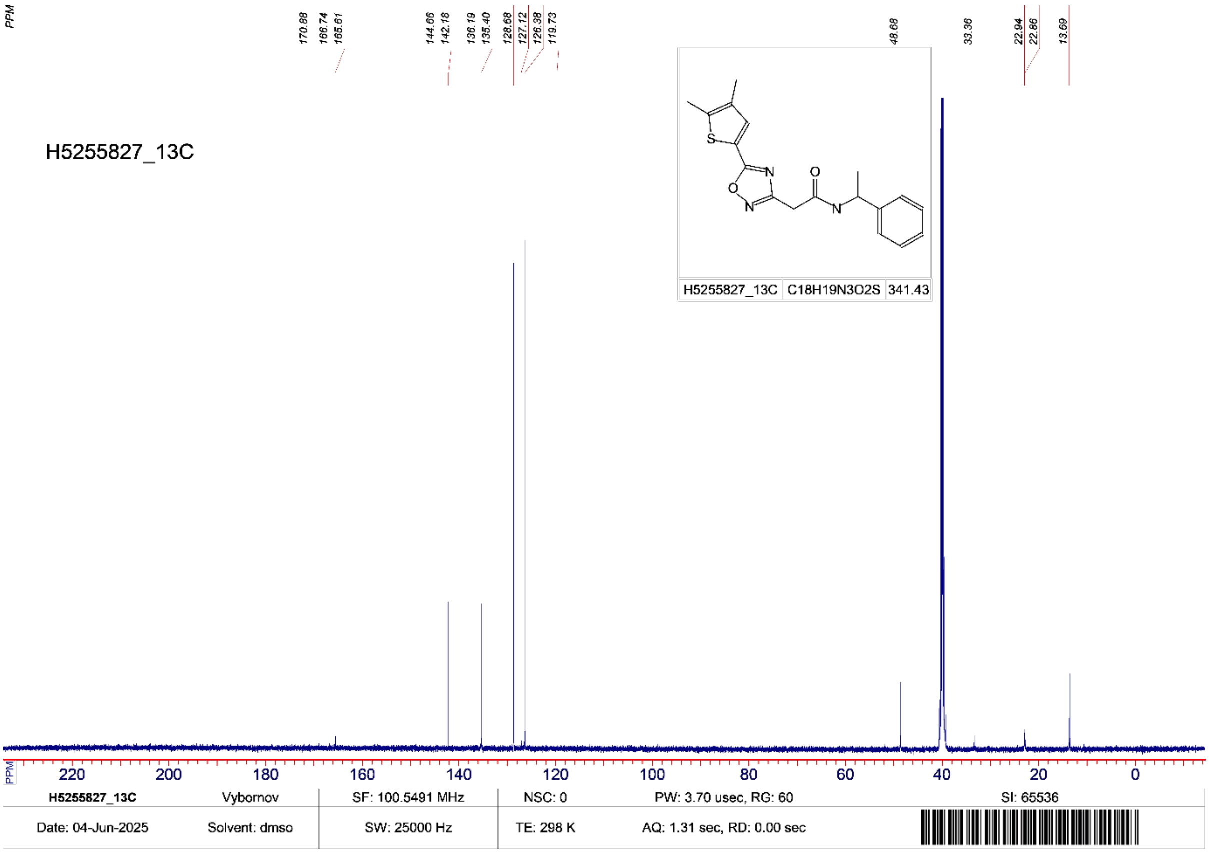

^{1}H NMR of compound **2**

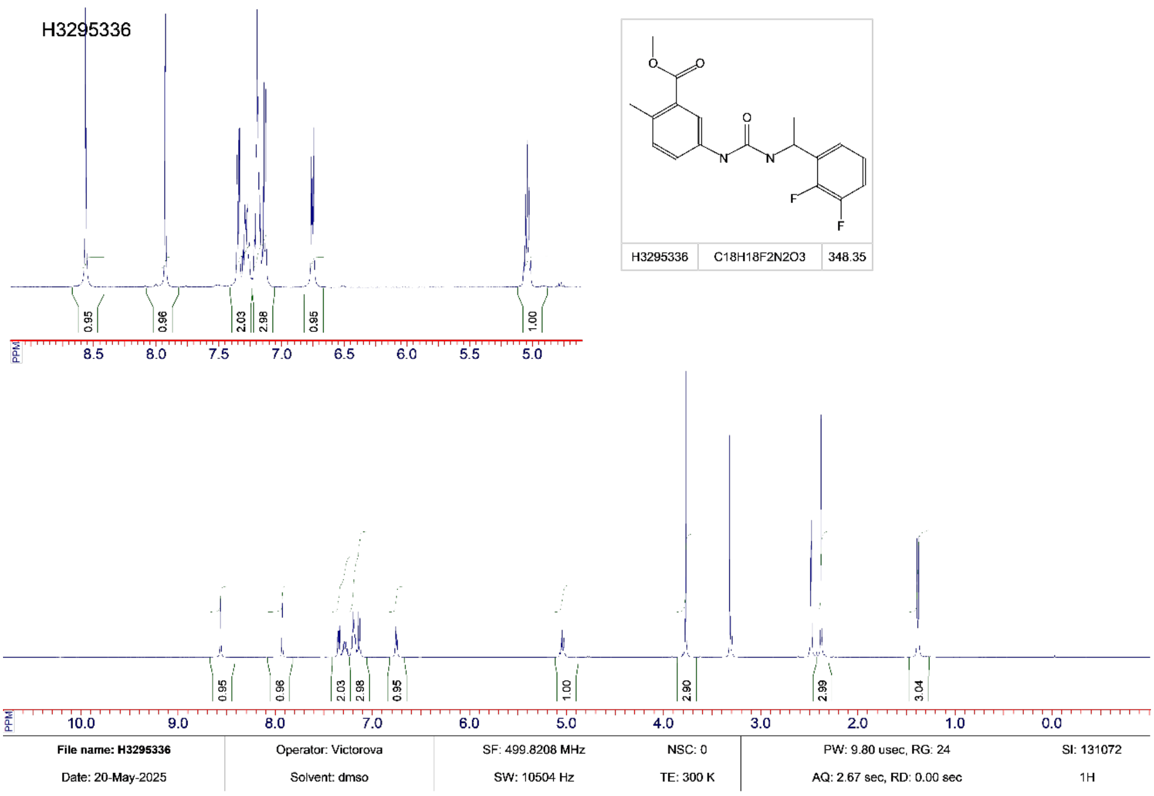

^{13}C NMR of compound **2**

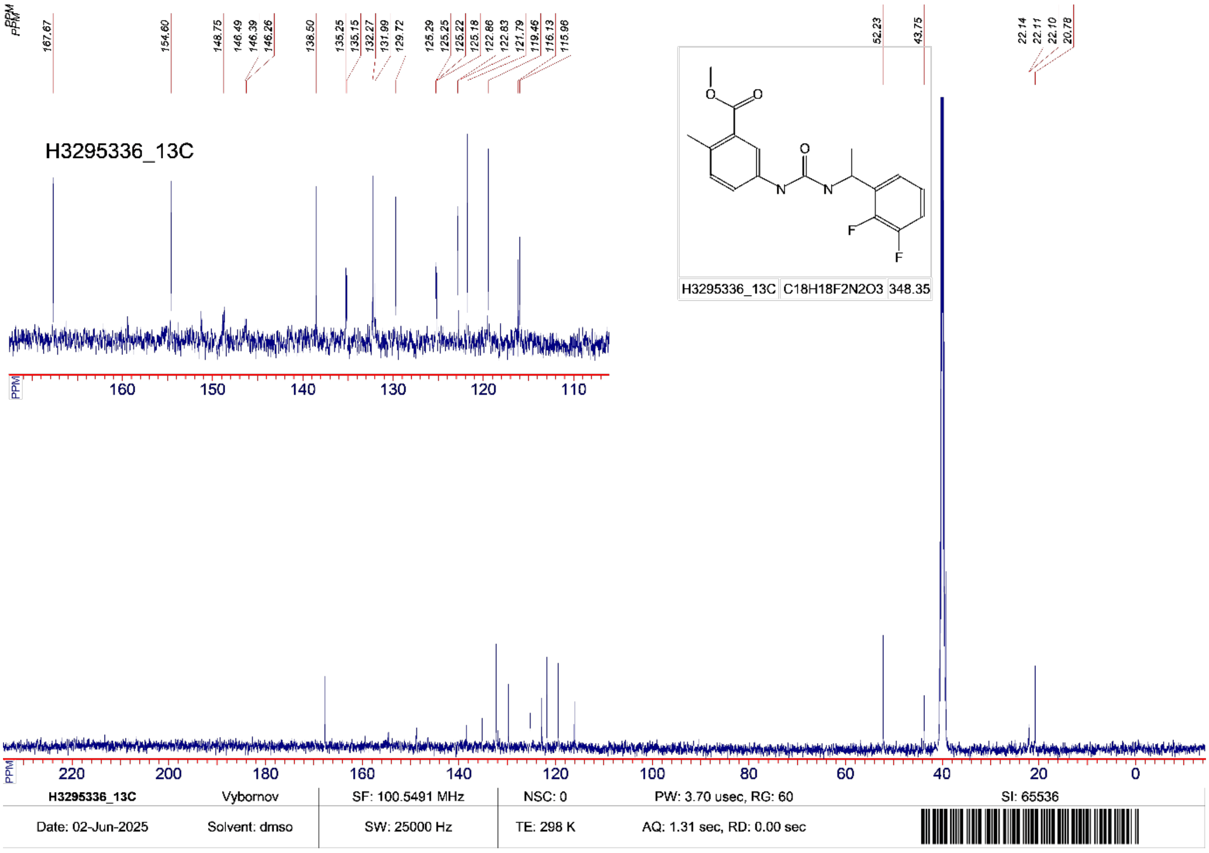

^{1}H NMR of compound **3**

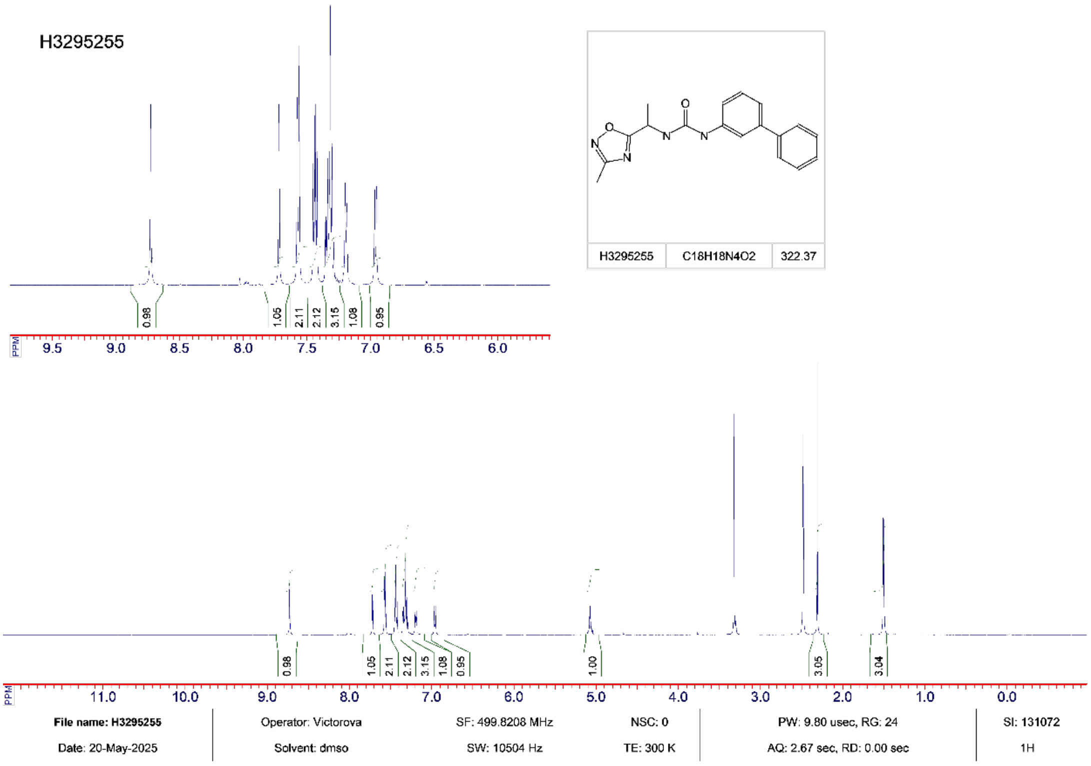

^{13}C NMR of compound **3**

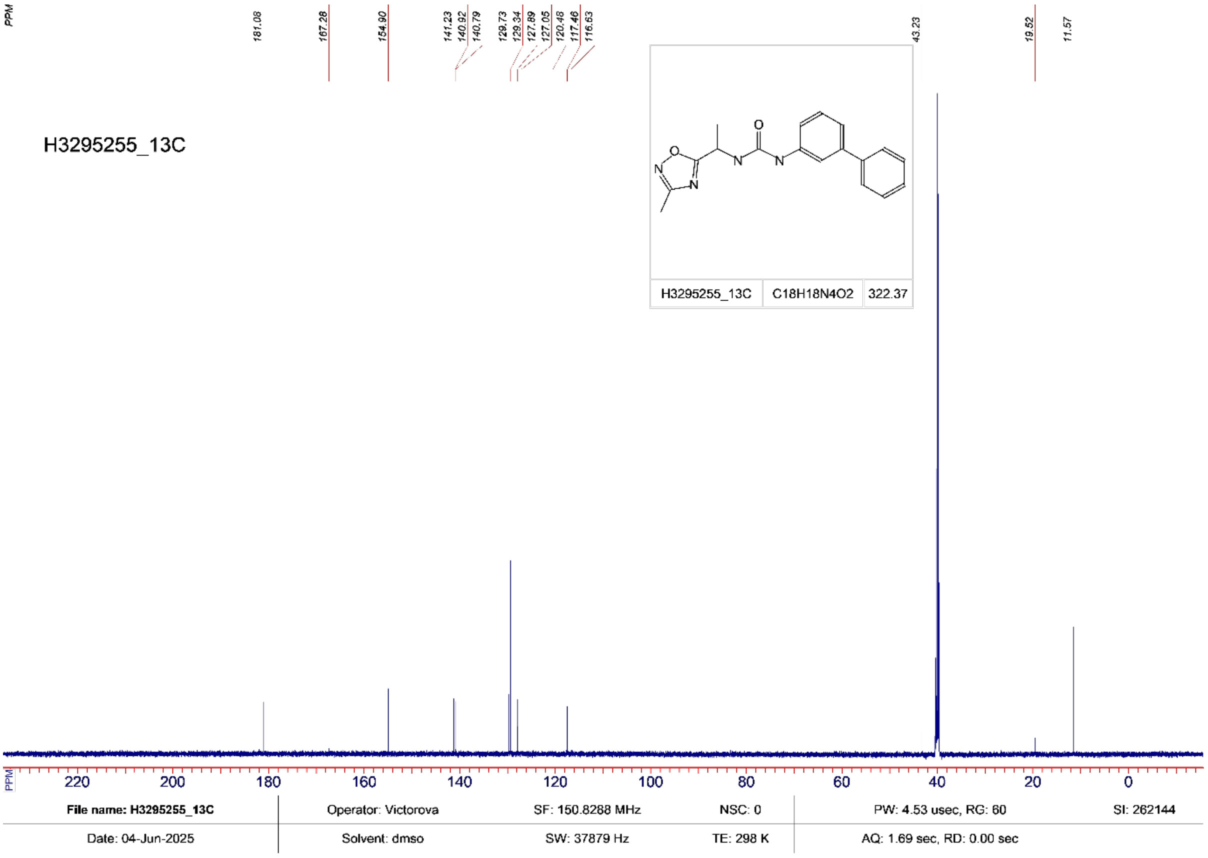

^{1}H NMR of compound **4**

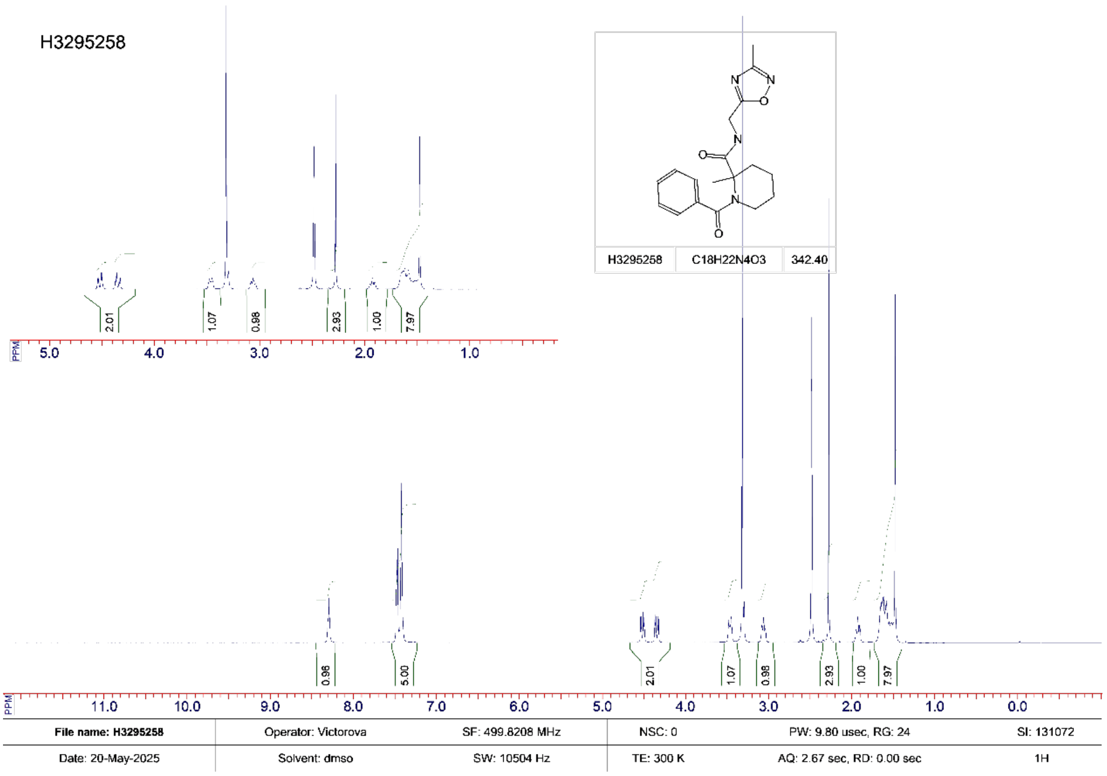

^{13}C NMR of compound **4**

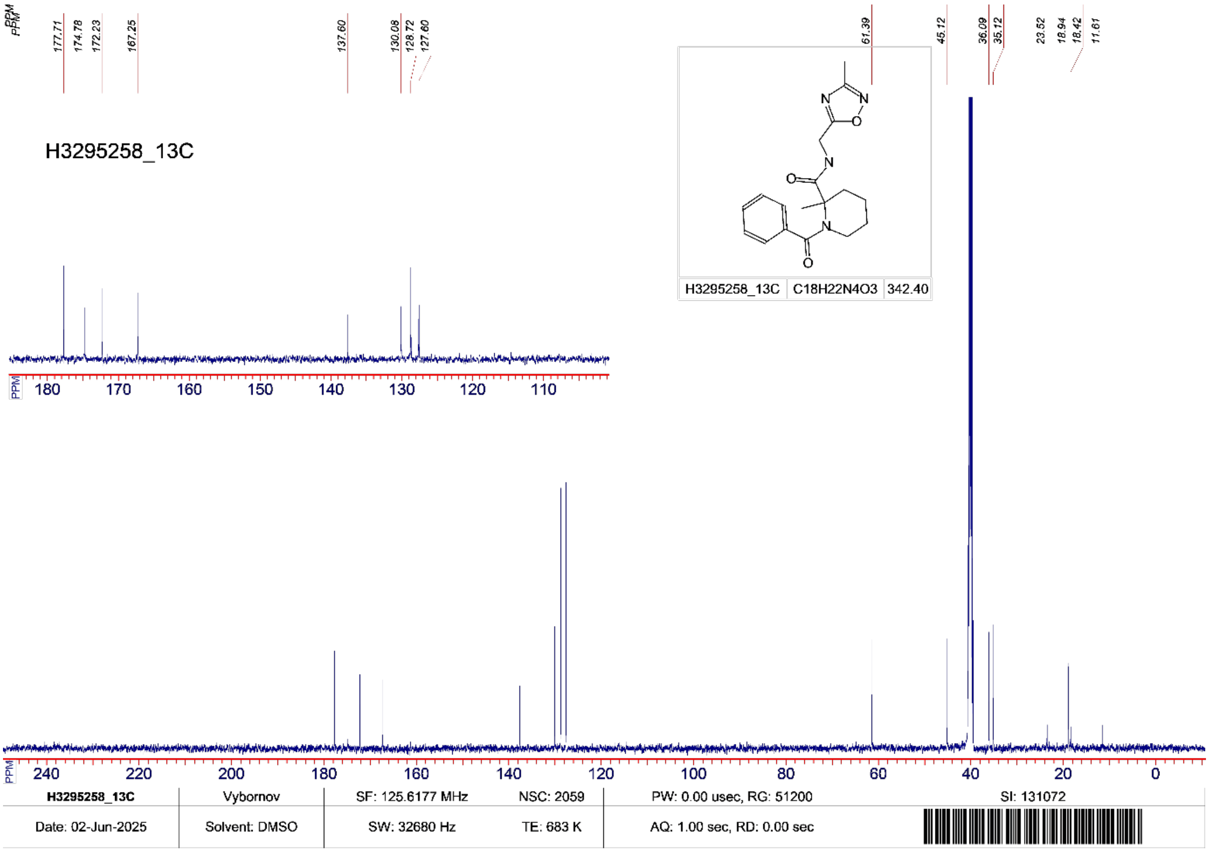

^{1}H NMR of compound **5(R)**

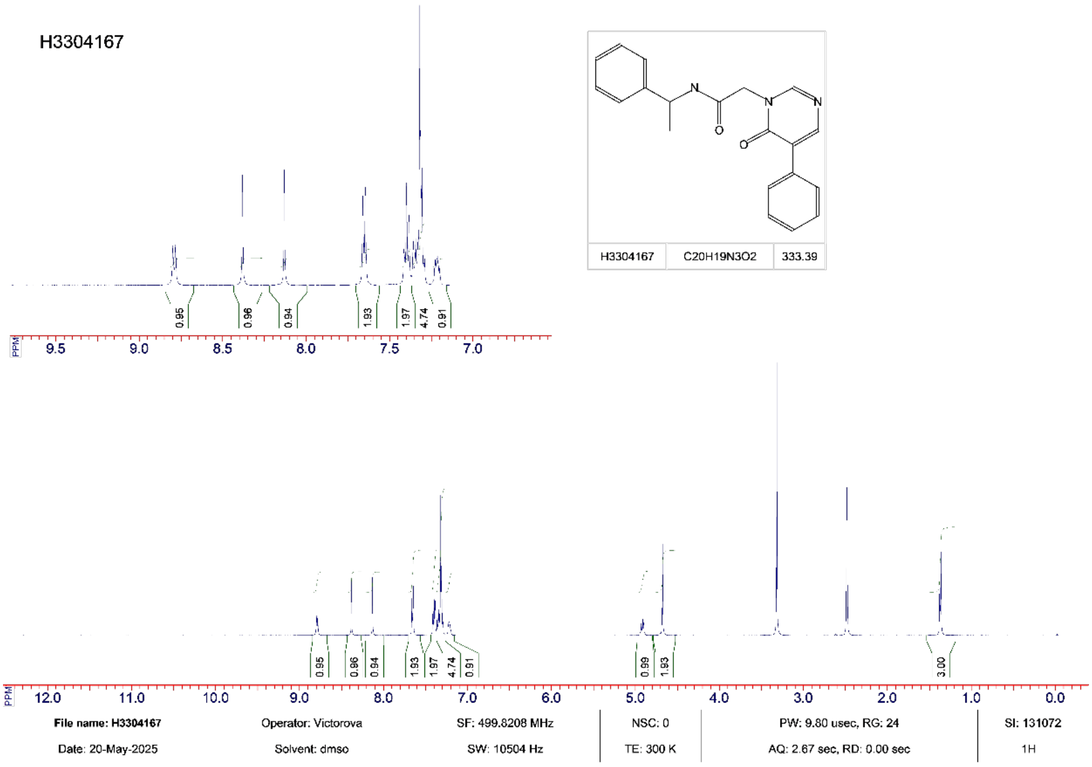

^{13}C NMR of compound **5(R)**

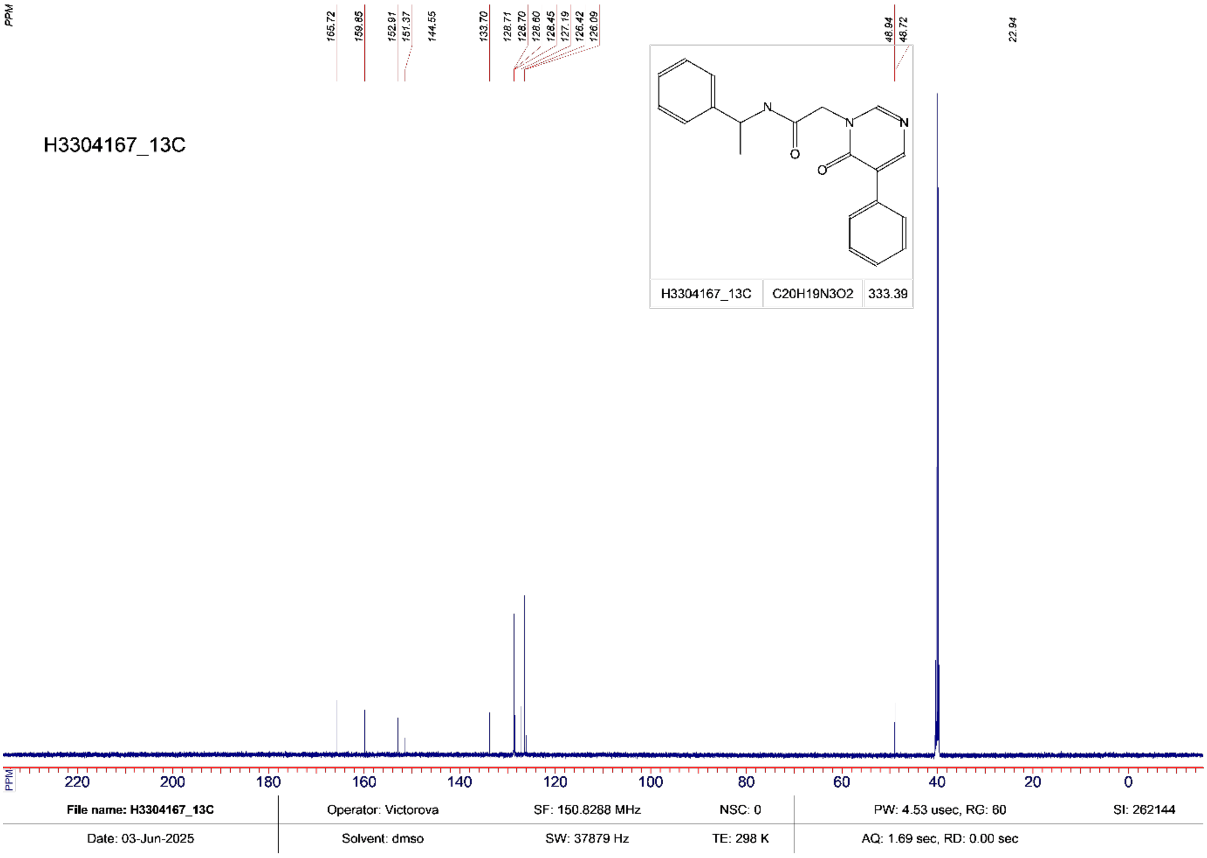

^{1}H NMR of compound **5(S)**

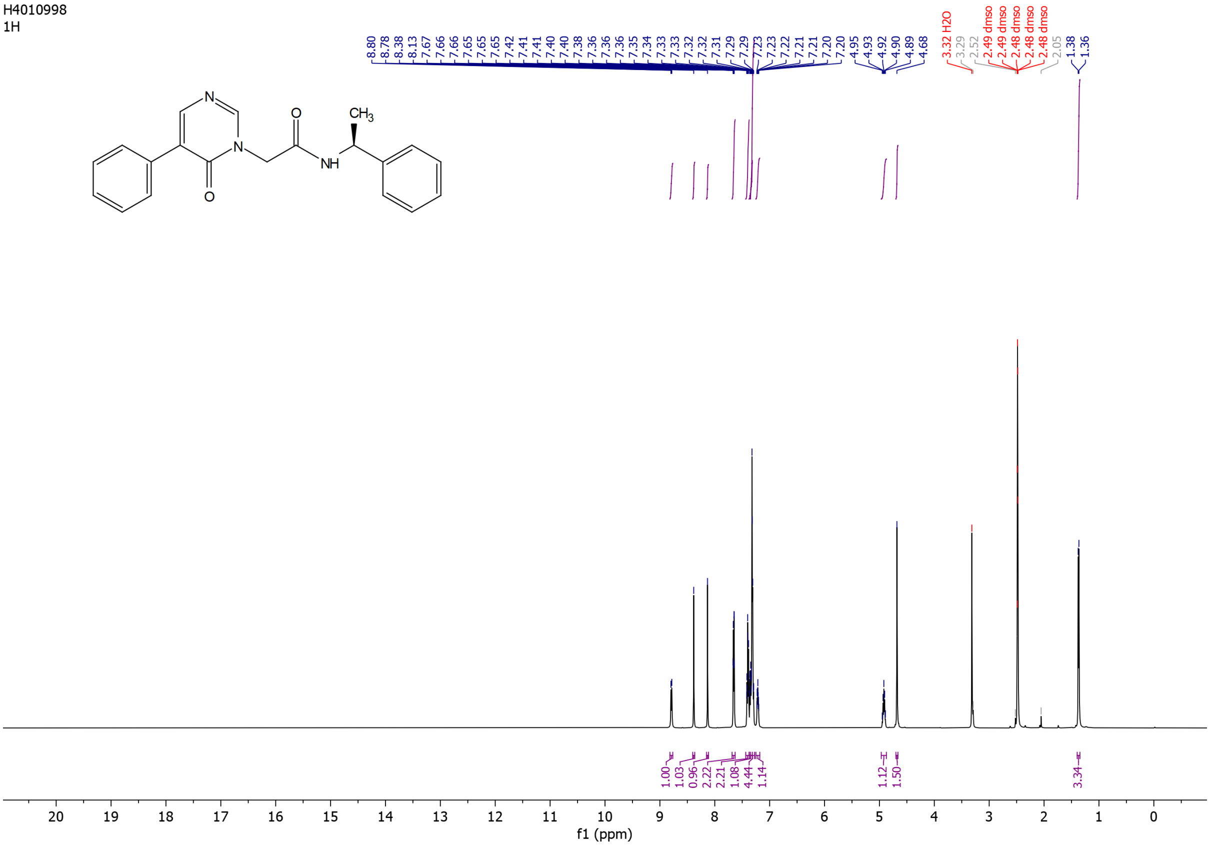

^{13}C NMR of compound **5(S)**

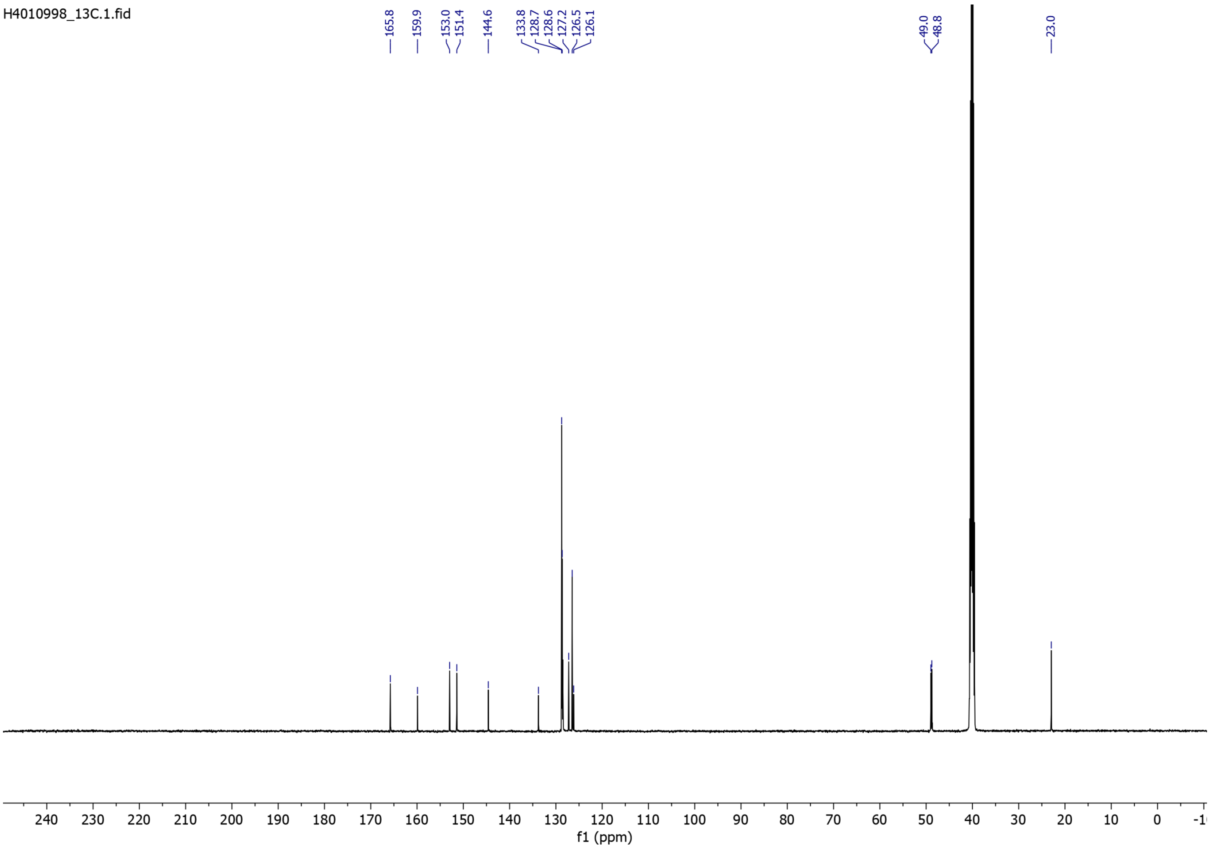

^{1}H NMR of compound **JNJ(S)** – CD_3OD

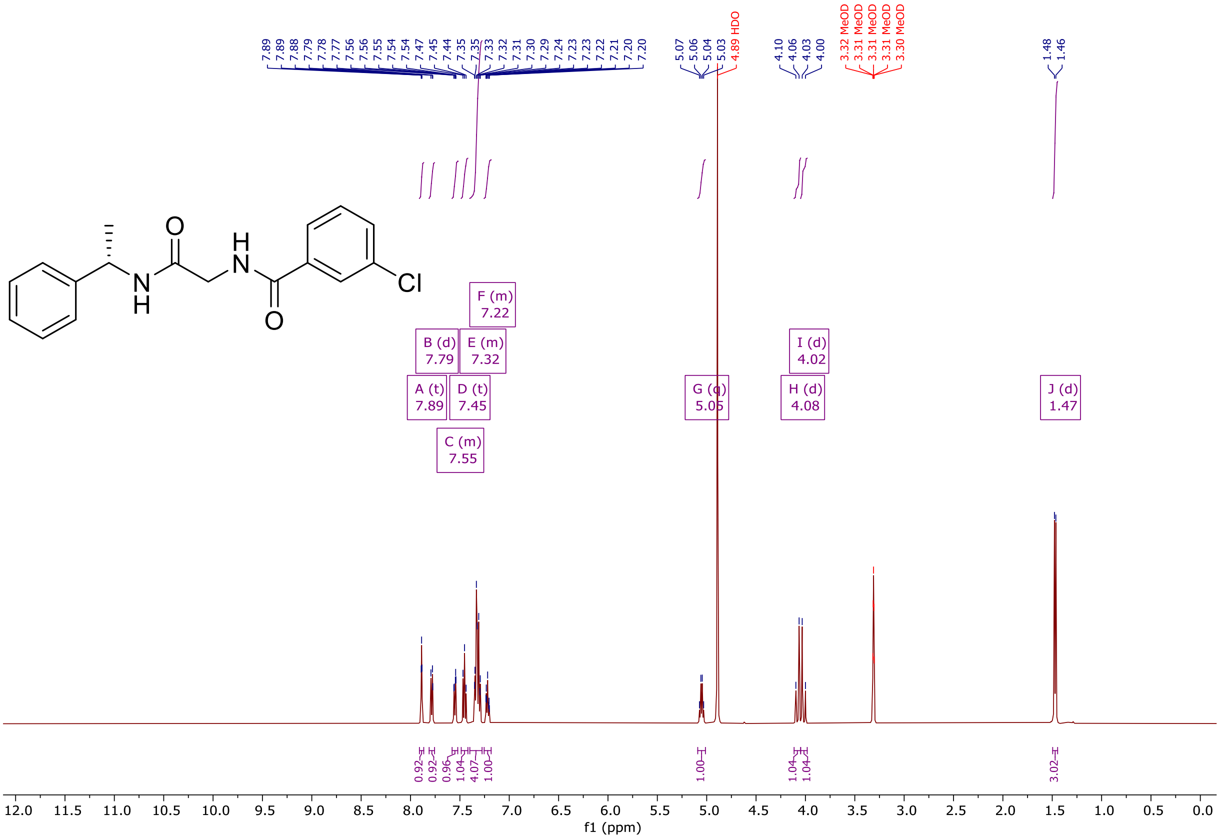

^{13}C NMR of compound **JNJ(S)** – CD_3OD

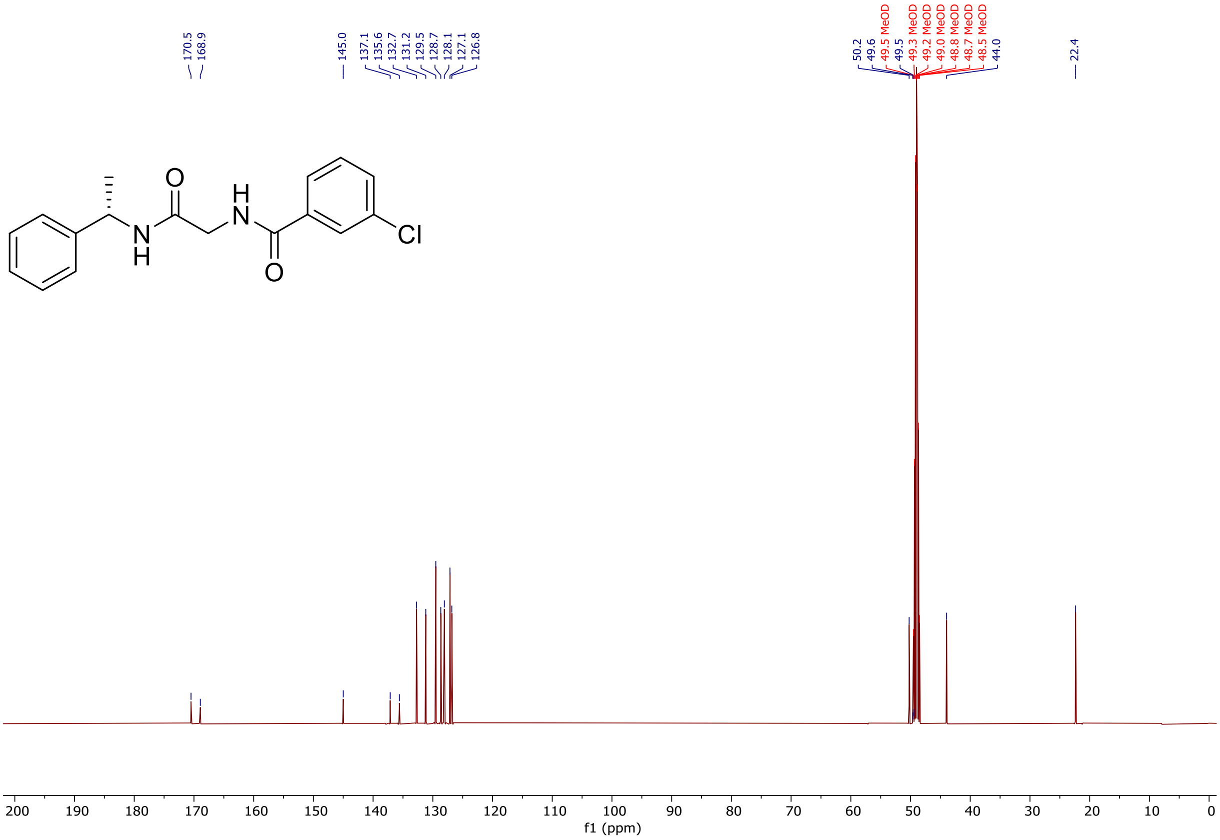

^{1}H NMR of compound **JNJ(R)** – CD_3OD

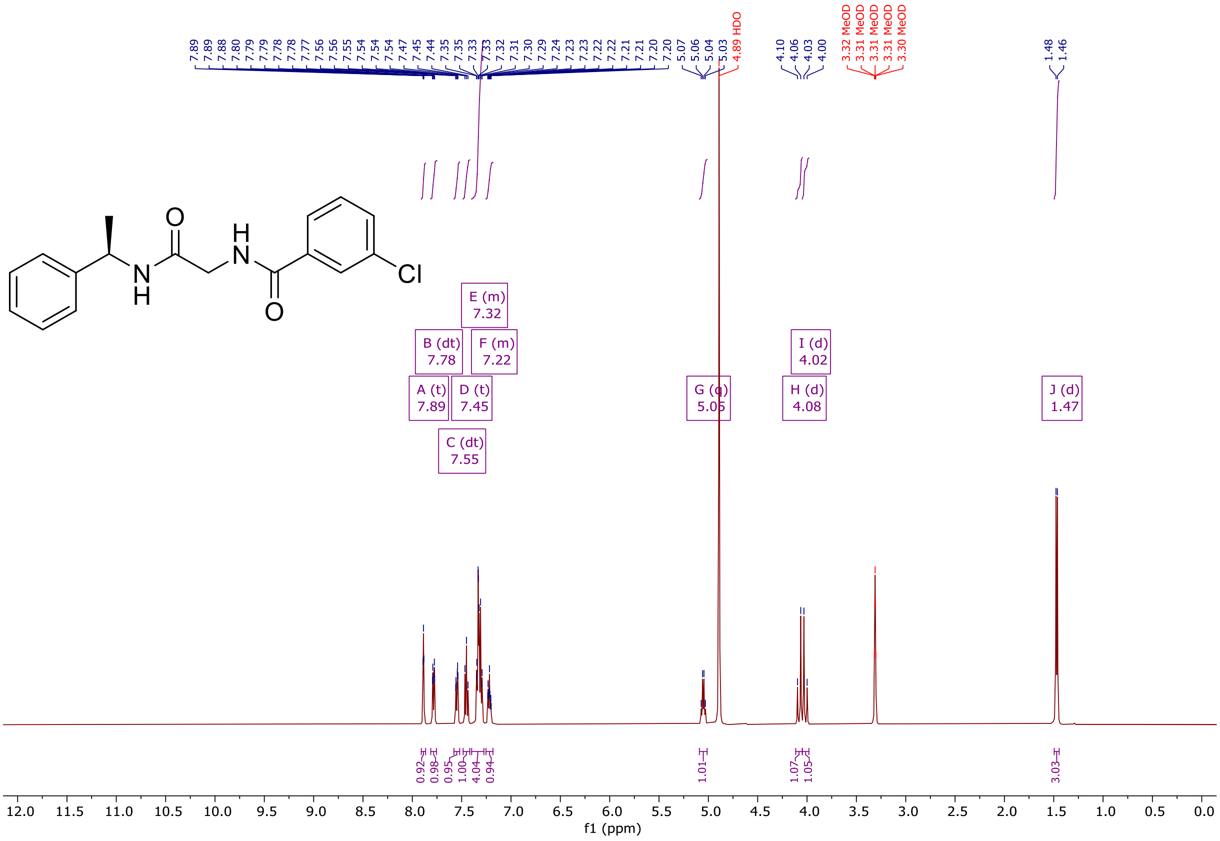

^{13}C NMR of compound **JNJ(R)** – CD_3OD

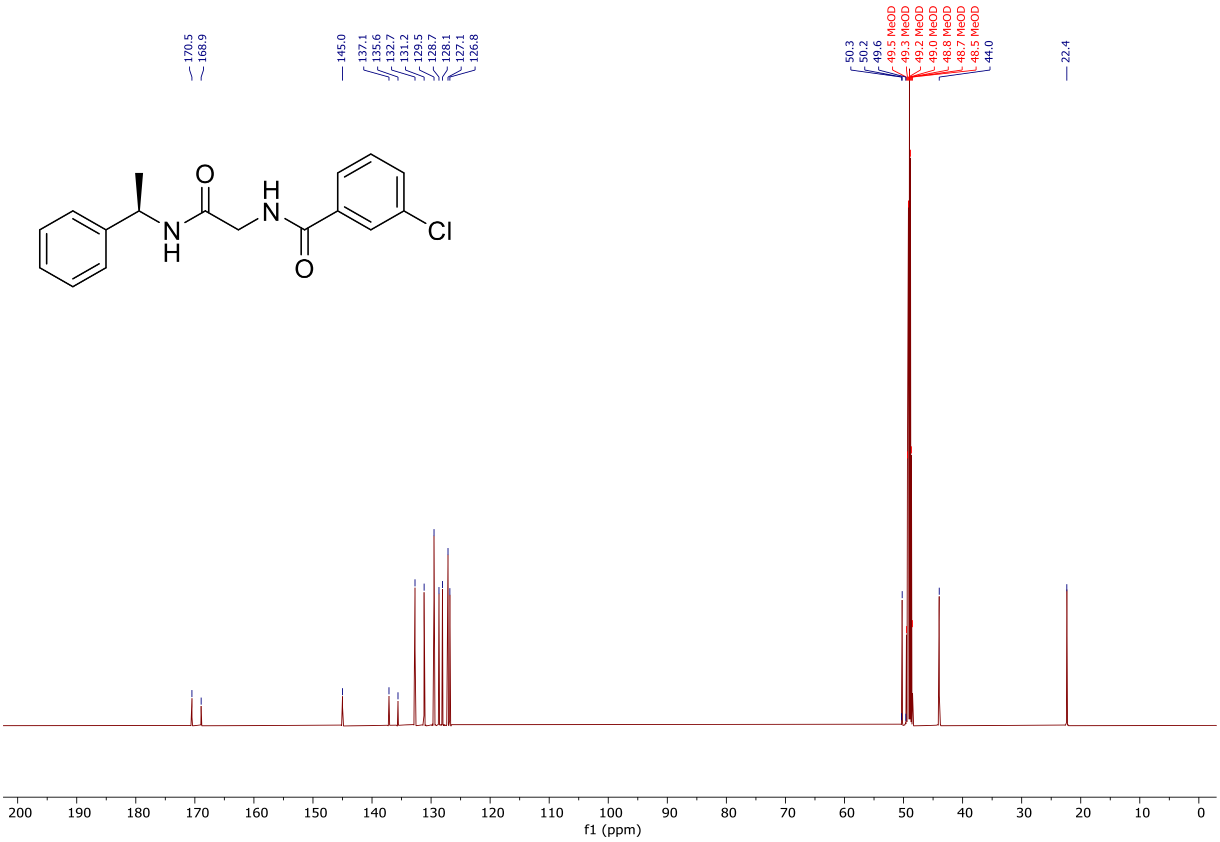

^{1}H NMR of compound **1.1(S)** – CD_3OD

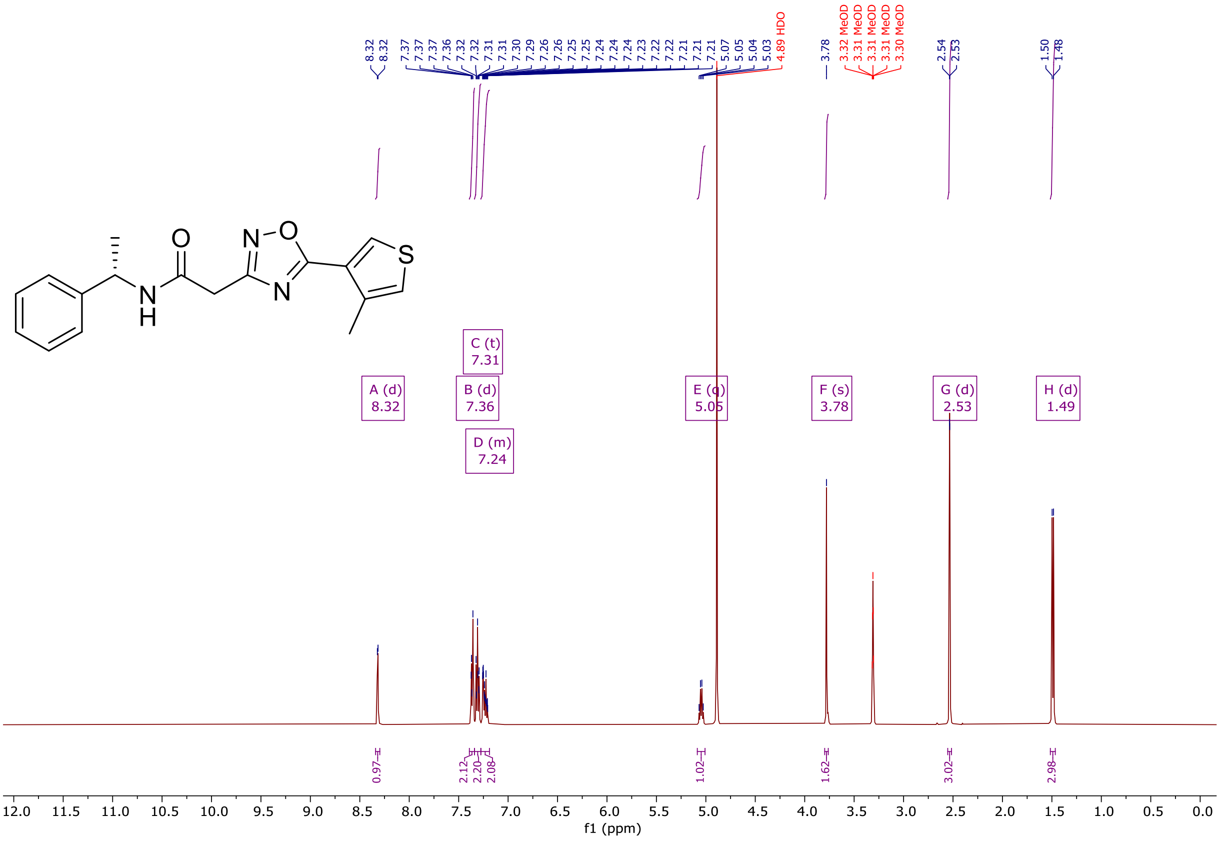

^{13}C NMR of compound **1.1(S)** – CD_3OD

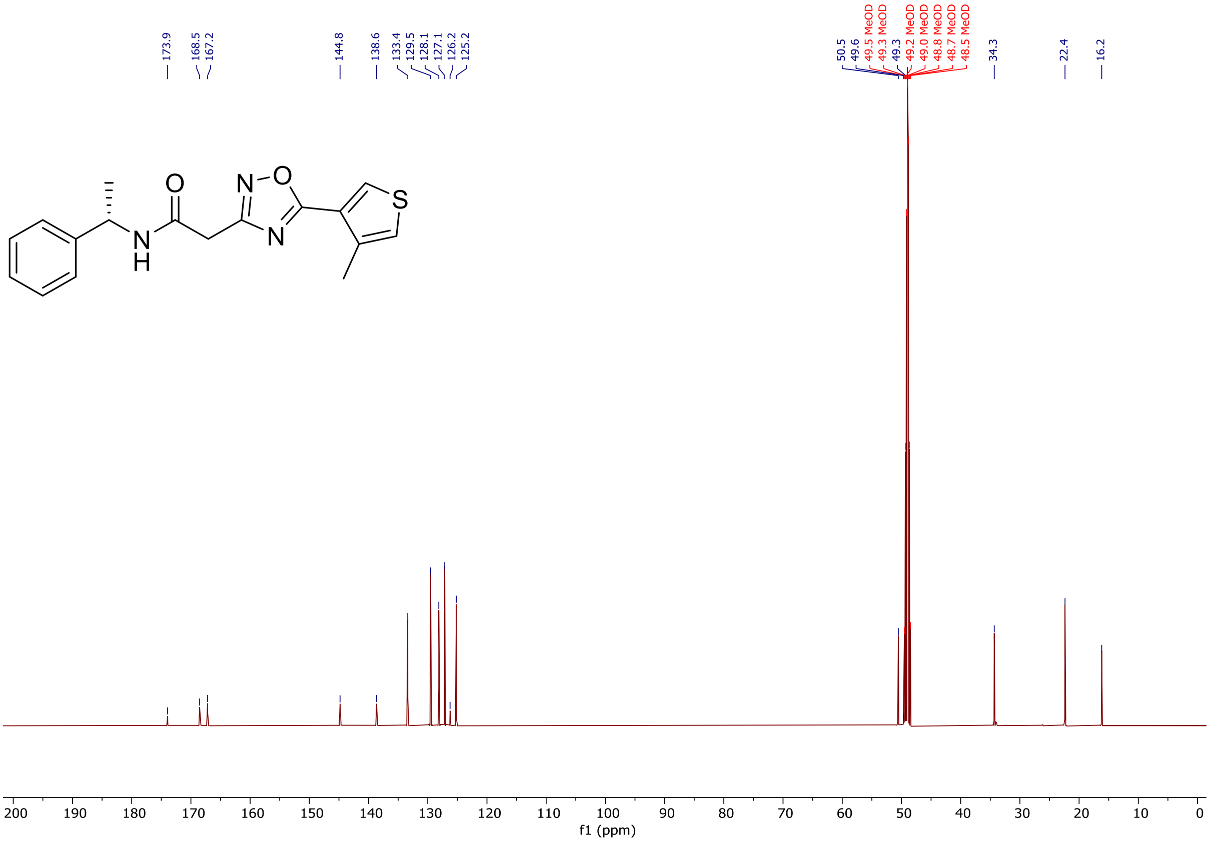

^{1}H NMR of compound **1.1(R)** – CD_3OD

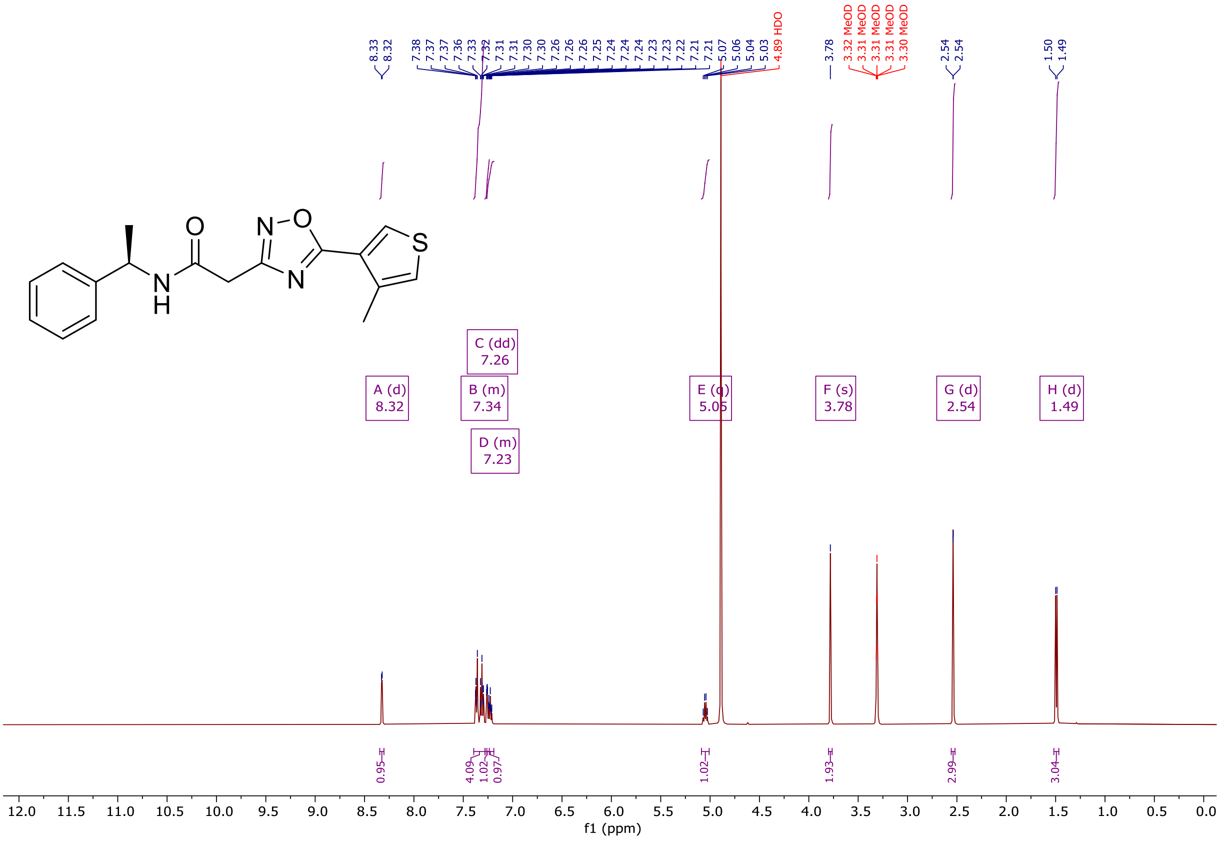

^{13}C NMR of compound **1.1(R)** – CD_3OD

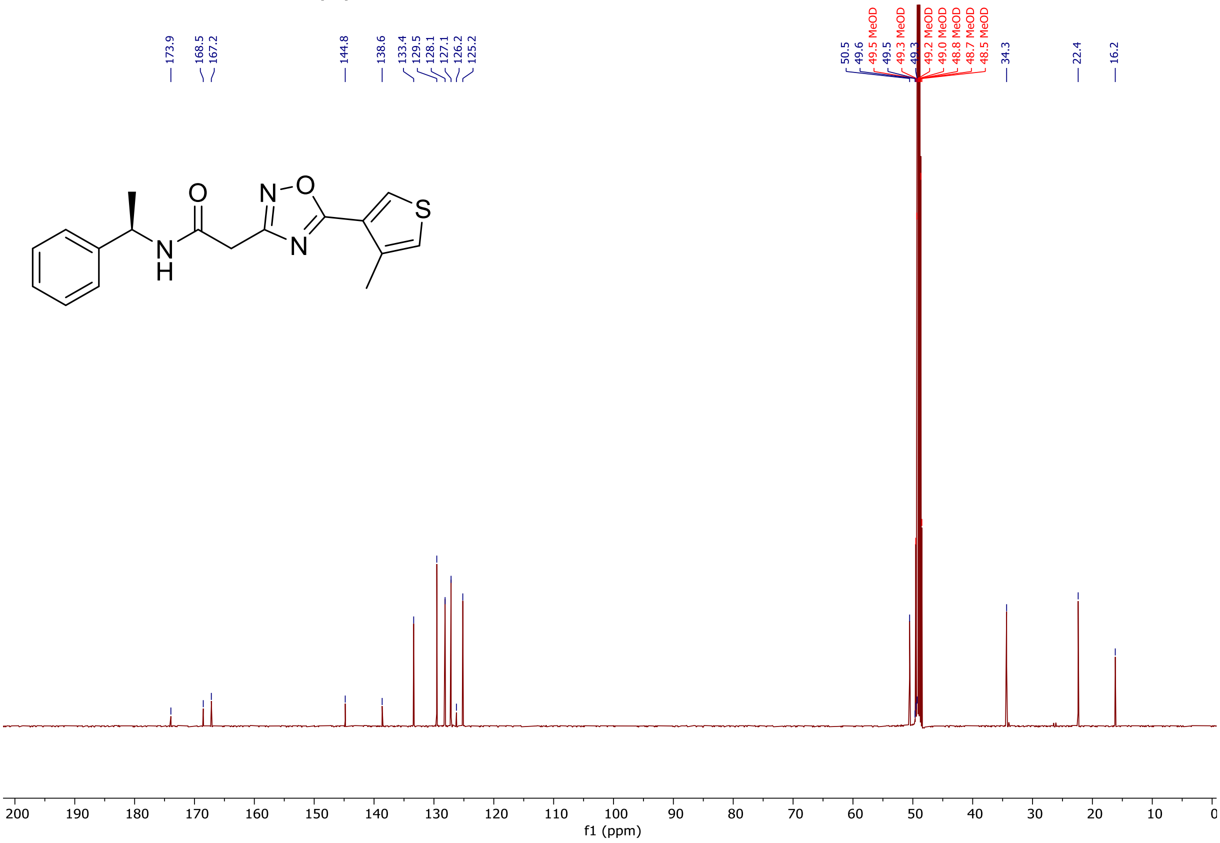

^{1}H NMR of compound **1.5(S)** – CD_3OD

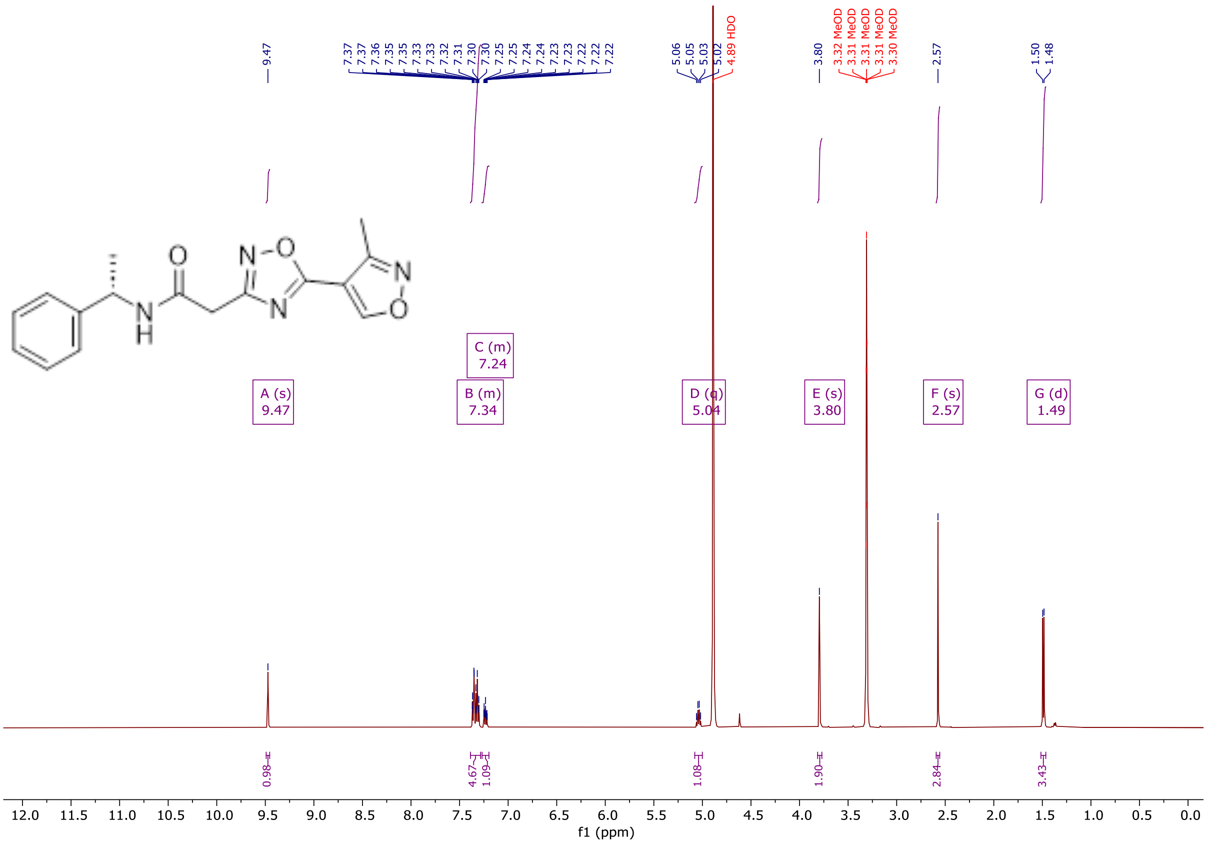

^{13}C NMR of compound **1.5(S)** – CD_3OD

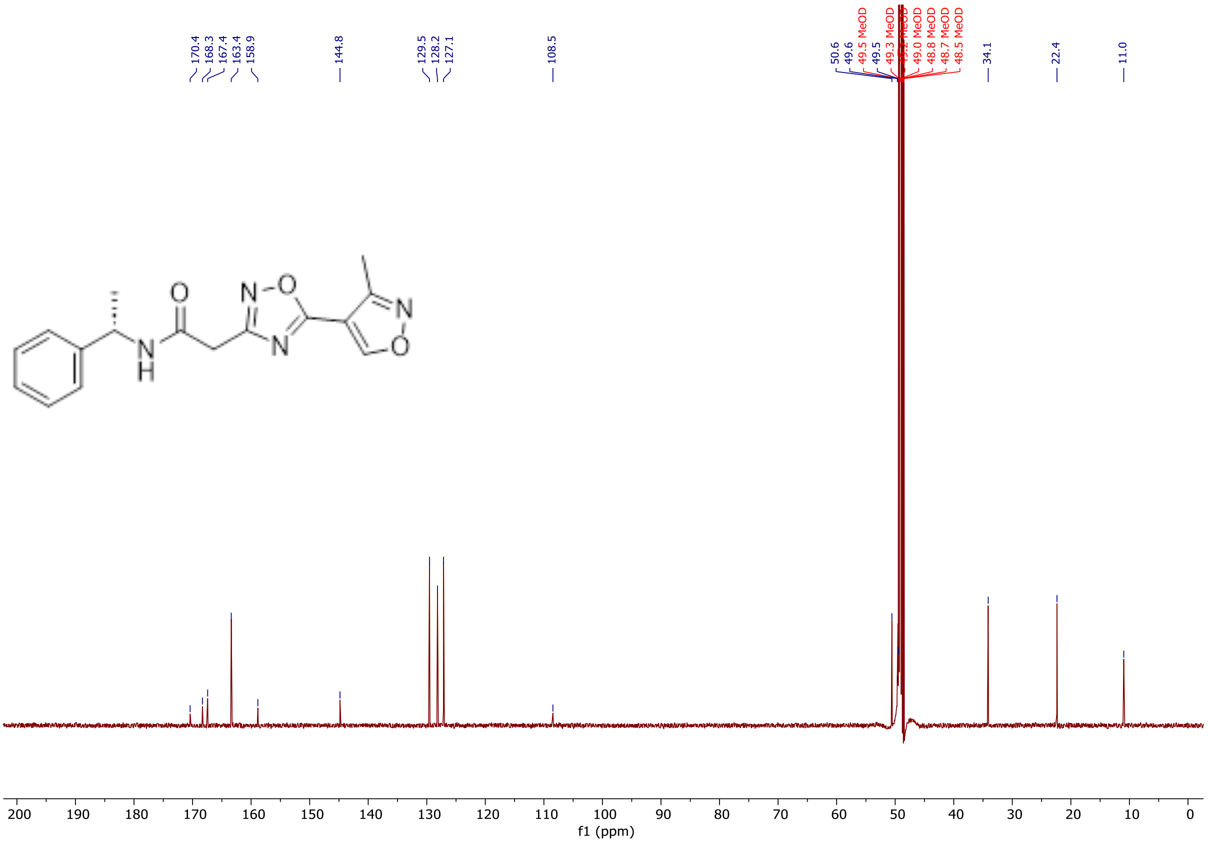

^{1}H NMR of compound **1.5(R)** – CD_3OD

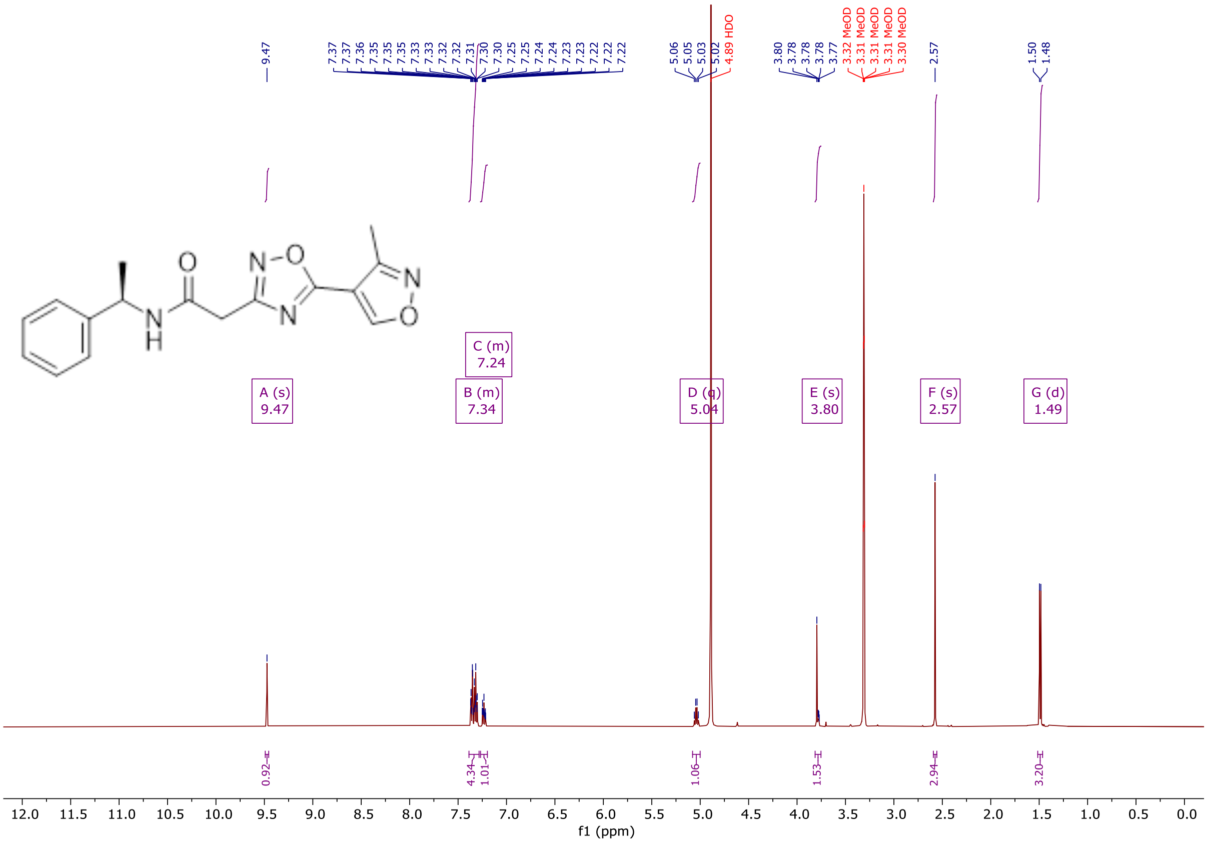

^{13}C NMR of compound **1.5(R)** – CD_3OD

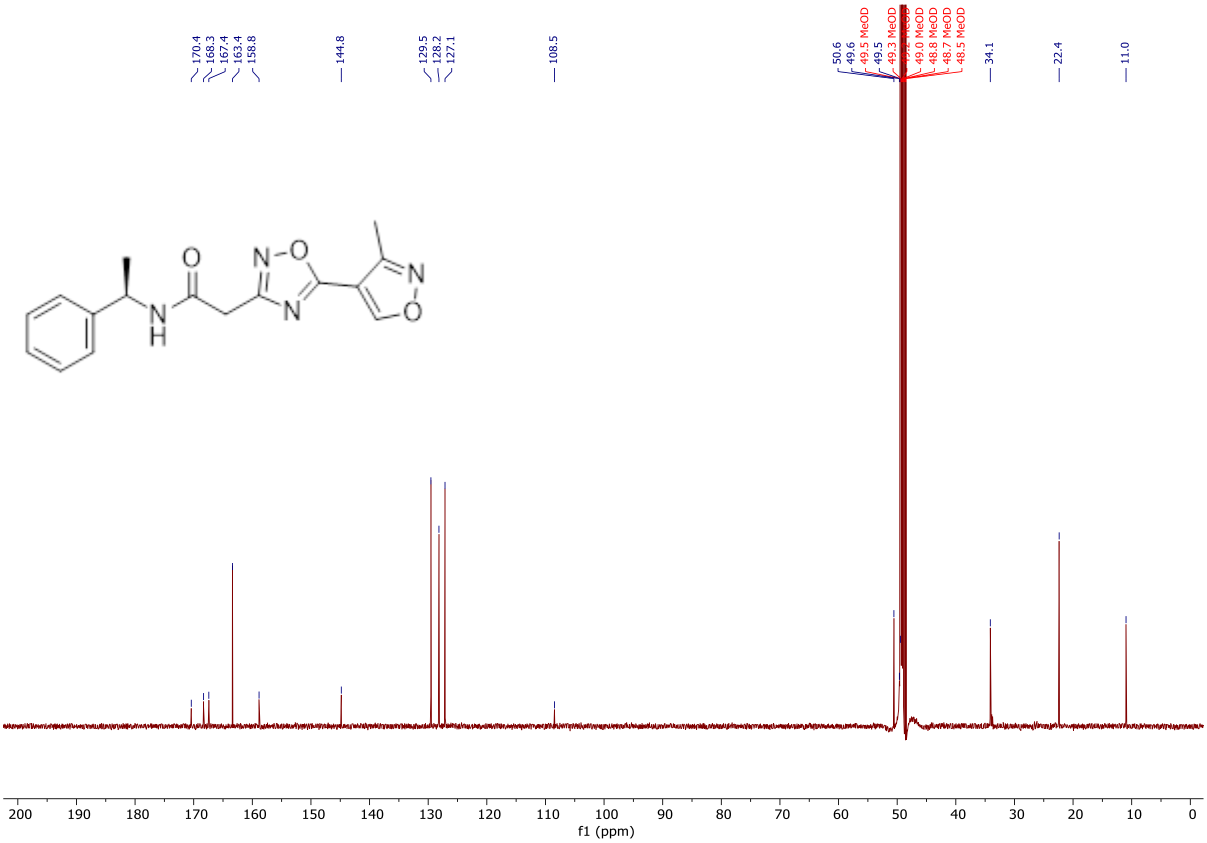

Supplementary References

(1) Barekatain, M.; Johansson, L. C.; Lam, J. H.; Chang, H.; Sadybekov, A. V.; Han, G. W.; Russo, J.; Bliesath, J.; Brice, N. L.; Carlton, M. B. L.; Saikatendu, K. S.; Sun, H.; Murphy, S. T.; Monenschein, H.; Schiffer, H. H.; Popov, P.; Lutomski, C. A.; Robinson, C. V.; Liu, Z.-J.; Hua, T.; Katritch, V.; Cherezov, V. Structural Insights into the High Basal Activity and Inverse Agonism of the Orphan Receptor GPR6 Implicated in Parkinson's Disease. *Sci. Signal.* **2024**, *17* (865), eado8741. https://doi.org/10.1126/scisignal.ado8741.

(2) Wang, J.-L.; Dou, X.-D.; Cheng, J.; Gao, M.-X.; Xu, G.-F.; Ding, W.; Ding, J.-H.; Li, Y.; Wang, S.-H.; Ji, Z.-W.; Zhao, X.-Y.; Huo, T.-Y.; Zhang, C.-F.; Liu, Y.-M.; Sha, X.-Y.; Gao, J.-R.; Zhang, W.-H.; Hao, Y.; Zhang, C.; Sun, J.-P.; Jiao, N.; Yu, X. Functional Screening and Rational Design of Compounds Targeting GPR132 to Treat Diabetes. *Nat. Metab.* **2023**, *5* (10), 1726–1746. https://doi.org/10.1038/s42255-023-00899-4.

(3) Lees, J. A.; Dias, J. M.; Rajamohan, F.; Fortin, J.-P.; O'Connor, R.; Kong, J. X.; Hughes, E. A. G.; Fisher, E. L.; Tuttle, J. B.; Lovett, G.; Kormos, B. L.; Unwalla, R. J.; Zhang, L.; Dechert Schmitt, A.-M.; Zhou, D.; Moran, M.; Stevens, K. A.; Fennell, K. F.; Varghese, A. E.; Maxwell, A.; Cote, E. E.; Zhang, Y.; Han, S. An Inverse Agonist of Orphan Receptor GPR61 Acts by a G Protein-Competitive Allosteric Mechanism. *Nat. Commun.* **2023**, *14* (1), 5938. https://doi.org/10.1038/s41467-023-41646-3.

(4) Liu, H.; Zhang, Q.; He, X.; Jiang, M.; Wang, S.; Yan, X.; Cheng, X.; Liu, Y.; Nan, F.-J.; Xu, H. E.; Xie, X.; Yin, W. Structural Insights into Ligand Recognition and Activation of the Medium-Chain Fatty Acid-Sensing Receptor GPR84. *Nat. Commun.* **2023**, *14* (1), 3271. https://doi.org/10.1038/s41467-023-38985-6.

(5) Duan, J.; Liu, Q.; Yuan, Q.; Ji, Y.; Zhu, S.; Tan, Y.; He, X.; Xu, Y.; Shi, J.; Cheng, X.; Jiang, H.; Eric Xu, H.; Jiang, Y. Insights into Divalent Cation Regulation and G13-Coupling of Orphan Receptor GPR35. *Cell Discov.* **2022**, *8* (1), 135. https://doi.org/10.1038/s41421-022-00499-8.

(6) Dvorak, C. A.; Coate, H.; Nepomuceno, D.; Wennerholm, M.; Kuei, C.; Lord, B.; Woody, D.; Bonaventure, P.; Liu, C.; Lovenberg, T.; Carruthers, N. I. Identification and SAR of Glycine Benzamides as Potent Agonists for the GPR139 Receptor. *ACS Med. Chem. Lett.* **2015**, *6* (9), 1015–1018. https://doi.org/10.1021/acsmedchemlett.5b00247.

(7) Tolmachev, A.; Bogolubsky, A. V.; Pipko, S. E.; Grishchenko, A. V.; Ushakov, D. V.; Zhemera, A. V.; Viniychuk, O. O.; Konovets, A. I.; Zaporozhets, O. A.; Mykhailiuk, P. K.; Moroz, Y. S. Expanding Synthesizable Space of Disubstituted 1,2,4-Oxadiazoles. *ACS Comb. Sci.* **2016**, *18* (10), 616–624. https://doi.org/10.1021/acscombsci.6b00103.

(8) Bogolubsky, A. V.; Ryabukhin, S. V.; Pipko, S. E.; Lukin, O.; Shivanyuk, A.; Mykytenko, D.; Tolmachev, A. A Facile Synthesis of Unsymmetrical Ureas. *Tetrahedron* **2011**, *67* (20), 3619–3623. https://doi.org/10.1016/j.tet.2011.03.101.
