# Supplementary material for: Ultra-large virtual screening unveils potent agonists of the neuromodulatory orphan receptor GPR139
Source: Nat Commun. 2025 Dec 9;17:129. doi: 10.1038/s41467-025-66845-y (PMC12775434; doi:10.1038/s41467-025-66845-y)
Supplement: Supplementary file 2 — Description of Additional Supplementary Files [file 41467_2025_66845_MOESM2_ESM.pdf]

Description of Additional Supplementary File

Supplementary Data 1: (IC_GPR139_SupplementaryData_1.tar.gz): Docked poses of compound 1.1, compounds 1-5, cryo-EM structure of GPR139 bound to JNJ-63533054 pose 2.
